# Accuracy of a Wrist-Worn Wearable Device for Monitoring Heart Rates in Hospital Inpatients: A Prospective Observational Study

**DOI:** 10.2196/jmir.6025

**Published:** 2016-09-20

**Authors:** Ryan R Kroll, J Gordon Boyd, David M Maslove

**Affiliations:** ^1^ Department of Medicine Queen's University Kingston, ON Canada; ^2^ Department of Critical Care Medicine Queen's University Kingston, ON Canada

**Keywords:** biometry/instrumentation, clothing, monitoring, physiologic, informatics, clinical trial

## Abstract

**Background:**

As the sensing capabilities of wearable devices improve, there is increasing interest in their application in medical settings. Capabilities such as heart rate monitoring may be useful in hospitalized patients as a means of enhancing routine monitoring or as part of an early warning system to detect clinical deterioration.

**Objective:**

To evaluate the accuracy of heart rate monitoring by a personal fitness tracker (PFT) among hospital inpatients.

**Methods:**

We conducted a prospective observational study of 50 stable patients in the intensive care unit who each completed 24 hours of heart rate monitoring using a wrist-worn PFT. Accuracy of heart rate recordings was compared with gold standard measurements derived from continuous electrocardiographic (cECG) monitoring. The accuracy of heart rates measured by pulse oximetry (Spo_2_.R) was also measured as a positive control.

**Results:**

On a per-patient basis, PFT-derived heart rate values were slightly lower than those derived from cECG monitoring (average bias of −1.14 beats per minute [bpm], with limits of agreement of 24 bpm). By comparison, Spo_2_.R recordings produced more accurate values (average bias of +0.15 bpm, limits of agreement of 13 bpm, *P*<.001 as compared with PFT). Personal fitness tracker device performance was significantly better in patients in sinus rhythm than in those who were not (average bias −0.99 bpm vs −5.02 bpm, *P*=.02).

**Conclusions:**

Personal fitness tracker–derived heart rates were slightly lower than those derived from cECG monitoring in real-world testing and not as accurate as Spo_2_.R-derived heart rates. Performance was worse among patients who were not in sinus rhythm. Further clinical evaluation is indicated to see if PFTs can augment early warning systems in hospitals.

**Trial Registration:**

ClinicalTrials.gov NCT02527408; https://clinicaltrials.gov/ct2/show/NCT02527408 (Archived by WebCite at  http://www.webcitation.org/6kOFez3on)

## Introduction

Over the last 5 years, consumer interest in self-monitoring and personal health tracking has grown considerably [1-4]. What began as a small movement among self-described “Quantified Self” enthusiasts has grown into an industry that is worth an estimated US $9 billion worldwide and is projected to grow to US $30 billion by 2018 [5]. This growth is largely driven by consumer interest in recording and reviewing high-frequency data about activity levels and general health in order to modify personal habits and promote healthy lifestyles. Data are generated by so-called wearables, small electronic devices that contain sensors and computing capabilities, which can be worn on a part of the body or integrated into clothing [5].

There has been growing enthusiasm for the potential use of wearable devices to improve health care delivery [4,6]. A number of different wearable sensors have been developed, which generate data that could potentially be useful in health care [7-9]. For instance, accelerometers have been incorporated into wearable devices to track physical activity such as walking, running, or climbing stairs and have also been used to evaluate sleep quality [10]. Wearable devices have seen only limited deployment in patient care settings, but their presence in clinics and hospitals is expected to grow significantly in the coming years [5].

Current clinical uses for wearable devices are mostly limited to outpatient and ambulatory settings, with a focus on the management of chronic diseases [11-13]. Applications include long-term ambulatory electrocardiogram (ECG) monitoring, optimizing pulmonary rehabilitation in patients with chronic obstructive pulmonary disease, and monitoring motor function in stroke patients as well as patients with Parkinson disease [11].

There is ample opportunity to leverage the sensing capabilities of wearable devices in the inpatient setting as well. Many newer wearable devices use photoplethysmography (PPG) to record heart rate by measuring differential reflection of light from the skin, based on the pulsatility of superficial blood vessels [14]. Heart rate sensing devices may be useful in extending the reach of vital signs monitoring in hospitals, which is typically limited by constraints on human resources. These signs, including heart rate, are monitored only a few times each day in ward settings. More frequent monitoring of heart rate stands to improve timely identification of deteriorating health of patients, increasing the chances that costly admission to the intensive care unit (ICU) can be avoided [15-19]. Heart rate surveillance also has the potential to identify patients with poorly controlled pain, to recognize incident arrhythmias, to detect sympathomimetic states such as alcohol withdrawal, and to generate more granular datasets for clinical research. A low-cost system capable of hospital-wide heart rate monitoring would therefore be valued in a time when health care expenditures are under increasing scrutiny [20].

The ability of wearable PPG sensors to reliably measure heart rate in the outpatient population has been demonstrated in at least one study [12]; however, their accuracy in hospital inpatients has not been firmly established. Equally uncertain are the accuracy and reliability of heart rate data derived from less costly wearables, such as commercially available personal fitness trackers (PFTs).

In order to address these and other questions, we examined the accuracy of heart rate measurements derived from PFTs. We focused on patients in the ICU as this cohort is closely monitored using continuous ECG (cECG) monitoring, which provides a gold standard measurement of heart rate. Because the degree of agreement that would be sufficient for clinical applications is not well defined, we also examined the agreement between cECG-derived heart rate measurements and a more widely accepted method of heart rate measurement, namely, pulse oximetry (Spo_2_) monitoring.

## Methods

### Study Setting and Patients

We used the Fitbit Charge HR (Fitbit, San Francisco, CA) PFT to monitor heart rate in 50 patients admitted to the ICU at Kingston General Hospital (KGH), a tertiary academic medical center in Ontario. The 33-bed ICU at KGH is a mixed medical, surgical, trauma, and neurosciences unit. The PFT device studied is a wrist-worn device resembling a watch, which uses PPG to detect periodic changes in blood flow beneath the sensor, thereby deriving heart rate measurements. Heart rate values are recorded every 5 minutes. The Fitbit Charge HR is a commercially available PFT and is not currently regulated by the US Food and Drug Administration.

In order to study a cohort of patients that would best resemble hospital ward patients, we included only stable patients who were not receiving mechanical ventilation, continuous analgesia, or sedation. To reduce the risk of transmitting nosocomial infections, we excluded patients under contact precautions for methicillin-resistant *Staphylococcus aureus* and *Clostridium difficile* infections. We further excluded patients with the potential for vascular compromise of the arm on which the device was to be placed, including those with deep venous thrombosis of the upper extremity, peripherally inserted central catheters, radial arterial lines, dialysis fistulas, and severe upper extremity trauma or fracture. Patients were monitored only once for a total duration of 24 hours.

### Data Capture

The study used 6 separate PFTs (3 size large, 3 size extra-large), each of which was assigned a unique email address and log-in credentials for the Fitbit website. An automated R script was used to download and process PFT data from the Fitbit website. Heart rate data are recorded by the PFT every 5 minutes. To provide a gold standard measurement of heart rate, we recovered data from the ICU bedside monitors using specialized software (BedMasterEX, Excel Medical, Jupiter, FL). Data included heart rate values, as well as heart rate data derived from continuous Spo_2_ monitoring (Spo_2_.R), both recorded every minute. These data were acquired as XML files and processed using an automated Python script to derive minute-level heart rate data. We synchronized bedside monitor data and PFT data using a correction factor that accounted for the difference between each device’s internal clock.

### Statistical Analysis

We analyzed heart rate data in aggregate across all patients, as well as on a per-patient basis. We determined the difference between cECG and PFT readings measured simultaneously, the median of differences over a 24-hour period, the interquartile range (IQR) of differences, and the Pearson correlation coefficient between cECG and PFT measurements. We used a Wilcoxon signed rank test to determine if the distribution of cECG-derived heart rates differed from that of the PFT-derived heart rates. Finally, we used Bland-Altman analysis to measure the agreement between the PFT and cECG methods of heart rate monitoring, as well as the bias of the PFT relative to cECG.

We calculated all the above-mentioned metrics for cECG-Spo_2_.R pairs in order to compare the accuracy of PFT measurements with that of a well-established and widely used alternative for heart rate measurement. On the basis of the mechanism of sensing used by the PFTs, we hypothesized that accuracy would differ in patients not in sinus rhythm and conducted a subgroup analysis to test this effect. Rhythm status was based on examination of cECG recording both at the time of device application and at the time of device removal, with patients designated as being in sinus rhythm only if this was present at both time points. To assess the potential for degradation in PFT performance over time, we compared the accuracy in the first 20 patients with that of the last 20 patients.

The study was approved by the Health Sciences Research Ethics Board of Queen’s University (DMED-1818-15) and is registered with ClinicalTrials.gov (NCT02527408). Patients or their substitute decision makers provided informed consent. All study data were deidentified. The study did not receive funding from the device manufacturer or from any other source. All statistical analyses were done using R (v 3.2.2).

## Results

### Patients

Between August 2015 and January 2016, we enrolled a convenience sample of 50 patients meeting our enrollment criteria. Patients were admitted with a variety of medical and surgical conditions and were of low clinical acuity at the time of monitoring (Table 1).

**Table 1:** Characteristics of patients included in the study (N=50)

Characteristics		Values
Mean heart rate, beats per minute		88.3
Mean age, years		64
**Sex, n (%)**		
	Male	26 (52)
	Female	24 (48)
**Admission diagnosis, n (%)**		
	Respiratory	12 (24)
	Sepsis	7 (14)
	Surgical	7 (14)
	Neurologic	11 (22)
	Trauma	3 (6)
	Cardiovascular	6 (12)
	Medical	4 (8)
**Sinus rhythm, n (%)**		
	At start of monitoring	43 (86)
	At end of monitoring	42 (84)
**Personal fitness tracker size used, n (%)**		
	Large	23 (46)
	Extra large	27 (54)

### Data Acquisition

The PFT device was removed prematurely in 2 cases; in one case a patient was discharged from the ICU early, and in another case a patient developed a diffuse drug rash. In 4 cases cardiorespiratory monitoring was discontinued early, resulting in incomplete comparison data. Personal fitness tracker devices in these cases continued to collect data for the full 24-hour period. Excluding the 2 patients whose devices were removed early, PFTs showed a high degree of data capture (mean 98% of eligible data).

### Heart Rate Accuracy

**Figure 1:**
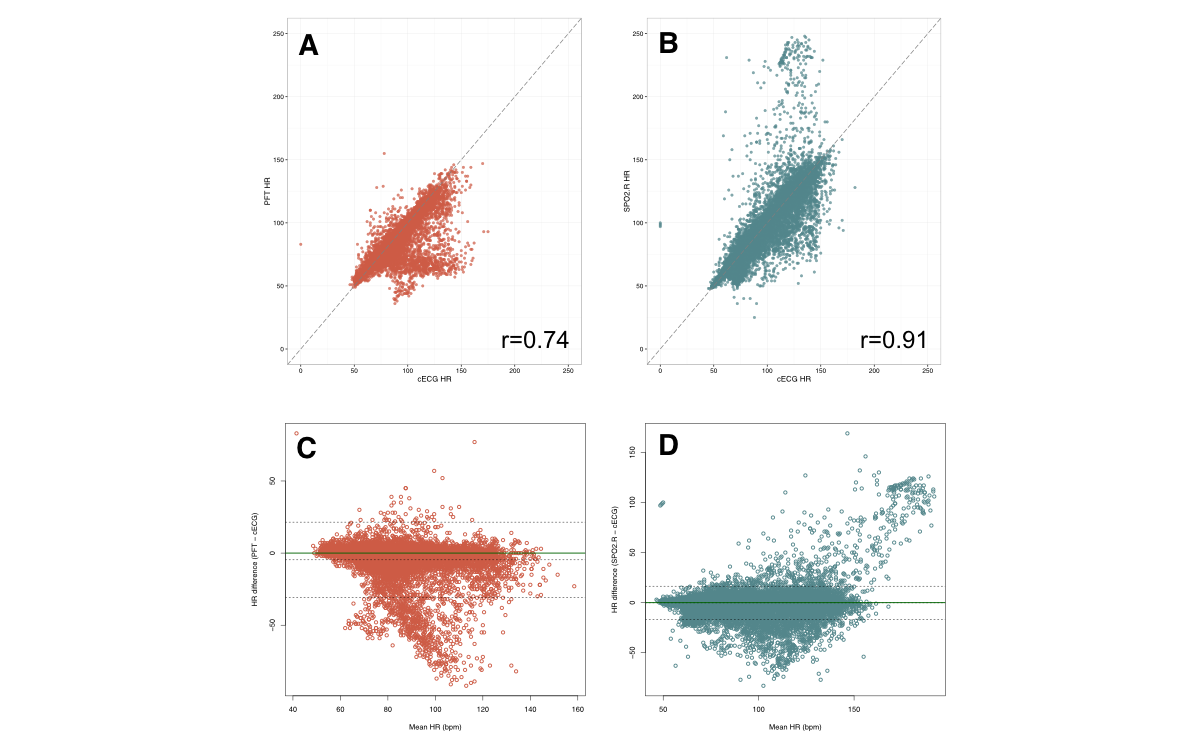
Results of the pooled analysis comparing continuous electrocardiogram (cECG)-derived heart rates and personal fitness tracker (PFT)-derived heart rates (in red), as well as cECG-derived heart rates and pulse oximetry heart rates (SpO_2_.R, in blue). A and B, Scatterplots showing simultaneous heart rate measurements from cECG (x-axis) compared with alternative methods (y-axis). C and D, Bland-Altman plots for heart rate measured by PFT and SpO_2_.R compared with cECG. Mean heart rate is shown on the x-axis, with the difference between heart rates shown on the y-axis. The solid horizontal line represents a difference between measurements of 0, while the dashed lines represent the observed mean difference (bias) and limits of agreement. HR: heart rate; bpm: beats per minute.

We analyzed a total of 12,358 cECG-PFT heart rate pairs and 56,385 cECG-Spo_2_.R heart rate pairs. Most of the 24-hour heart rate recordings conformed to a skewed or bimodal distribution (Multimedia Appendix 1). In the pooled analysis (Figure 1 and Multimedia Appendix 2), the median difference between PFT-derived heart rates and cECG-derived heart rates was 1 beat per minute (bpm), with 73% of readings within 5 bpm of the cECG value. The correlation with cECG heart rate values was .74, and the distribution of PFT-derived heart rate values was significantly different from that of the cECG values (*P*<.001). By comparison, Spo_2_.R-derived heart rates more closely approximated cECG, with a median difference of 0 bpm, correlation coefficient of .91, and 89% of readings within 5 bpm of the cECG value. The Spo_2_.R and cECG heart rate distributions were similar (*P*=.18). Visual inspection of the Bland-Altman plots revealed a tendency for the PFT to underestimate heart rate values in the range of approximately 75 to 120 bpm (Figure 1, part C). There was greater bias with the PFT method compared with the Spo_2_.R method (−4.7 bpm, 95% CI −4.91 to −4.44 bpm, vs −0.2 bpm, 95% CI −0.30 to −0.16 bpm). The limits of agreement were wider with the PFT method compared with the Spo_2_.R method: −31 (95% CI −31.22 to −30.40 bpm) to 21 bpm (95% CI 21.06-21.87 bpm) versus −17 (95% CI −17.01 to 16.77 bpm) to 16 bpm (95% CI 16.31-16.55 bpm; see Multimedia Appendix 3).

### Per-Patient Analysis

**Figure 2:**
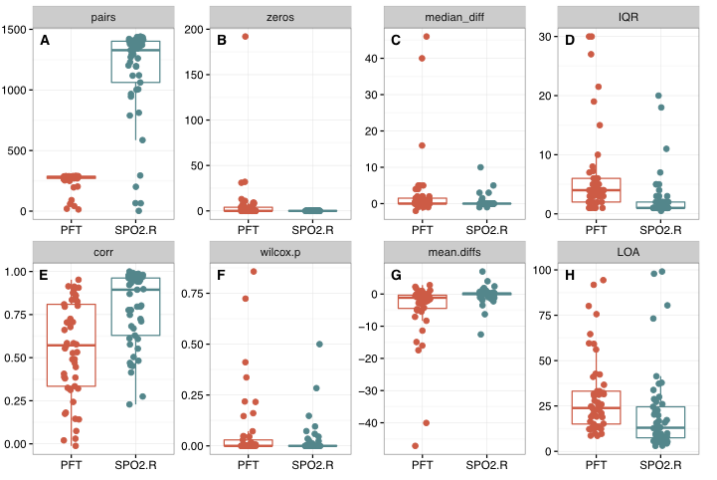
Summary of the per-patient analysis of the differences between personal fitness tracker (PFT, shown in red) and pulse oximetry heart rate values (SpO_2_.R, shown in blue) as compared with continuous electrocardiogram (cECG)–derived heart rate. A, Number of heart rate pairs analyzed. B, Number of zero measurements recorded. C, Median difference between the device-derived heart rate and cECG-derived heart rate. D, Interquartile range (IQR) of the differences. E, Pearson correlation coefficient (PFT vs cECG and SpO_2_.R vs cECG). F, P values indicating the likelihood that the distribution of heart rate values derived from the other devices differed from that derived from cECG (Wilcoxon signed rank test). G, Mean difference between device-derived heart rate and cECG-derived heart rate from Bland-Altman analysis. H, Limits of agreement between device-derived heart rate and cECG-derived heart rate from Bland-Altman analysis. For each boxplot, individual patients are represented by an individual point. All comparisons showed statistically significant differences by Wilcoxon rank sum test (*P*<.01), with the exception of the comparison of P values (*P*=.57).

Scatterplots for individual patients are provided in Multimedia Appendix 4. Summary statistics are presented in Table 2 and Figure 2. Although the median heart rate difference was 0 for both the PFT device and Spo_2_.R readings, when compared with cECG, there was a statistically significant difference between these 2 groups (*P*=.003). On average, PFT recordings yielded a higher IQR, lower Pearson correlation coefficient, larger bias, and wider limits of agreement than Spo_2_.R recordings (*P*<.001 for all comparisons).

**Table 2:** Results of the per-patient analysis of heart rate recording accuracy.

Variable	PFT^a^	Spo_2_.R^b^	*P* value
Pairs	281	1328	<.001
Zeros (median)	0	0	<.001
Median difference (bpm^c^)	0	0	.003
Interquartile range (bpm)	4	1	<.001
Correlation coefficient	.57	.89	<.001
Wilcoxon ­ *P* value	1.52E−06	5.06E−11	.57^d^	
Bias (bpm)	−1.14	0.15	<.001
Limits of agreement (bpm)	23.88	13.00	<.001

^a^PFT: personal fitness tracker.

^b^Spo_2_.R: pulse oximetry heart rate.

^c^bpm: beats per minute.

^d^comparing the number of recordings with Wilcoxon ­ *P* value < .05.

**Figure 3:**
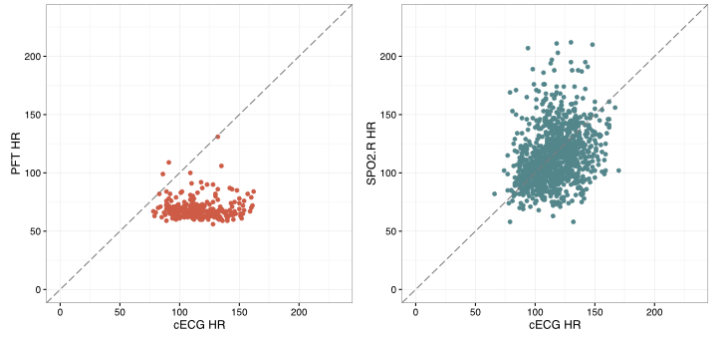
Scatterplots showing simultaneous heart rate (HR) measurements derived from continuous electrocardiogram (cECG; x-axis) compared with heart rate from personal fitness tracker (PFT, left) and pulse oximetry (SpO_2_.R, right) in a patient with atrial fibrillation. In the absence of normal sinus rhythm, the PFT consistently underestimated heart rate.

**Figure 4:**
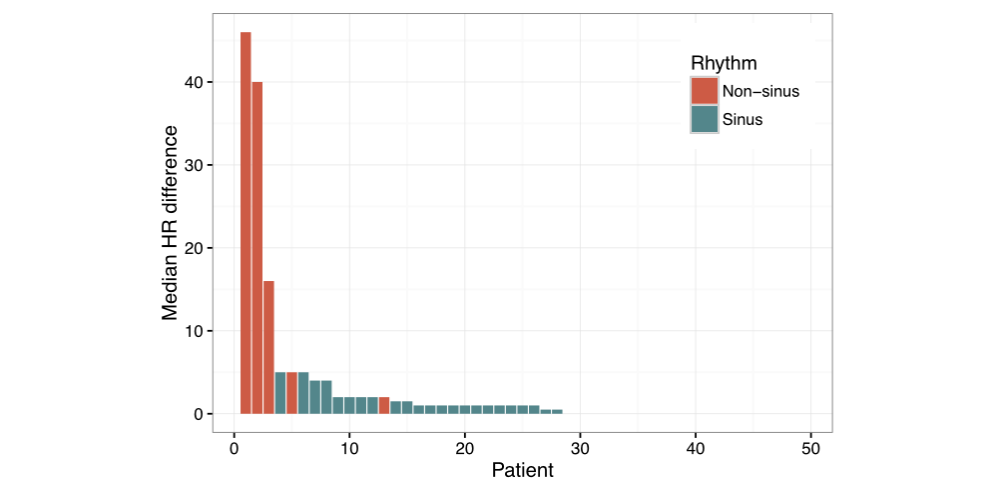
Patient recordings arranged according to median difference between personal fitness tracker–derived heart rate and heart rate from continuous electrocardiogram. The least accurate recordings were from patients who were not in sinus rhythm. HR: heart rate.

Our subgroup analyses compared 8 patients who were not in sinus rhythm with 40 patients in sinus rhythm. Median heart rate difference, IQR, Pearson correlation coefficient, bias, and limits of agreement were all significantly worse in patients with rhythms other than sinus (*P*<.05 for all comparisons, Table 3). An example of poor PFT performance is shown in Figure 3. Of the 5 recordings showing the worst PFT performance, 4 were from patients not in sinus rhythm (Figure 4). There was no difference in the correlation between PFT heart rates and cECG-derived heart rates between the first 20 patients enrolled and the last 20 patients (mean Pearson correlation coefficient .51 vs .46, *P*=.61). Individual PFT devices were used between 5 and 13 times (mean 9 times).

**Table 3:** Heart rate measurement accuracy in patients in sinus rhythm compared with those not in sinus rhythm.

Measurement	Sinus rhythm (n=40)	Nonsinus rhythm (n=8)	*P* value
Median difference (bpm^a^)	0	3.5	.04
Interquartile range (bpm)	4	8.6	.01
Correlation coefficient	.58	.23	<.001
Bias (bpm)	−0.99	−5.02	.02
Limits of agreement (bpm)	22.9	46.4	.049
			

^a^bpm: beats per minute.

## Discussion

### Wearables in Health Care

Although the use of wearables in health care has garnered considerable attention in recent years, few objective studies exist resulting in a substantial dearth of clinical evidence regarding their use. A recent PubMed search of the term “wearable technology” revealed nearly 1000 articles published in the last 5 years, only 3% of which were clinical trials [21]. None of these included acutely ill patients. Despite the absence of evidence regarding their accuracy, data from PFTs have been used in acute care settings, including in one recently published case of a patient presenting to an emergency department who received electrical cardioversion for stable atrial fibrillation [22]. Data derived under real-world conditions from clinical settings are needed to better define the role of wearable devices in general, and commercially available fitness trackers in particular, in the delivery of acute care medicine. We conducted an observational study of heart rate monitoring accuracy of a commercially available PFT in order to provide objective evidence regarding the accuracy of its heart rate monitoring capabilities among hospitalized patients.

### Principal Findings

We found that, overall, Fitbit PFT-derived heart rate measurements were less accurate and consistent than heart rate values recorded by continuous pulse oximetry. There was, however, considerable between-patient heterogeneity, with PFT heart rate values proving highly accurate in some cases and less so in others. With heart rate values analyzed on a per-patient basis, the differences between the PFT and pulse oximetry methods were less pronounced. The accuracy of PFT-based heart rate monitoring was poor among patients not in sinus rhythm.

Our results show that, on average, the PFT devices tested tended to underestimate heart rate values slightly, particularly with heart rate values in the range of 75 to 120 bpm. The clinical implications of this degree of bias are uncertain and likely depend on the intended purpose of the monitoring. A difference of the magnitude observed might be acceptable for detecting acute clinical deterioration, which is often accompanied by marked changes in heart rate, but may not be adequate for identifying more subtle physiological derangements.

Wrist-worn heart rate sensing devices have the potential to enhance inpatient safety by identifying episodes of clinical deterioration faster than current nurse-driven vital signs monitoring practices allow. With only a small minority of hospitalized patients receiving cECG monitoring in intensive care settings, most have heart rate measurements taken only 2 to 3 times in a 24-hour period. Early warning systems (EWSs) have been shown to accurately predict cardiac arrest and hospital mortality, with some studies suggesting a reduction in these events following EWS implementation [15]. Heart rate is a common variable factored into most EWS algorithms [15,17]. Derangements in heart rate in general, and tachycardia in particular, have consistently been shown to predict impending clinical deterioration [15-19]. Early warning system variants can be complicated and difficult to use on a practical level [15,23-25]. Commercially available PFTs suggest a potential solution to address shortfalls by supplementing the monitoring of ward patients with frequent heart rate measurements generated automatically.

There are a number of potential advantages to augmenting hospital monitoring practices using wrist-worn PPG-based heart rate sensors such as the one we studied. A cost advantage may be achievable given that the device we tested retails for approximately US $170 and that we were able to reuse devices on average 9 times without seeing a decrement in performance. By comparison, conventional heart rate monitoring on inpatient wards (ie, telemetry) has been estimated to incur expenses of just under US $40 per patient per day in direct costs and as much as an additional US $170 in opportunity costs [26]. Although PFTs do not measure any additional vital signs, they do record movement data that can be used to monitor physical rehabilitation [11]. Personal fitness trackers could therefore provide benefit through the continuum of an illness episode, by providing enhanced heart rate monitoring during the acute phases, accurate tracking of mobility during convalescence, and ongoing feedback to both patient and clinician following discharge.

The use of wrist-worn devices for heart rate monitoring in hospitals also has potential disadvantages. Consumer-grade PFTs do not provide information regarding respiratory rate or blood pressure, both of which have been shown to add value in EWS [17]. Wrist-worn PPG devices might also be susceptible to errors in heart rate measurement owing to the phenomenon of the pulse deficit, in which beat-to-beat variability in stroke volume alters the amplitude of the pulse. This can be seen in atrial fibrillation, as well as other physiological conditions of acute illness such as cardiac tamponade, status asthmaticus, and various shock states, and may explain the significant decrement in heart rate sensing accuracy seen in our subgroup of patients who were not in sinus rhythm. Heart rate reporting might therefore be less accurate in the patients for whom the recognition of clinical deterioration is most needed, namely, those developing hemodynamic instability. Whether a degradation of signal quality could be used to identify physiological decompensation remains unknown.

Our study has a number of strengths. We examined the use of PFTs in a sizeable cohort of hospitalized patients under real-world conditions. Devices were adjusted only once at the time of application and were not reassessed for the duration of the 24-hour recording period by either study personnel or clinical staff. We used high-frequency data captured from continuous bedside monitoring to provide an accurate gold standard assessment of heart rate and analyzed PFT performance on both a pooled and per-patient level.

### Limitations

One of the potential limitations of our study arises from the fact that the PFT-derived and cECG-derived heart rate values were obtained from different devices, with separate internal clocks. Although correction factors were used to synchronize the time stamps from the 2 heart rate sources, it is possible that in some cases the heart rate values that were treated as simultaneous were in fact separated by a short time interval. As the PFT device only recorded heart rate measurements every 5 minutes—an interval longer than the maximal device time discrepancy observed—the impact of any potential asynchrony was likely minimal.

Our study was conducted in the ICU, where cECG monitoring provides a gold standard comparator for heart rate. The extent to which our results can be generalized to hospitalized patients on the wards is therefore not certain; however, all patients enrolled were stable and were receiving ward-level care at the time of monitoring. Finally, our subgroup analysis included a relatively small number of patients not in sinus rhythm, thereby limiting the statistical power of the results.

Our study used one particular type of PFT, namely, the Fitbit Charge HR. Although many consumer-grade PFTs have similar intended functionality and use similar heart rate sensing technology, our results cannot necessarily be generalized to other wearable devices. Given that the different performance characteristics of various PFTs are not known, a study in which a mix of devices is used would be vulnerable to unwarranted mixing of effects or would require an increase in sample size proportional to the number of different devices tested.

### Comparison With Prior Work

Our study is the first to report on the accuracy of heart rate recordings from wearable devices among hospital inpatients. Previous work has focused on the technical and engineering aspects of PPG-based wearable heart rate sensors, as well as discussion of their potential uses in health care settings [7,8,11]. Studies regarding the accuracy of wearables have largely focused on activity tracking and have been done using healthy volunteers [27,28]. Our study differs from previous clinical evaluations of wearable devices [29,30] in its focus on heart rate monitoring rather than activity tracking, as well as its inclusion of inpatients rather than ambulatory patients.

### Conclusions

The health care sector is expected to drive a large proportion of sales of wearable devices in the coming years [5]. Optimal deployment and value from these devices will require clinical trials conducted under real-world conditions, to test the feasibility, accuracy, and costs associated with their use in health care settings. Our study suggests a potential role for PFTs in monitoring heart rate among inpatients; however, recording accuracy was not as high as with pulse oximetry and lagged substantially among patients not in sinus rhythm. Our findings suggest that future work should focus on identifying which patients are most suitable for PFT-derived heart rate monitoring, as well as software development to optimize recording accuracy in a wide range of illness states, including those associated with a pulse deficit. Although our results suggest that PFT-based heart rate monitoring may be highly accurate in some cases, prospective clinical trials are needed to evaluate their capacity to improve clinical outcomes as part of a larger strategy of enhanced hospital-based monitoring.

## Appendix

Multimedia Appendix 1 Frequency distributions for continuous electrocardiogram–derived heart rate (black), personal fitness tracker heart rate (red), and pulse oximetry heart rate (green) for each patient.

Multimedia Appendix 2 Table showing the results of the pooled analysis of heart rate comparisons.

Multimedia Appendix 3 Histogram showing the distribution of the obtained heart rate differences (x-axis) of both personal fitness tracker and pulse oximetry (SpO_2_) when compared with continuous electrocardiogram, in beats per minute.

Multimedia Appendix 4 Scatterplots (continuous electrocardiogram–derived heart rate vs device-derived heart rate) for each patient.

## Abbreviations

bpm: beats per minute
ECG: electrocardiogram
EWS: early warning system
cECG: continuous electrocardiogram
ICU: intensive care unit
IQR: interquartile range
KGH: Kingston General Hospital
PFT: personal fitness tracker
PPG: photoplethysmography
SpO_2_: pulse oximetry
SpO_2_.R: pulse oximetry heart rate
