# Supplementary material for: Accuracy of a Wrist-Worn Wearable Device for Monitoring Heart Rates in Hospital Inpatients: A Prospective Observational Study
Source: J Med Internet Res. 2016 Sep 20;18(9):e253. doi: 10.2196/jmir.6025 (PMC5050383; doi:10.2196/jmir.6025)
Supplement: Multimedia Appendix 1 [file jmir_v18i9e253_app1.pdf]

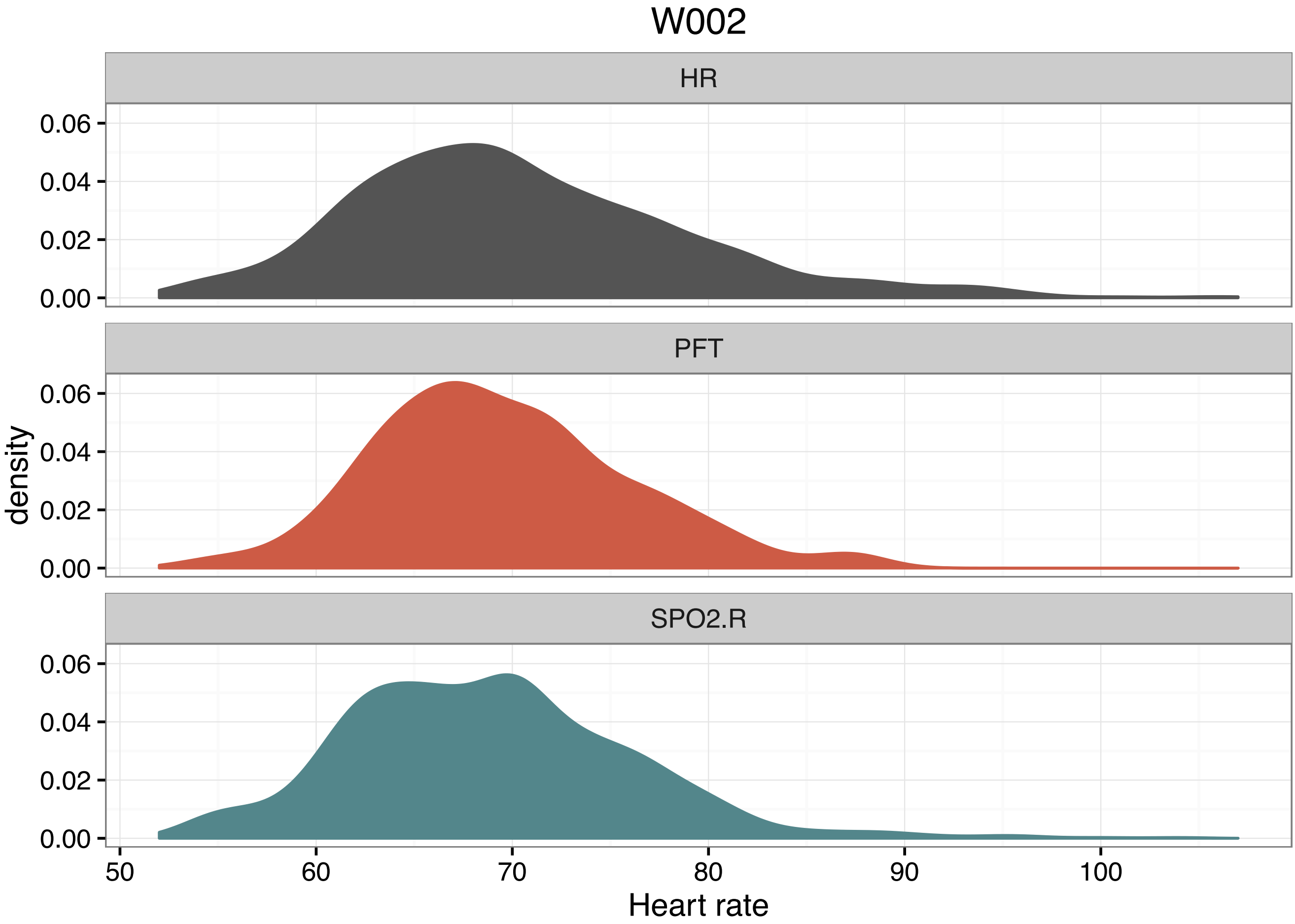
W002
HR
PFT
SPO2.R
density
0.06
0.04
0.02
0.00
50
60
70
80
90
100
Heart rate

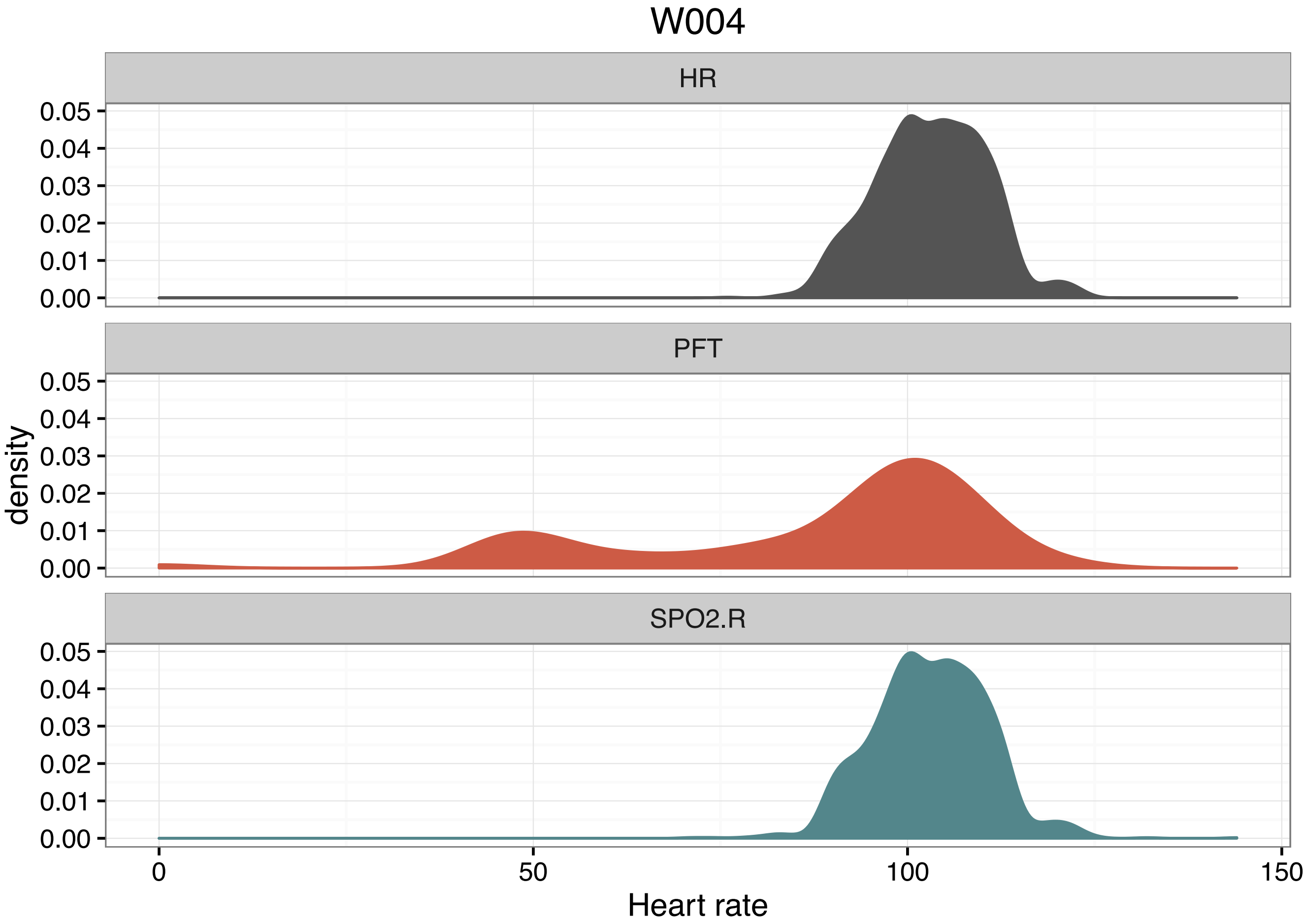

W004
HR
PFT
SPO2.R
density
0.00
0.01
0.02
0.03
0.04
0.05
0
50
100
150
Heart rate

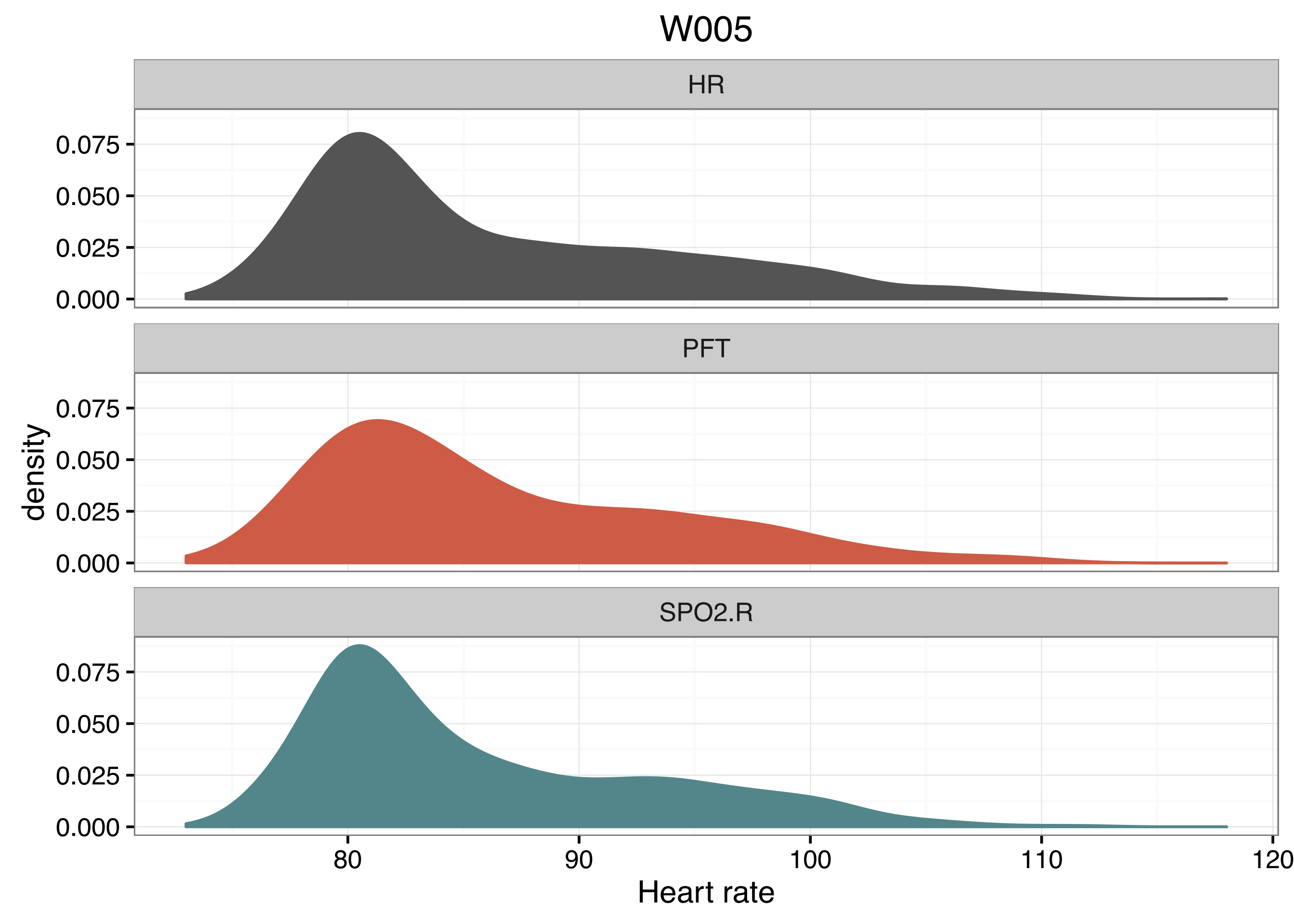
W005
HR
PFT
SPO2.R
density
0.075
0.050
0.025
0.000
80
90
100
110
120
Heart rate

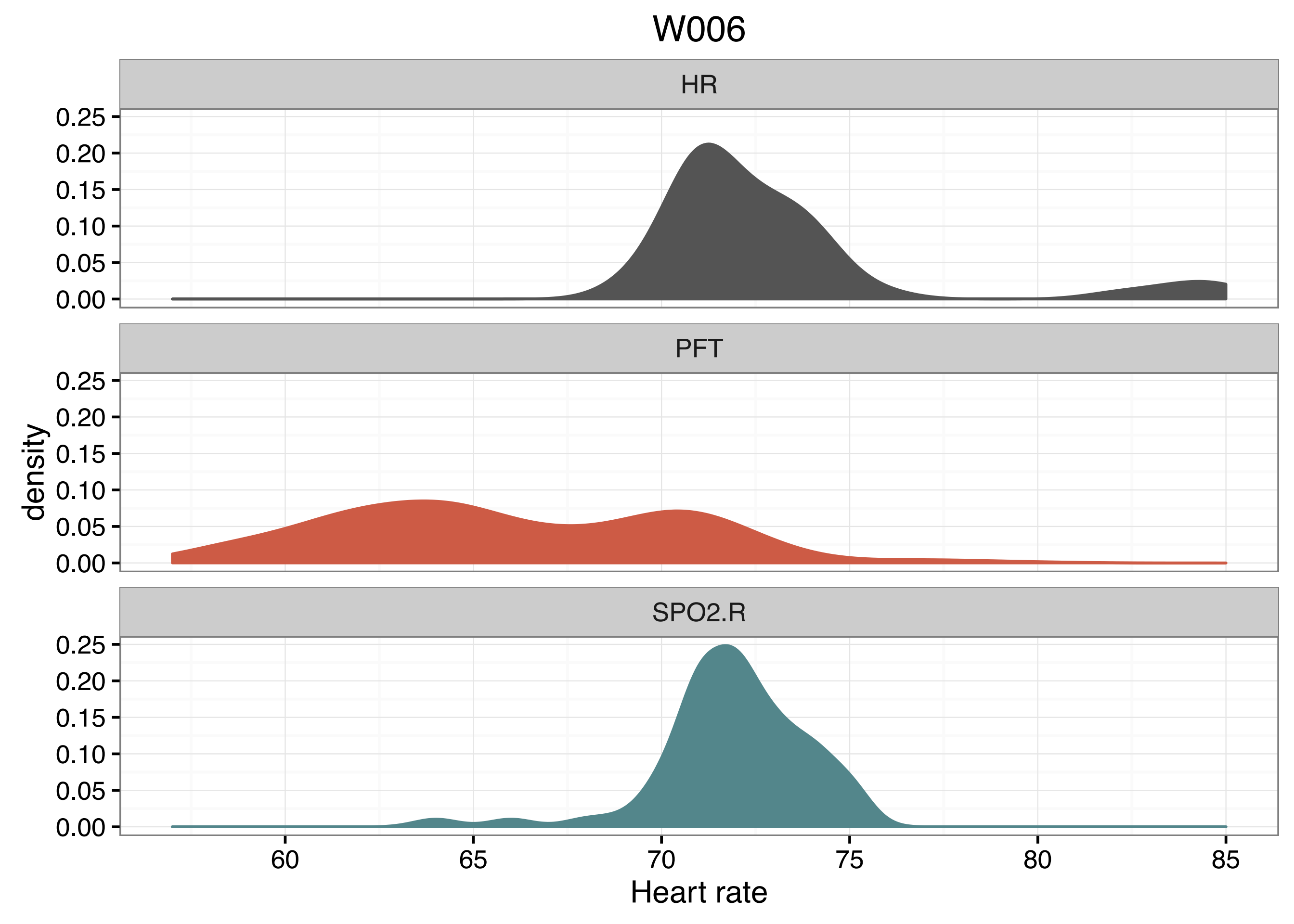
W006
HR
PFT
SPO2.R
density
0.00
0.05
0.10
0.15
0.20
0.25
60
65
70
75
80
85
Heart rate

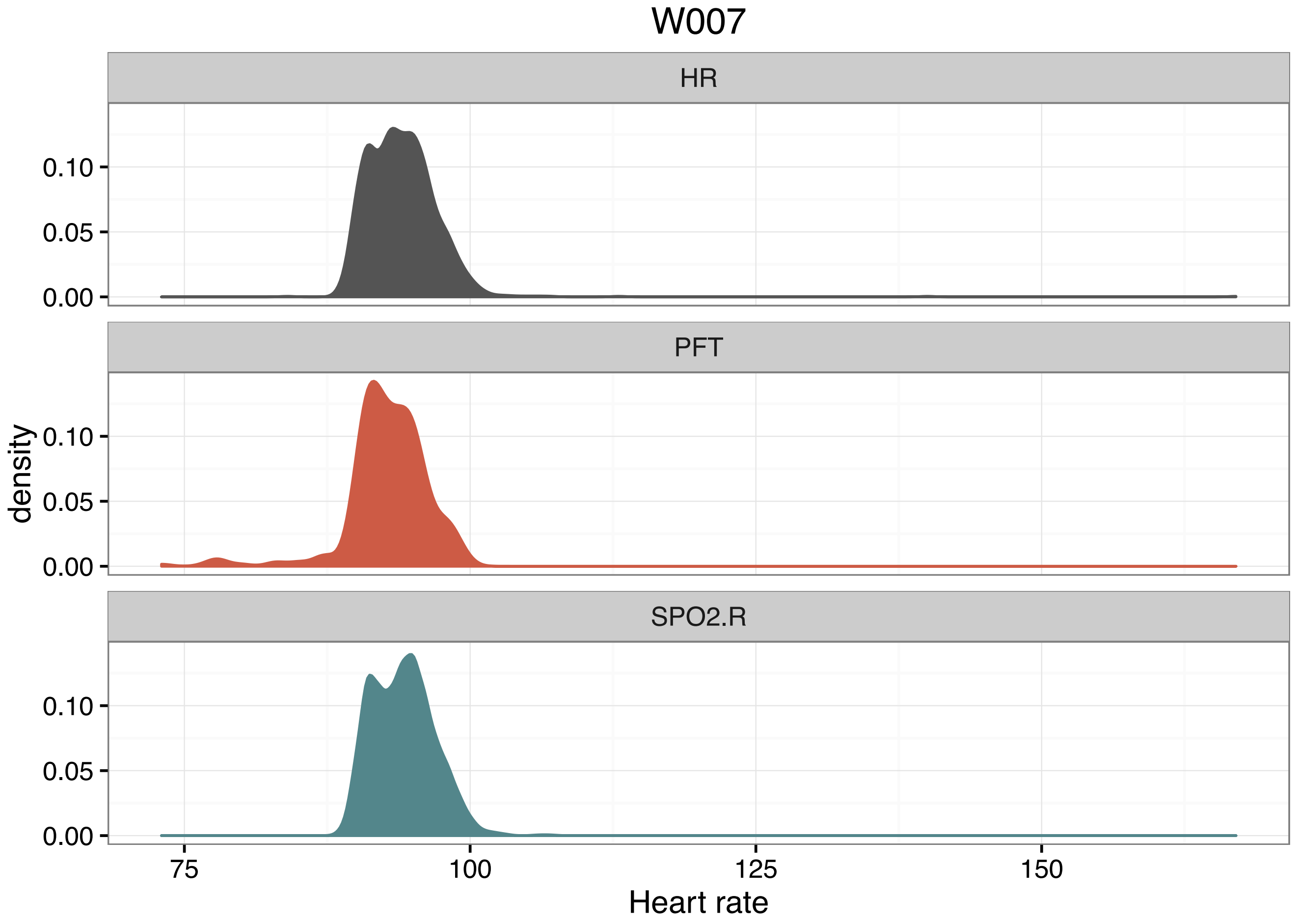

W007
HR
PFT
SPO2.R
density
0.10
0.05
0.00
75
100
125
150
Heart rate

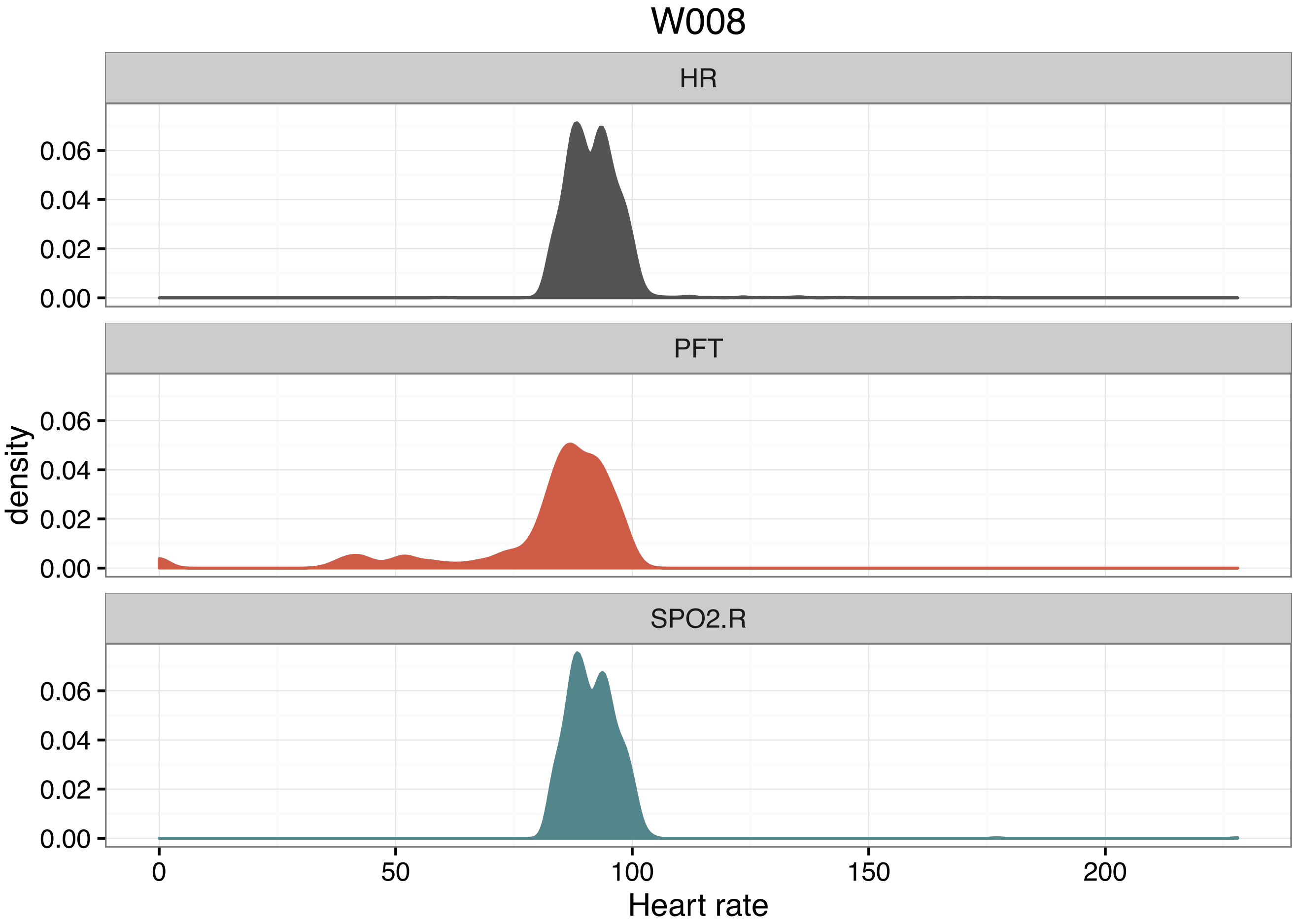
W008
HR
PFT
SPO2.R
density
0.06
0.04
0.02
0.00
0
50
100
150
200
Heart rate

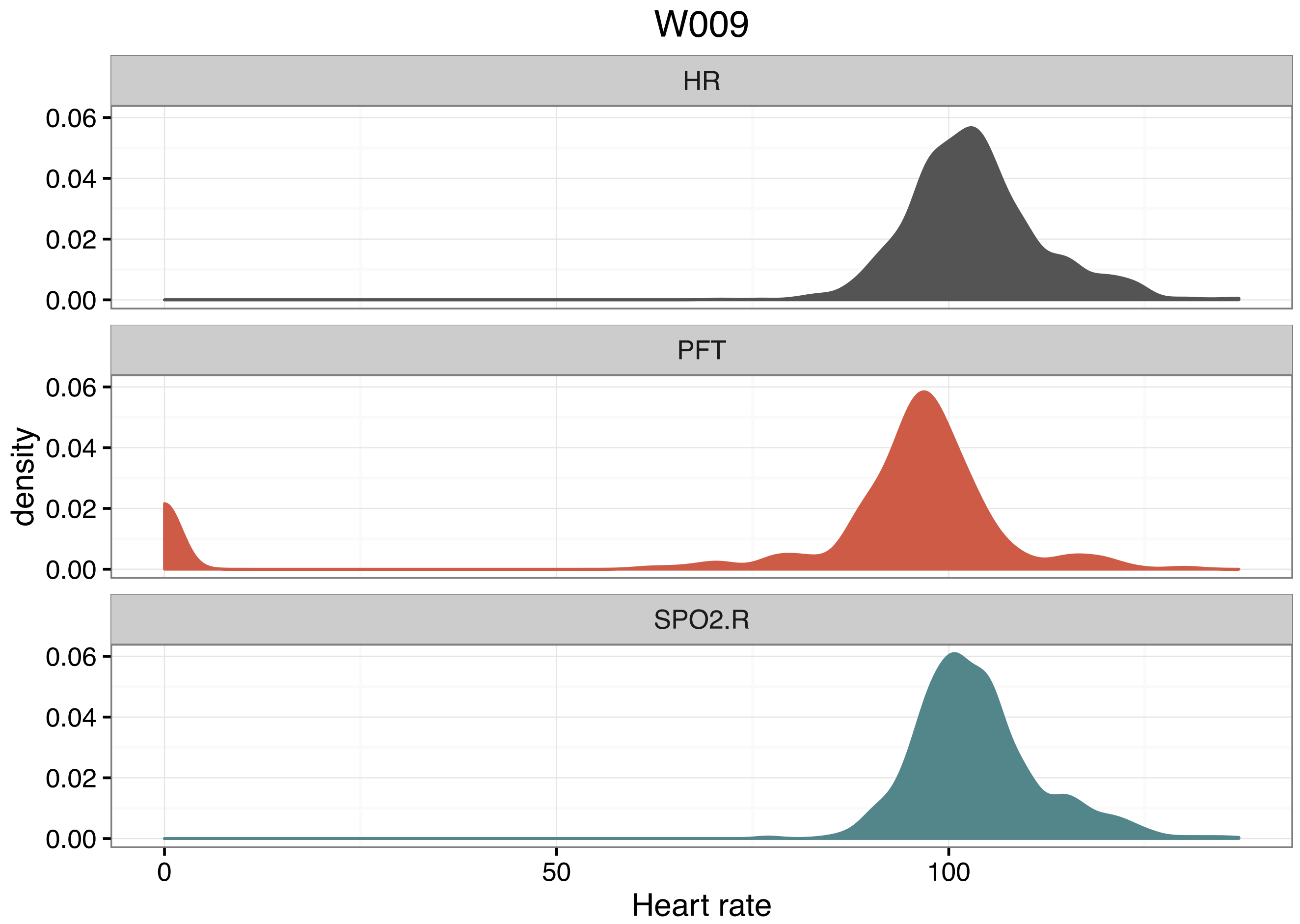
W009
HR
PFT
SPO2.R
density
0.06
0.04
0.02
0.00
0
50
100
Heart rate

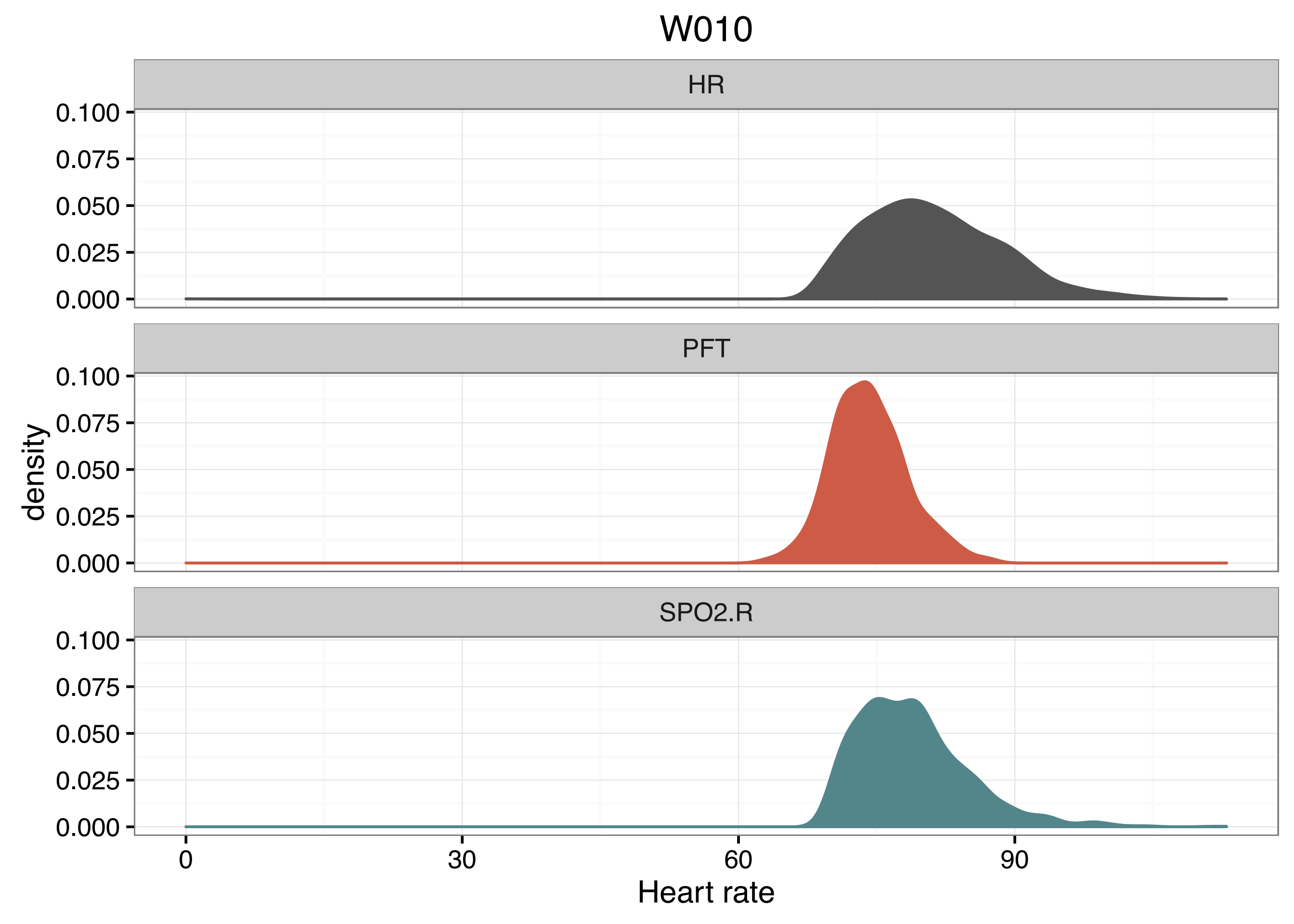
W010
HR
PFT
SPO2.R
density
0.100
0.075
0.050
0.025
0.000
0
30
60
90
Heart rate

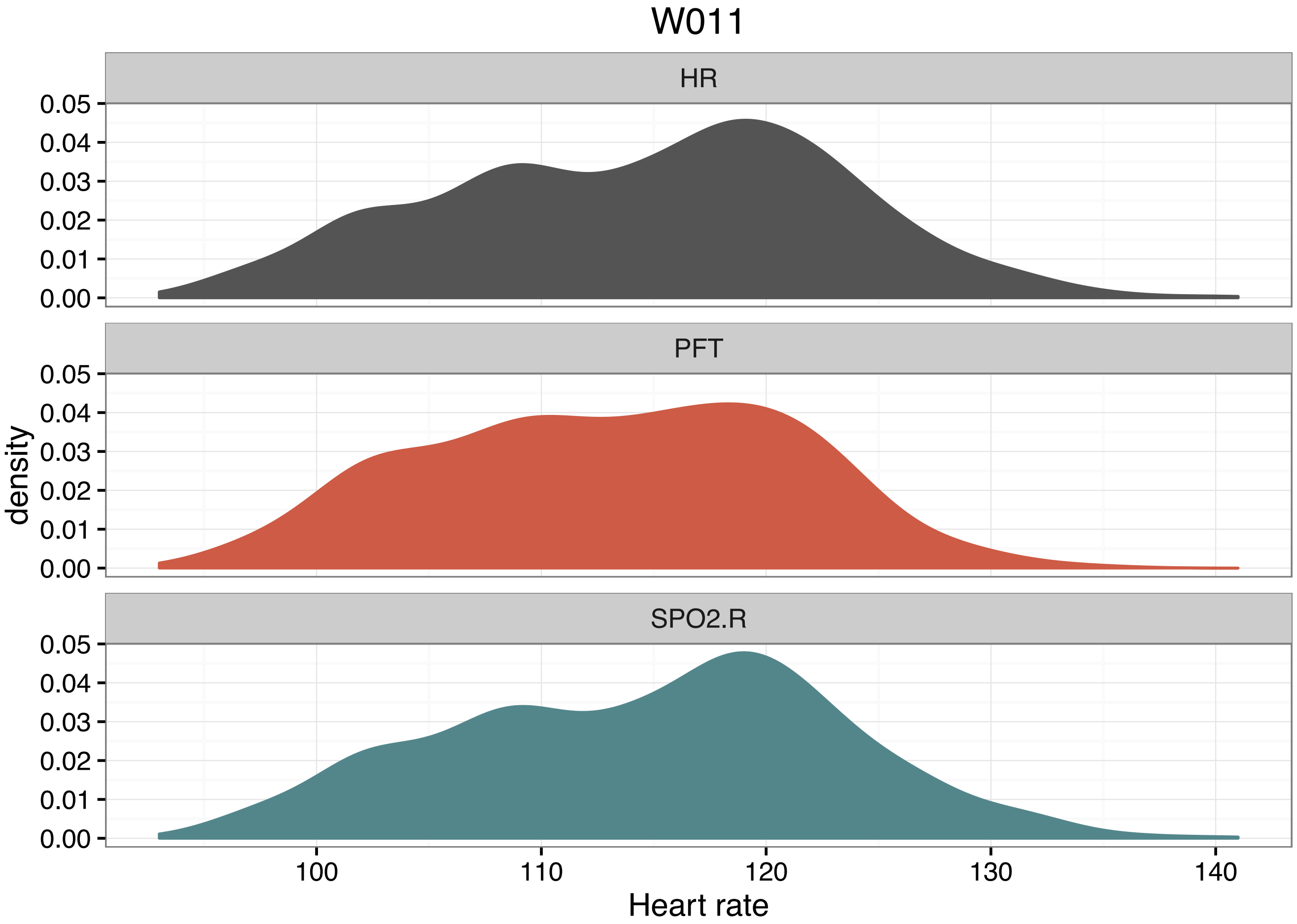

W011
HR
PFT
SPO2.R
density
0.05
0.04
0.03
0.02
0.01
0.00
100
110
120
130
140
Heart rate

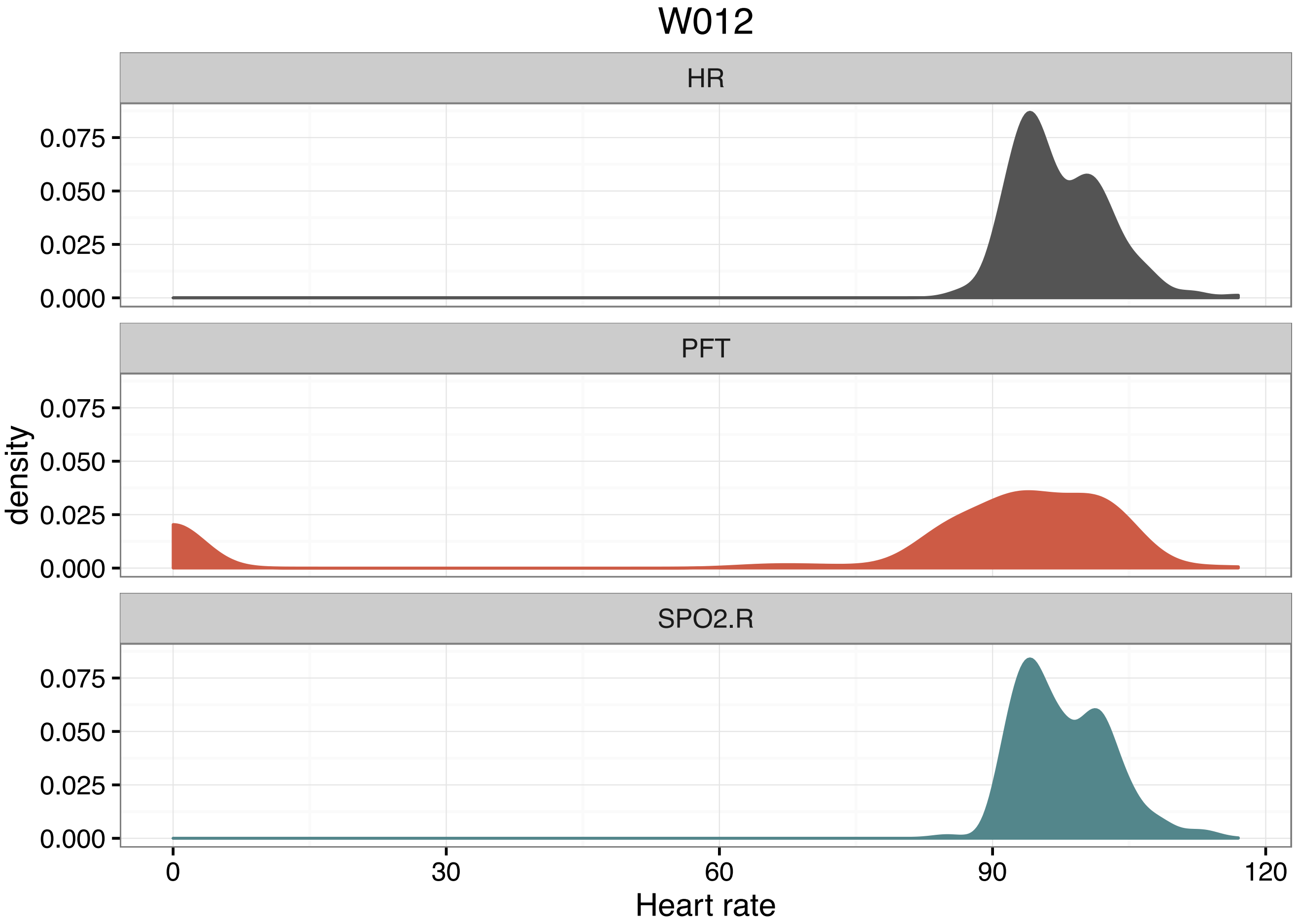
W012
HR
PFT
SPO2.R
0.075
0.050
0.025
0.000
density
0
30
60
90
120
Heart rate

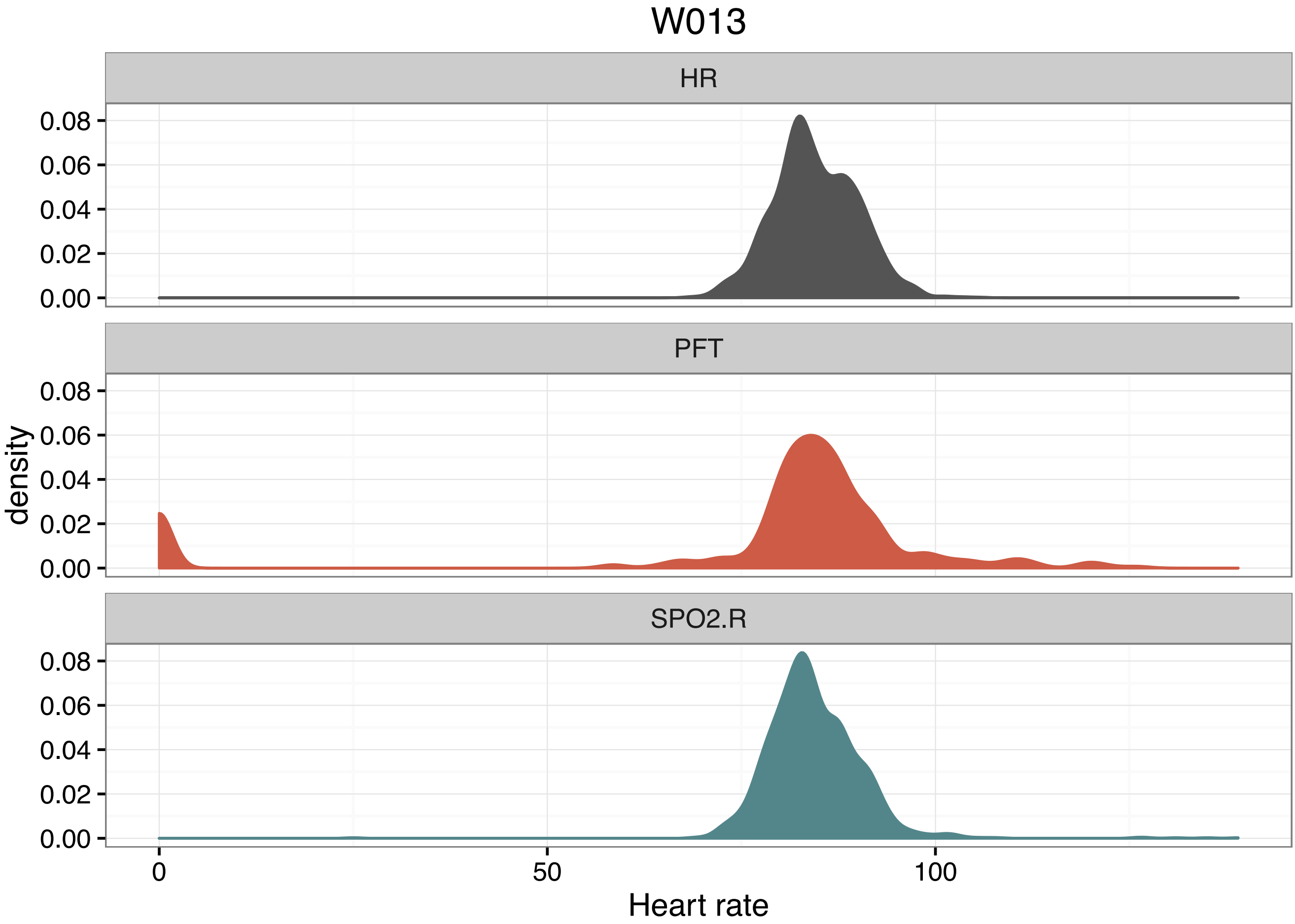
W013
HR
PFT
SPO2.R
density
0.08
0.06
0.04
0.02
0.00
0
50
100
Heart rate

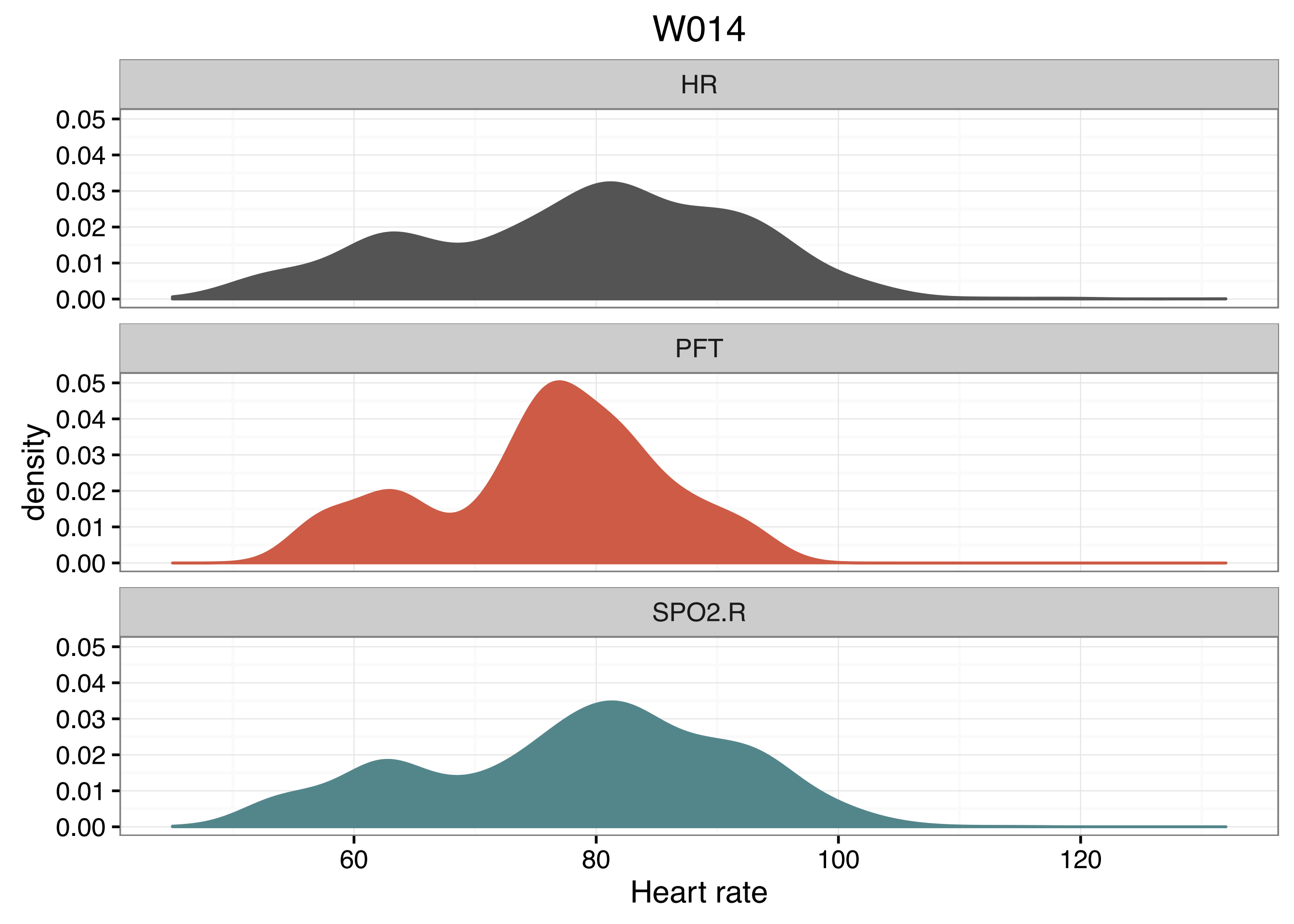

W014
HR
PFT
SPO2.R
density
0.05
0.04
0.03
0.02
0.01
0.00
60
80
100
120
Heart rate

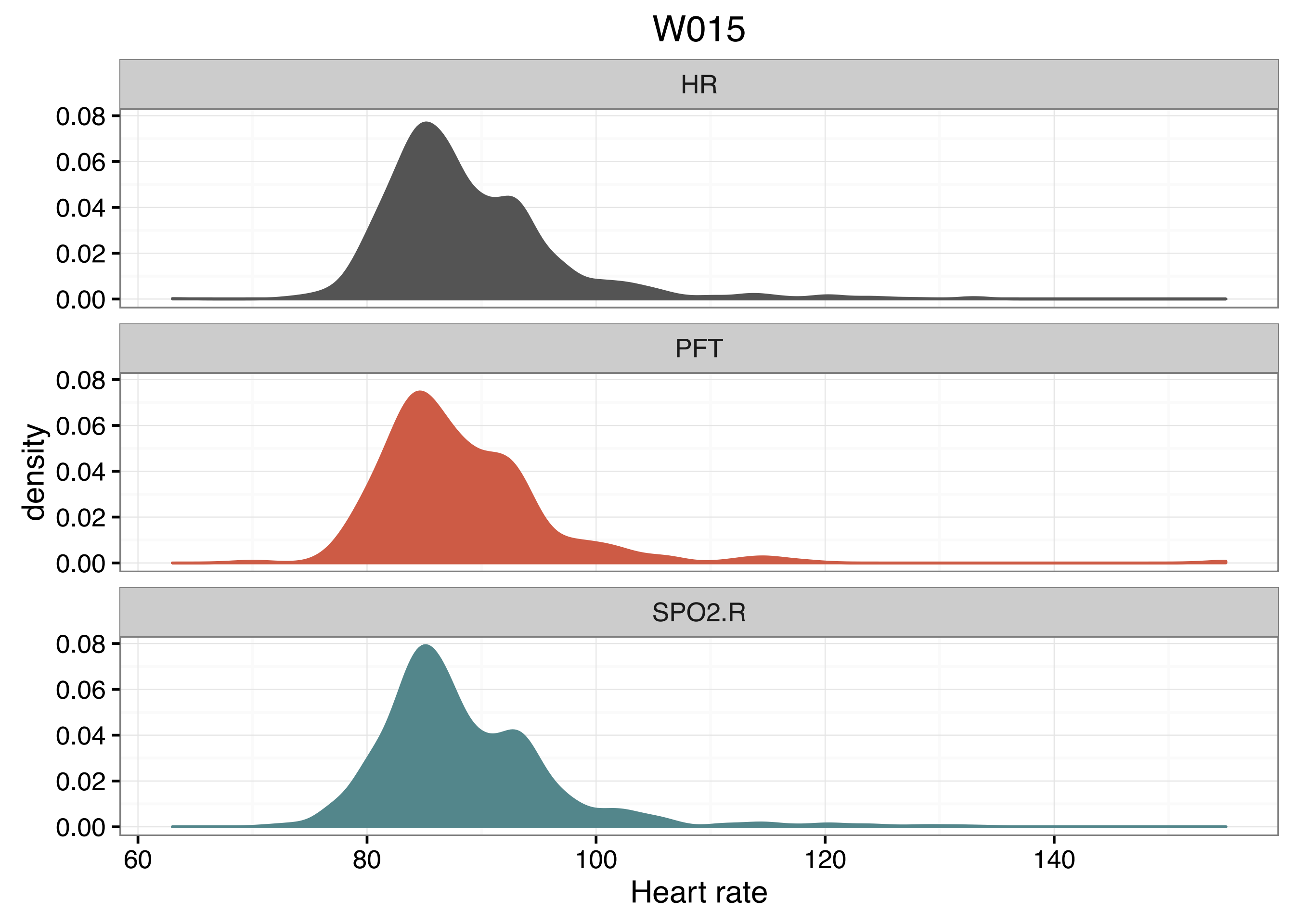
W015
HR
PFT
SPO2.R
density
0.08
0.06
0.04
0.02
0.00
60
80
100
120
140
Heart rate

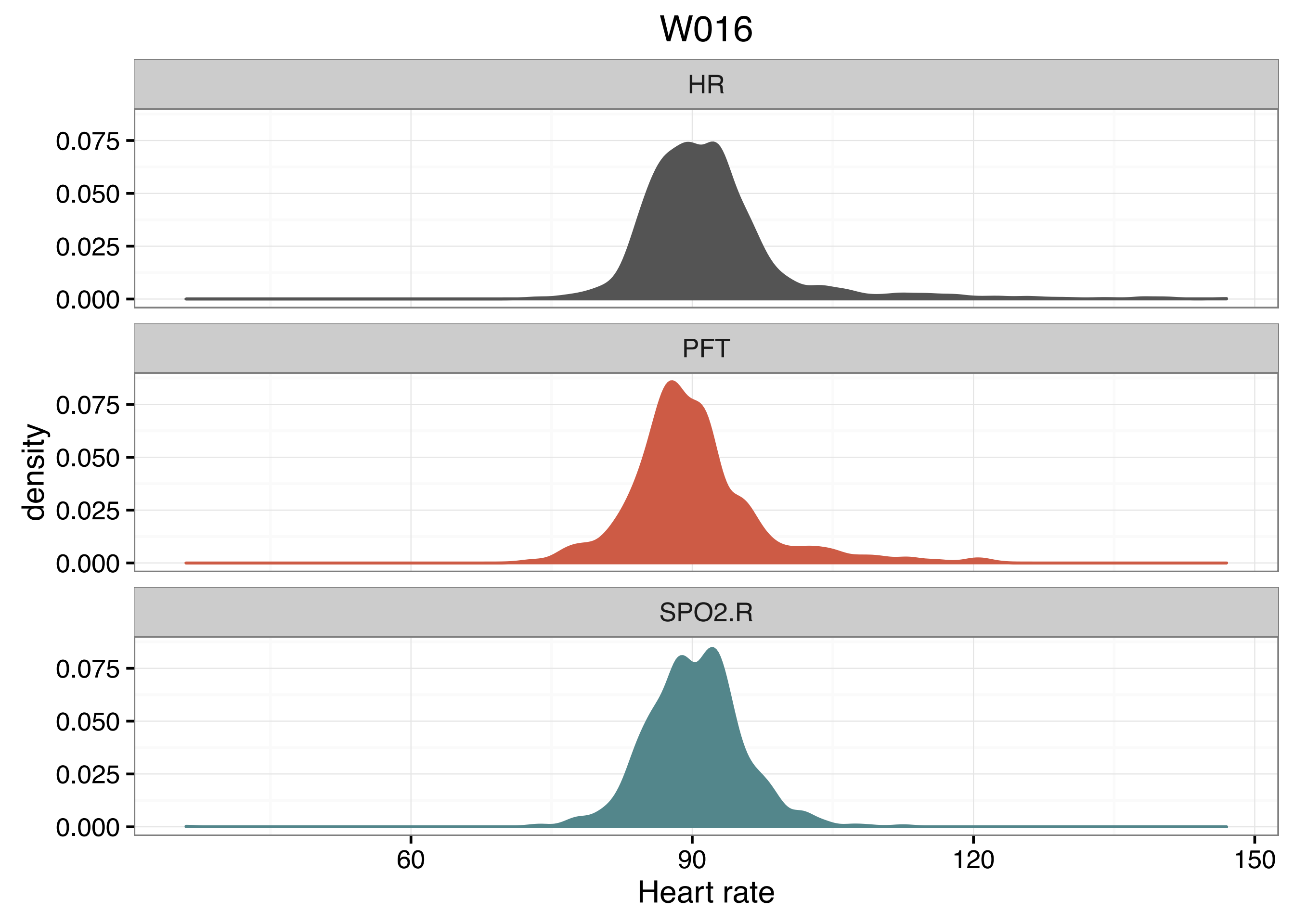

W016
HR
PFT
SPO2.R
density
0.075
0.050
0.025
0.000
60
90
120
150
Heart rate

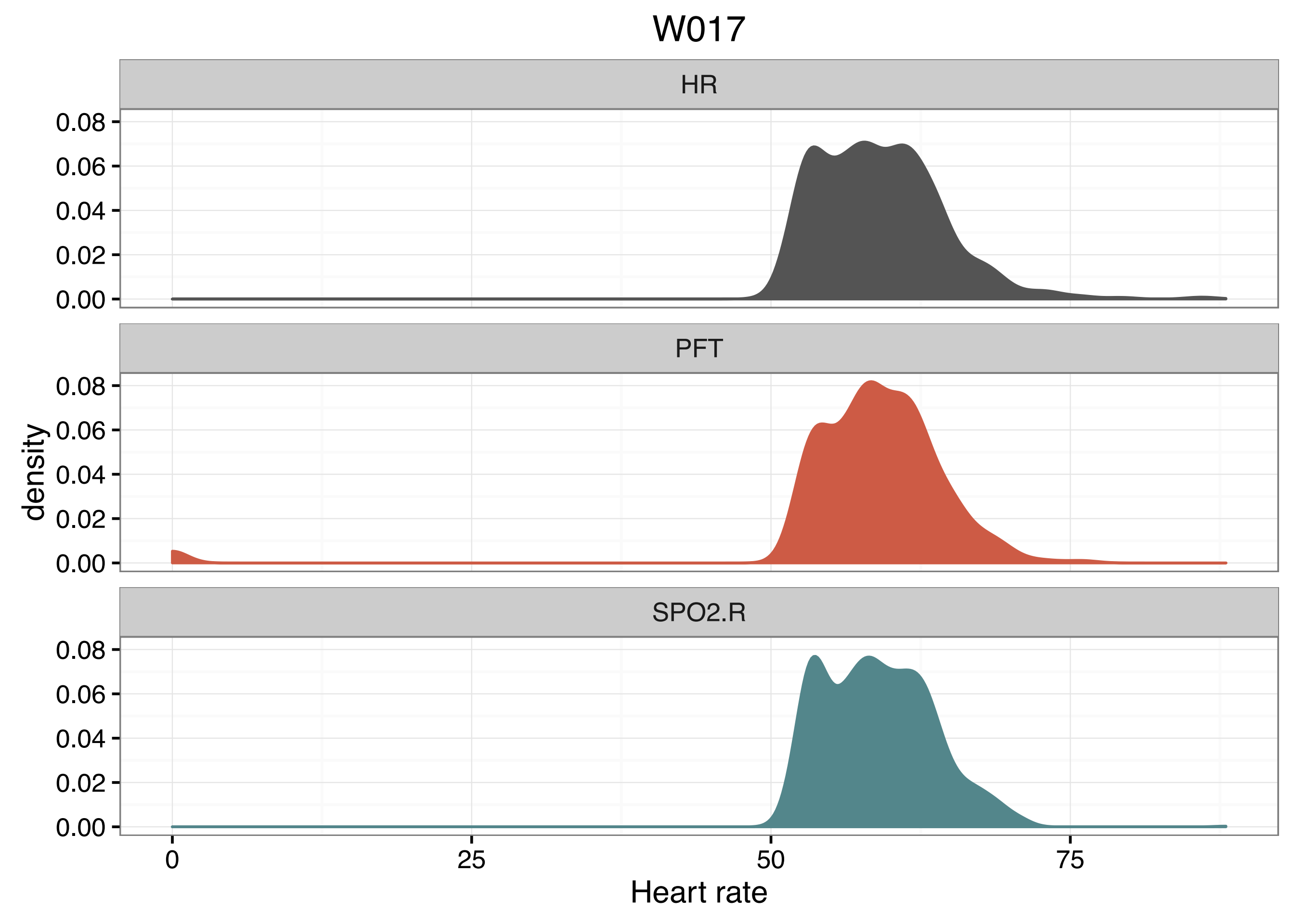

W017
HR
PFT
SPO2.R
density
0.08
0.06
0.04
0.02
0.00
0
25
50
75
Heart rate

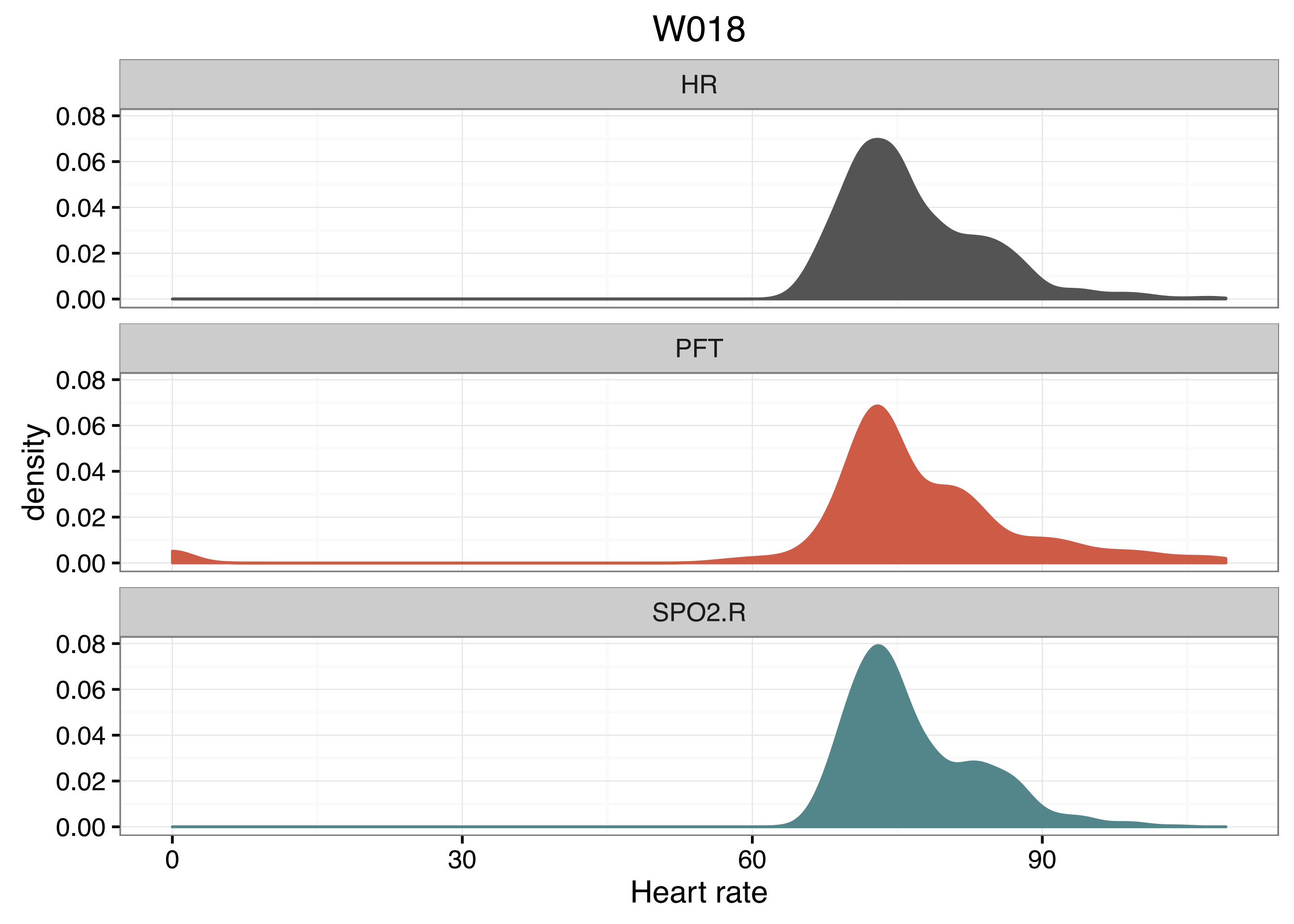

W018
HR
PFT
SPO2.R
density
0.08
0.06
0.04
0.02
0.00
0
30
60
90
Heart rate

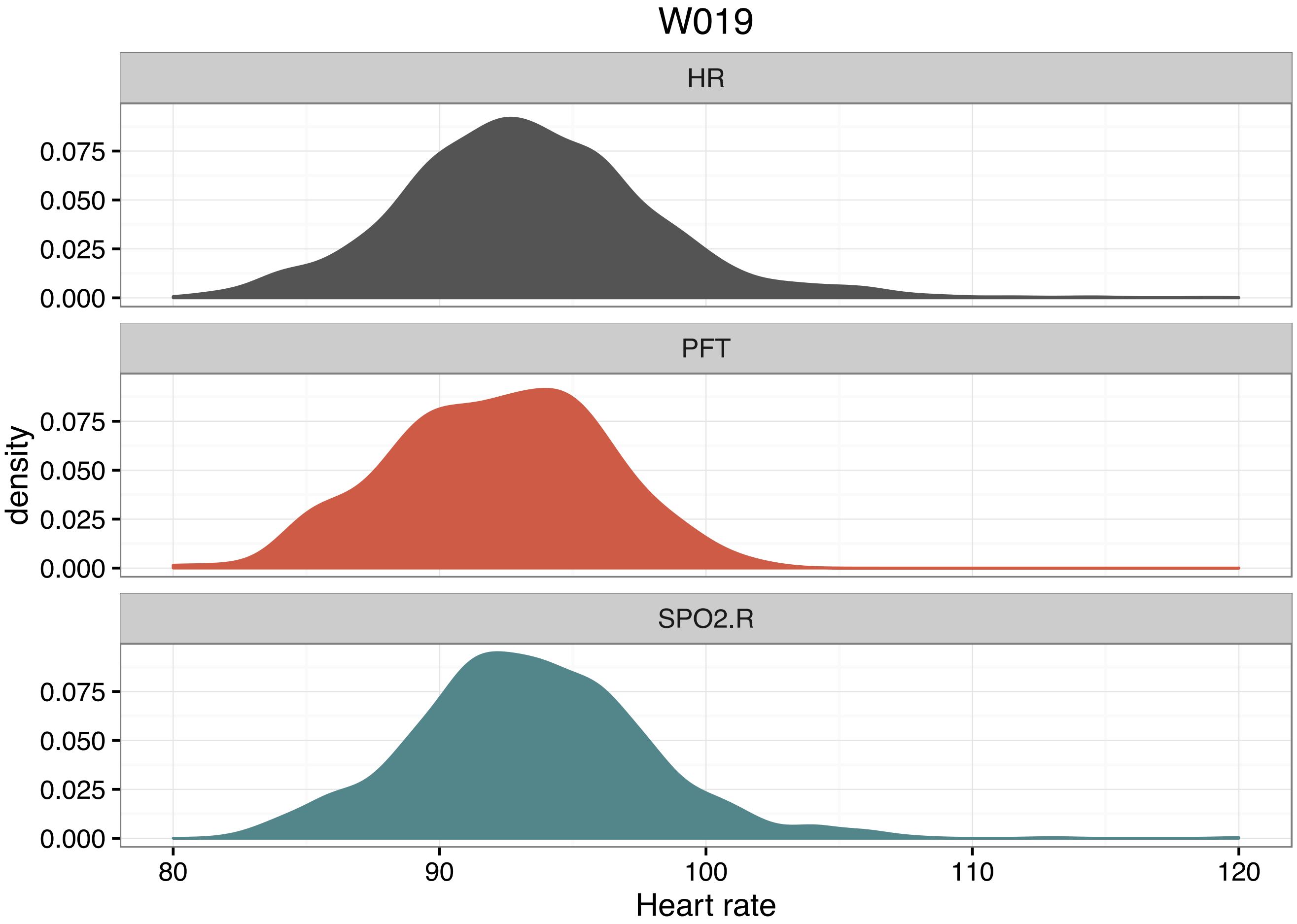

W019
HR
PFT
SPO2.R
density
0.075
0.050
0.025
0.000
80
90
100
110
120
Heart rate

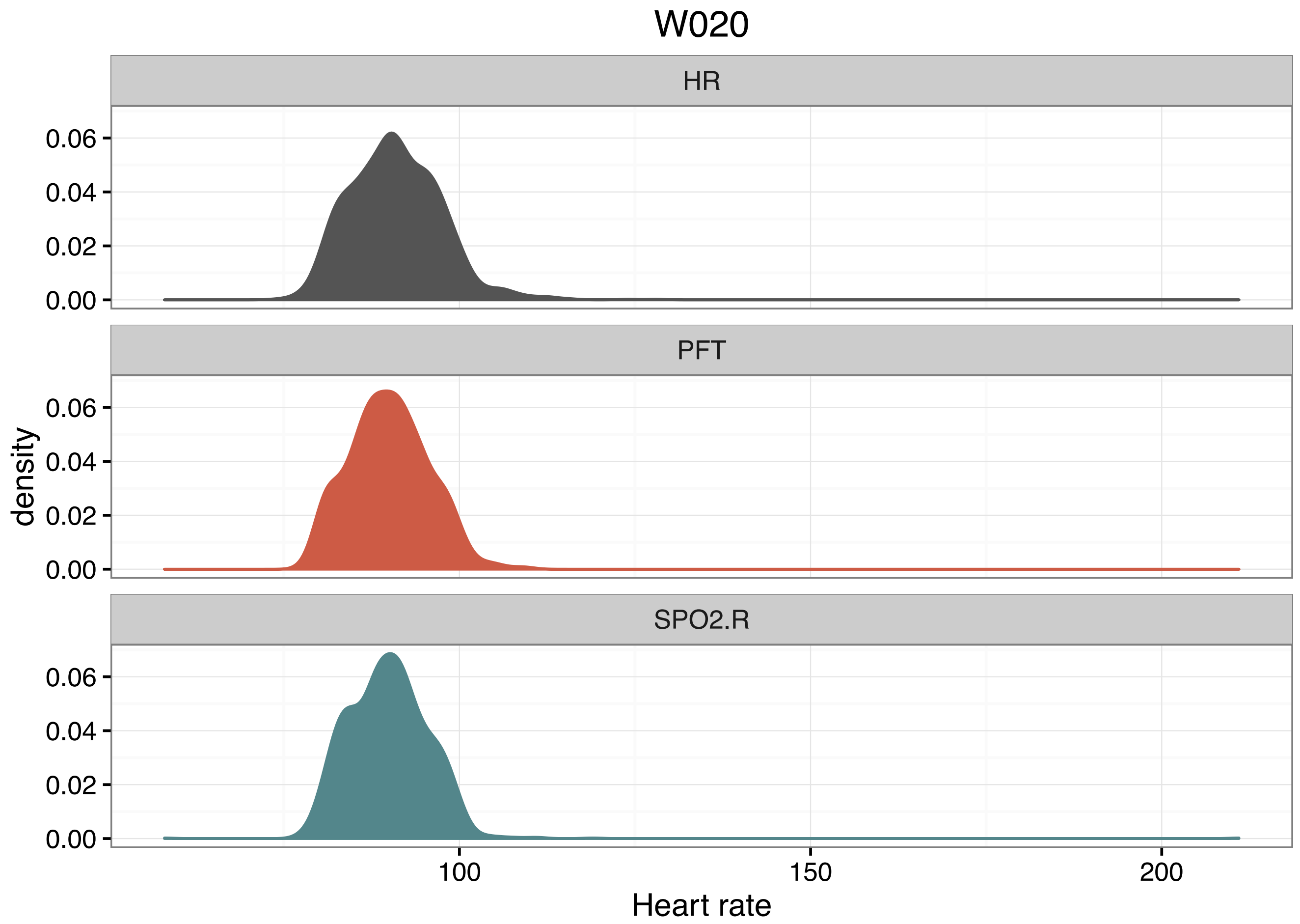

W020
HR
PFT
SPO2.R
density
0.06
0.04
0.02
0.00
100
150
200
Heart rate

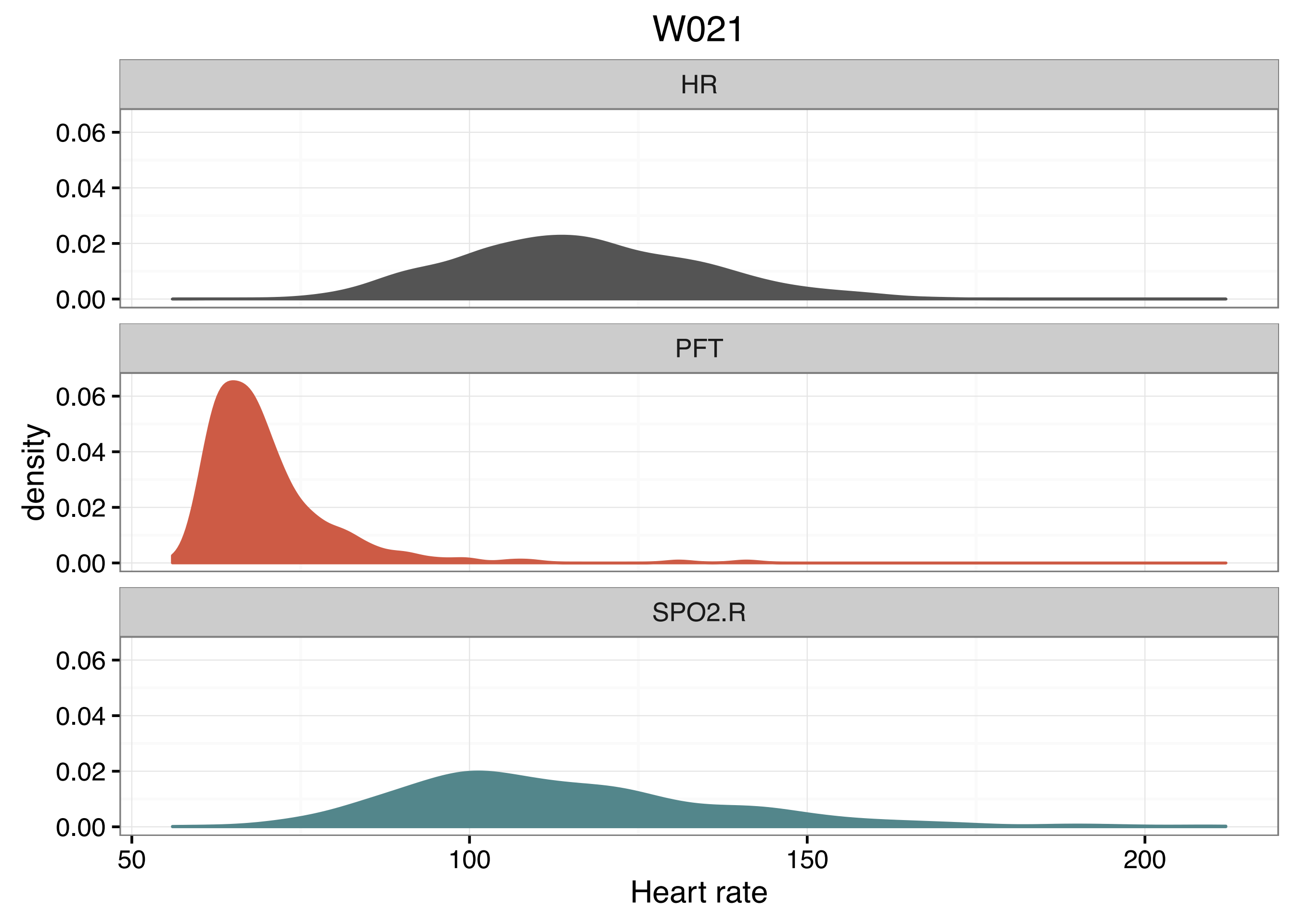

W021
HR
PFT
SPO2.R
0.06
0.04
0.02
0.00
density
50
100
150
200
Heart rate

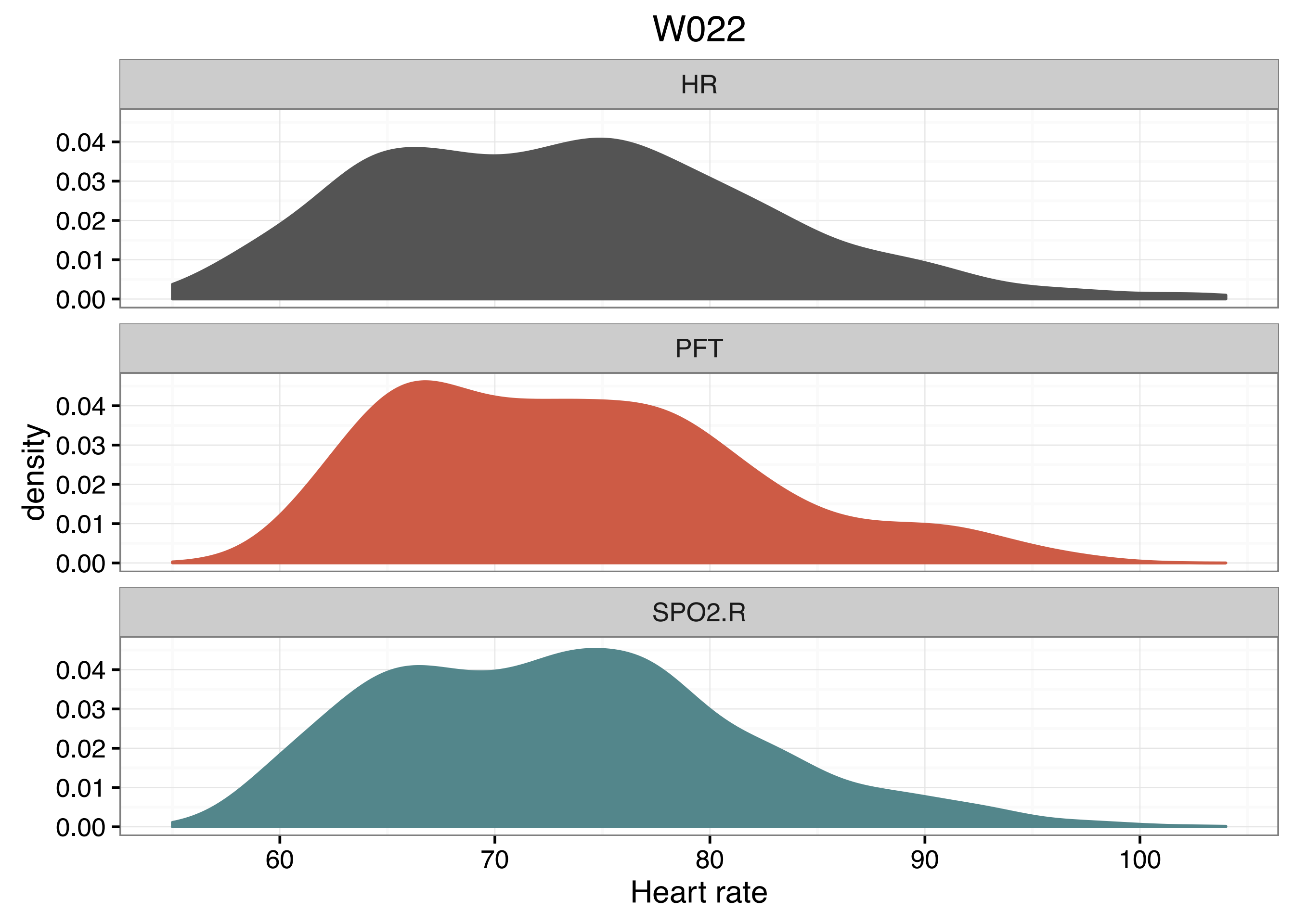
W022
HR
PFT
SPO2.R
density
0.00
0.01
0.02
0.03
0.04
60
70
80
90
100
Heart rate

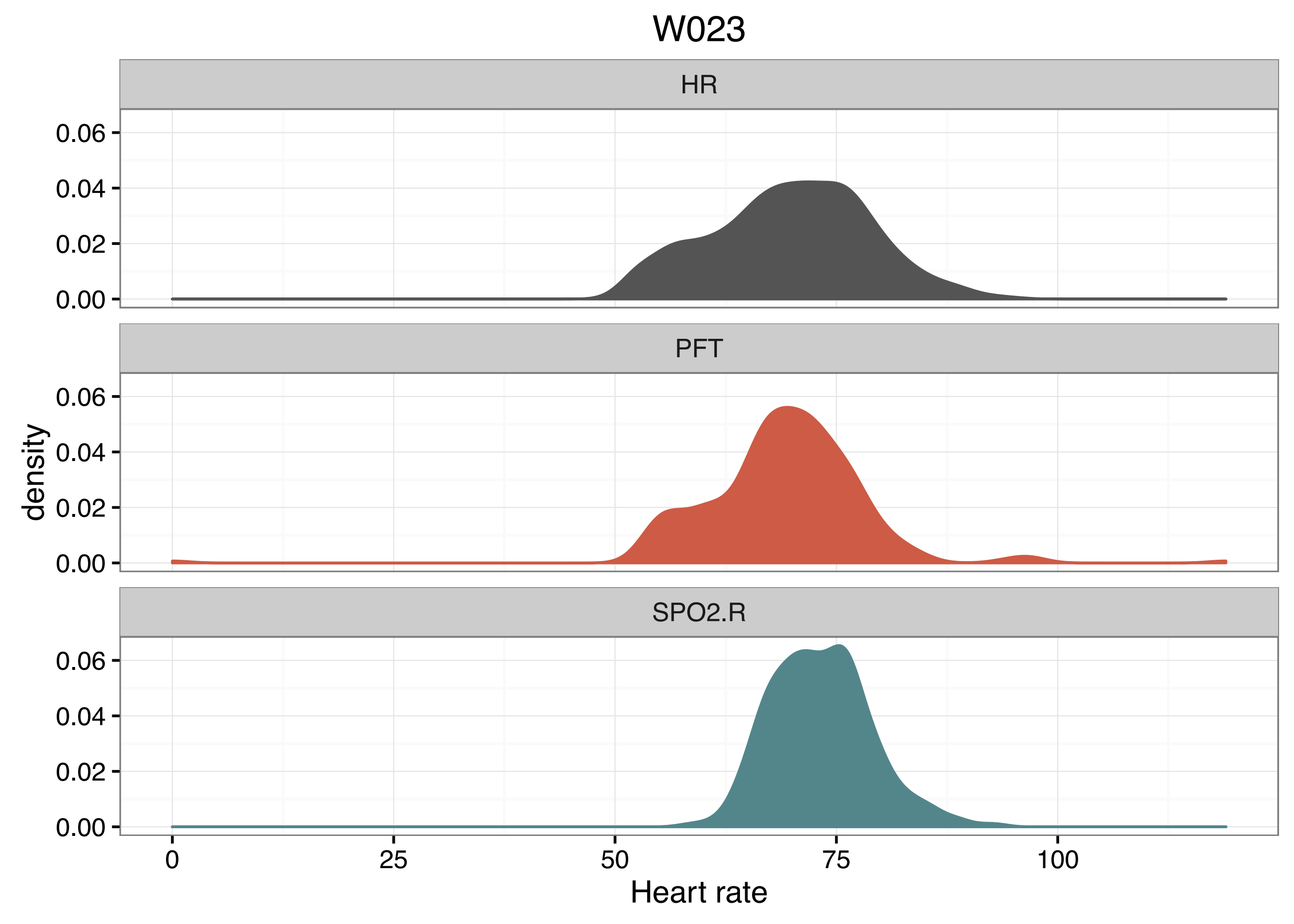
W023
HR
PFT
SPO2.R
density
0.00
0.02
0.04
0.06
0
25
50
75
100
Heart rate

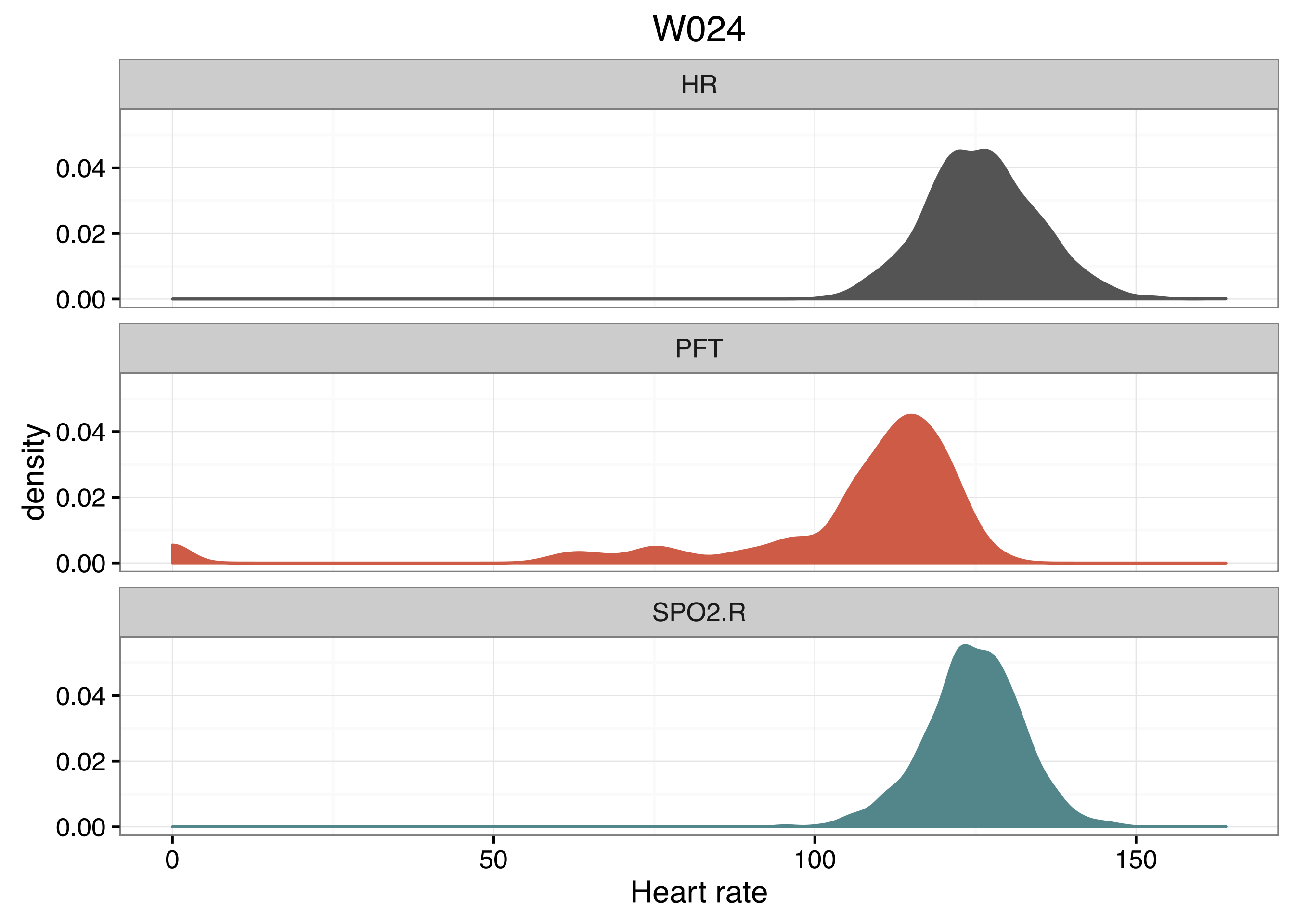
W024
HR
PFT
SPO2.R
density
0.04
0.02
0.00
0
50
100
150
Heart rate

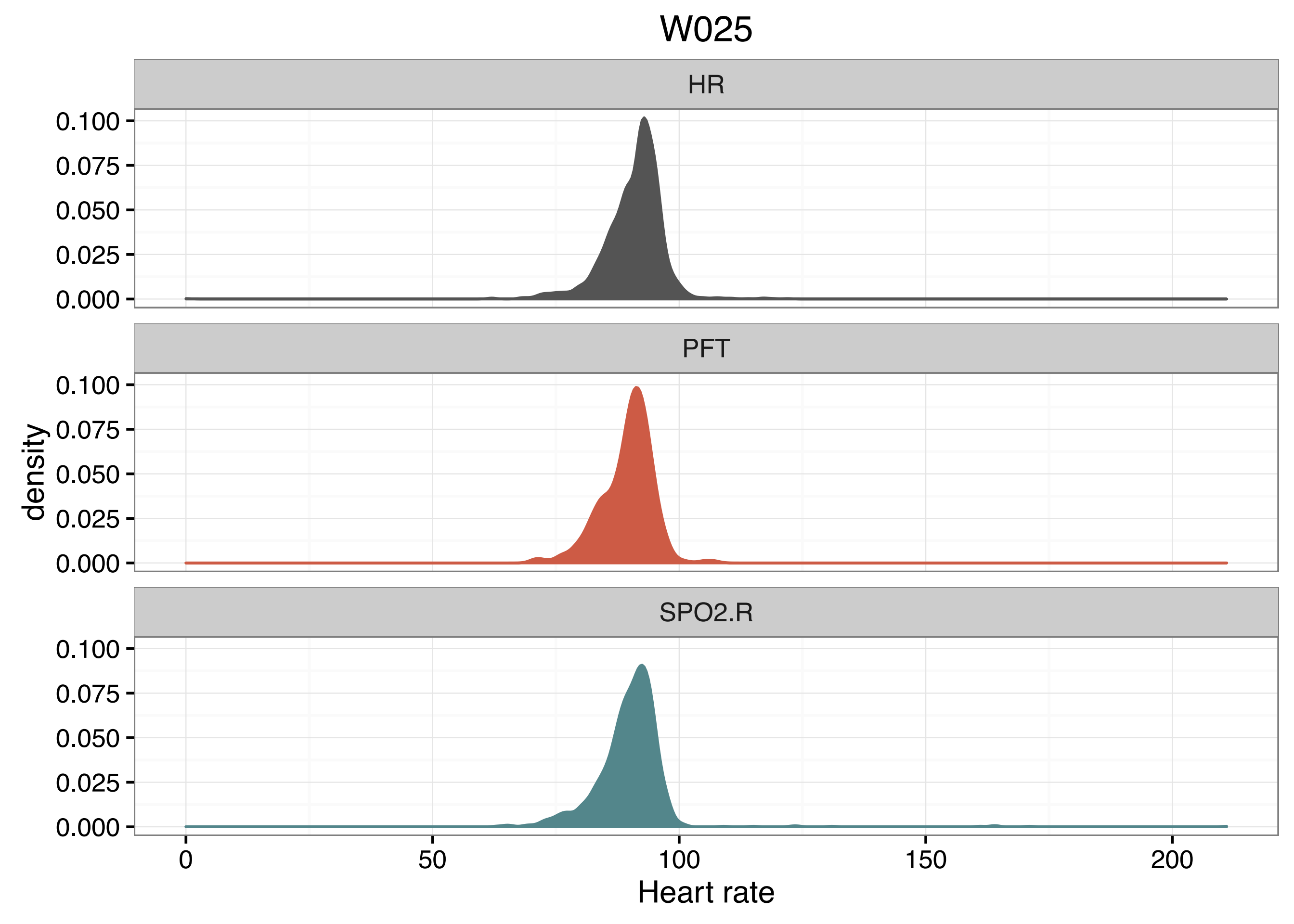
W025
HR
PFT
SPO2.R
density
0.100
0.075
0.050
0.025
0.000
0
50
100
150
200
Heart rate

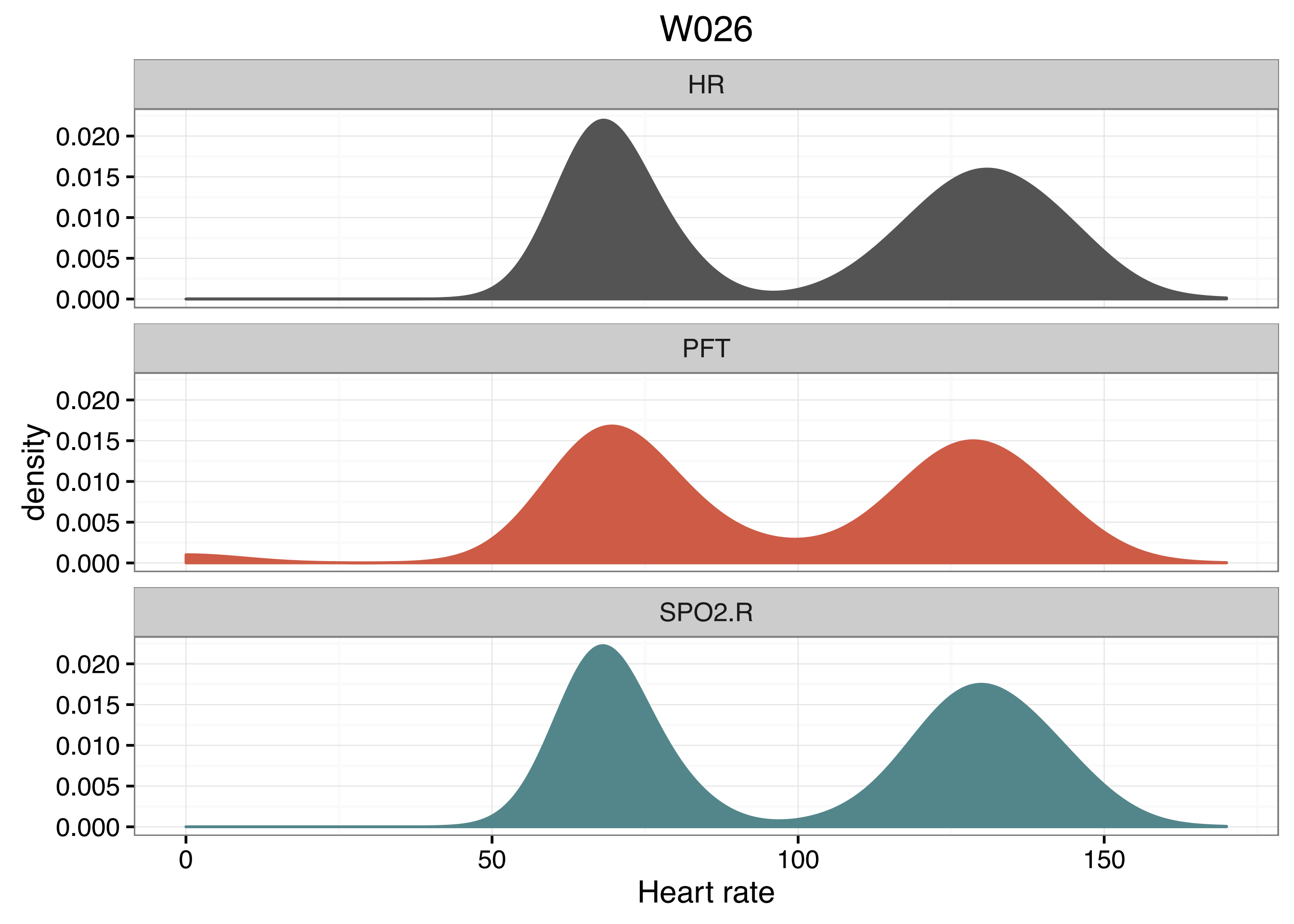
W026
HR
PFT
SPO2.R
density
0.020
0.015
0.010
0.005
0.000
0
50
100
150
Heart rate

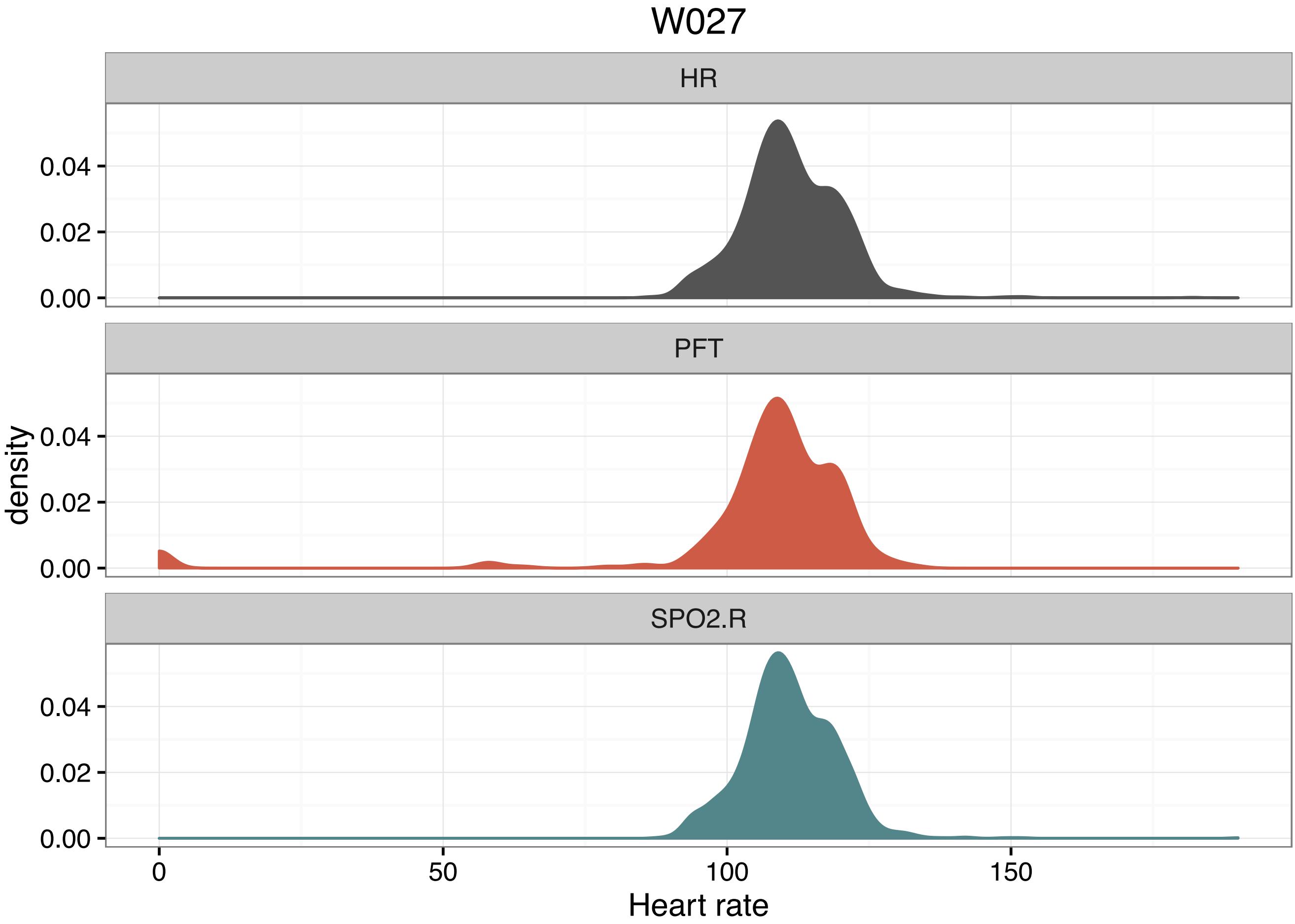
W027
HR
PFT
SPO2.R
density
0.04
0.02
0.00
0
50
100
150
Heart rate

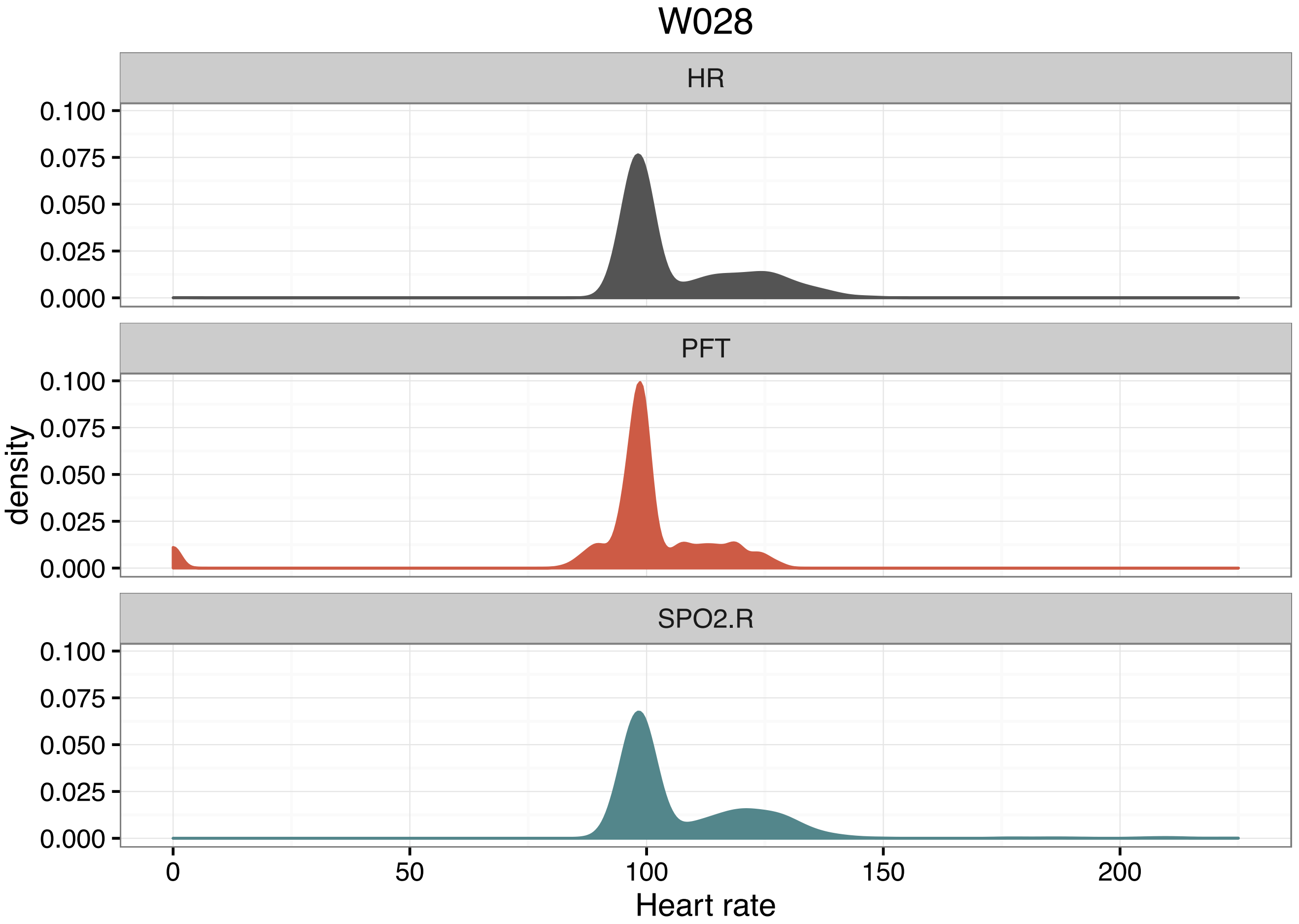
W028
HR
PFT
SPO2.R
density
0.100
0.075
0.050
0.025
0.000
0
50
100
150
200
Heart rate

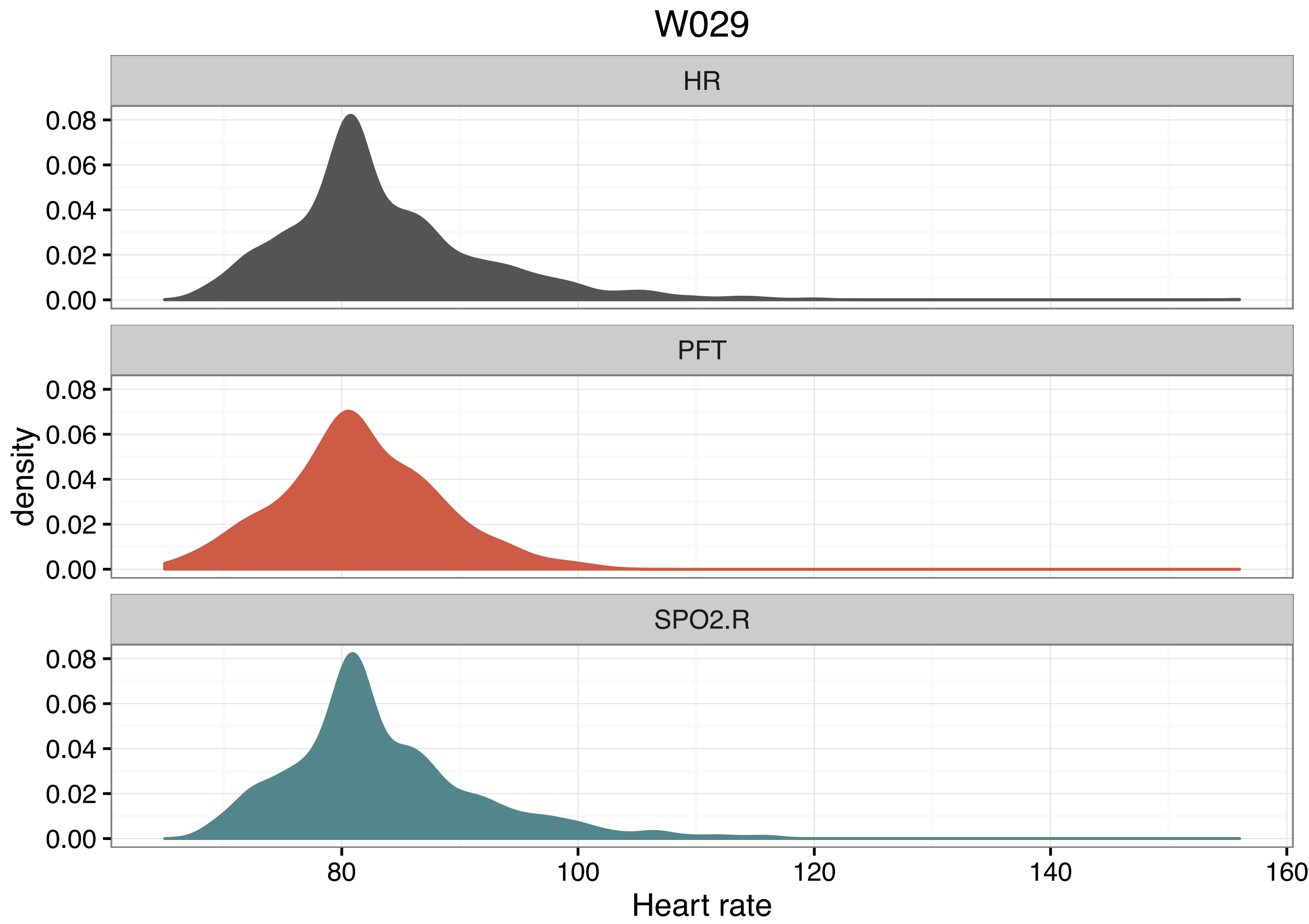
W029
HR
PFT
SPO2.R
density
0.08
0.06
0.04
0.02
0.00
80
100
120
140
160
Heart rate

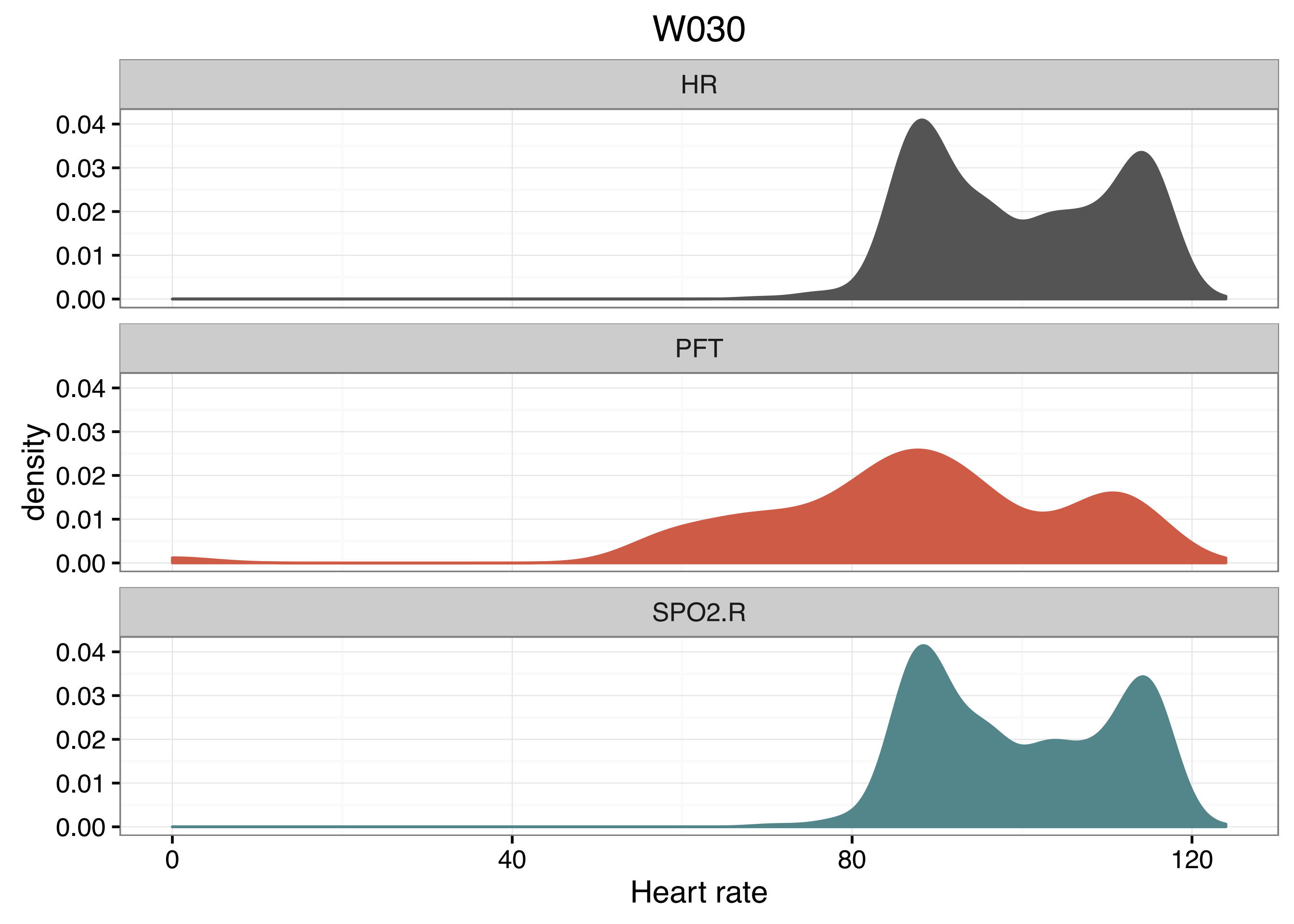
W030
HR
PFT
SPO2.R
density
0.04
0.03
0.02
0.01
0.00
0
40
80
120
Heart rate

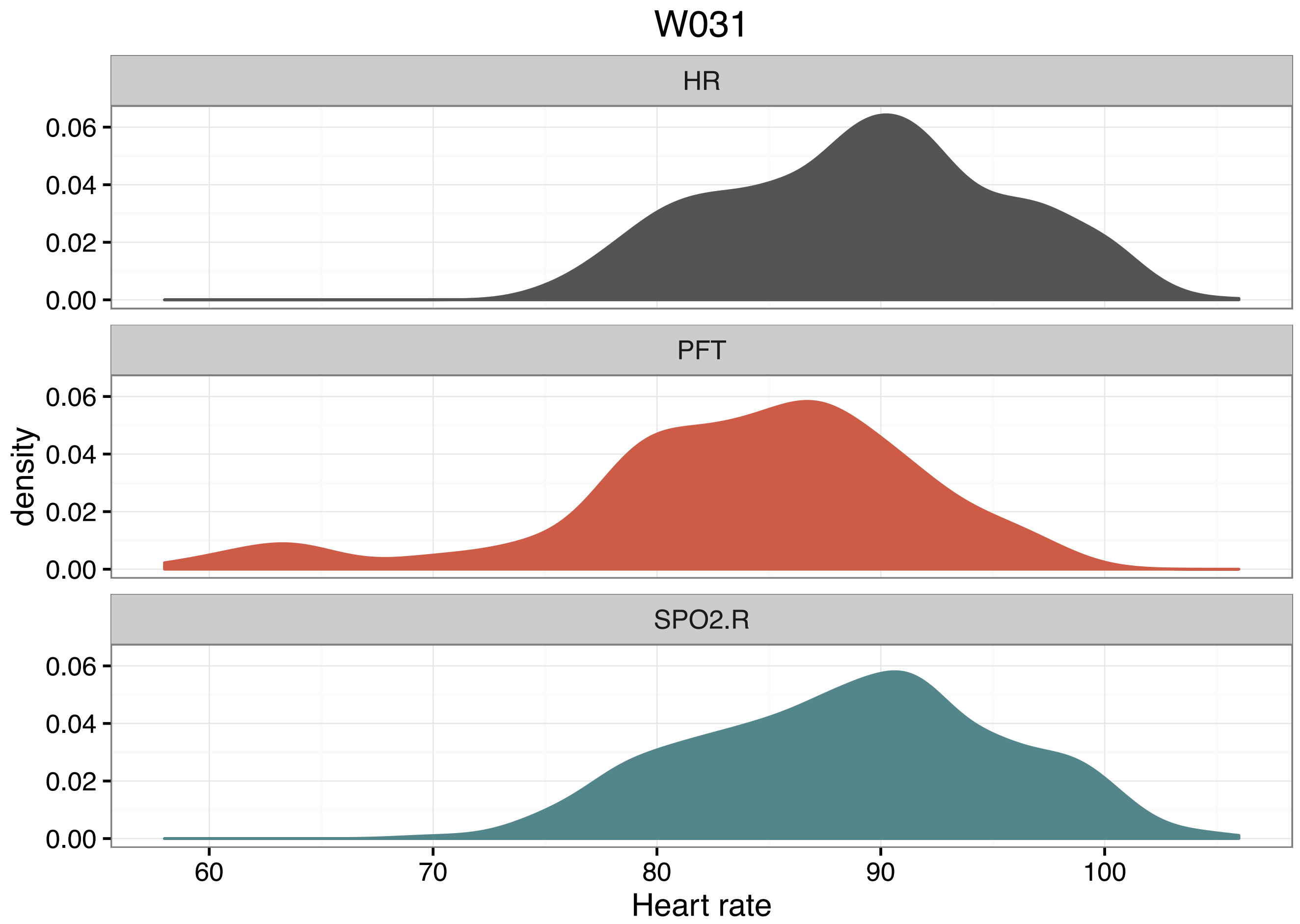
W031
HR
PFT
SPO2.R
density
0.00
0.02
0.04
0.06
60
70
80
90
100
Heart rate

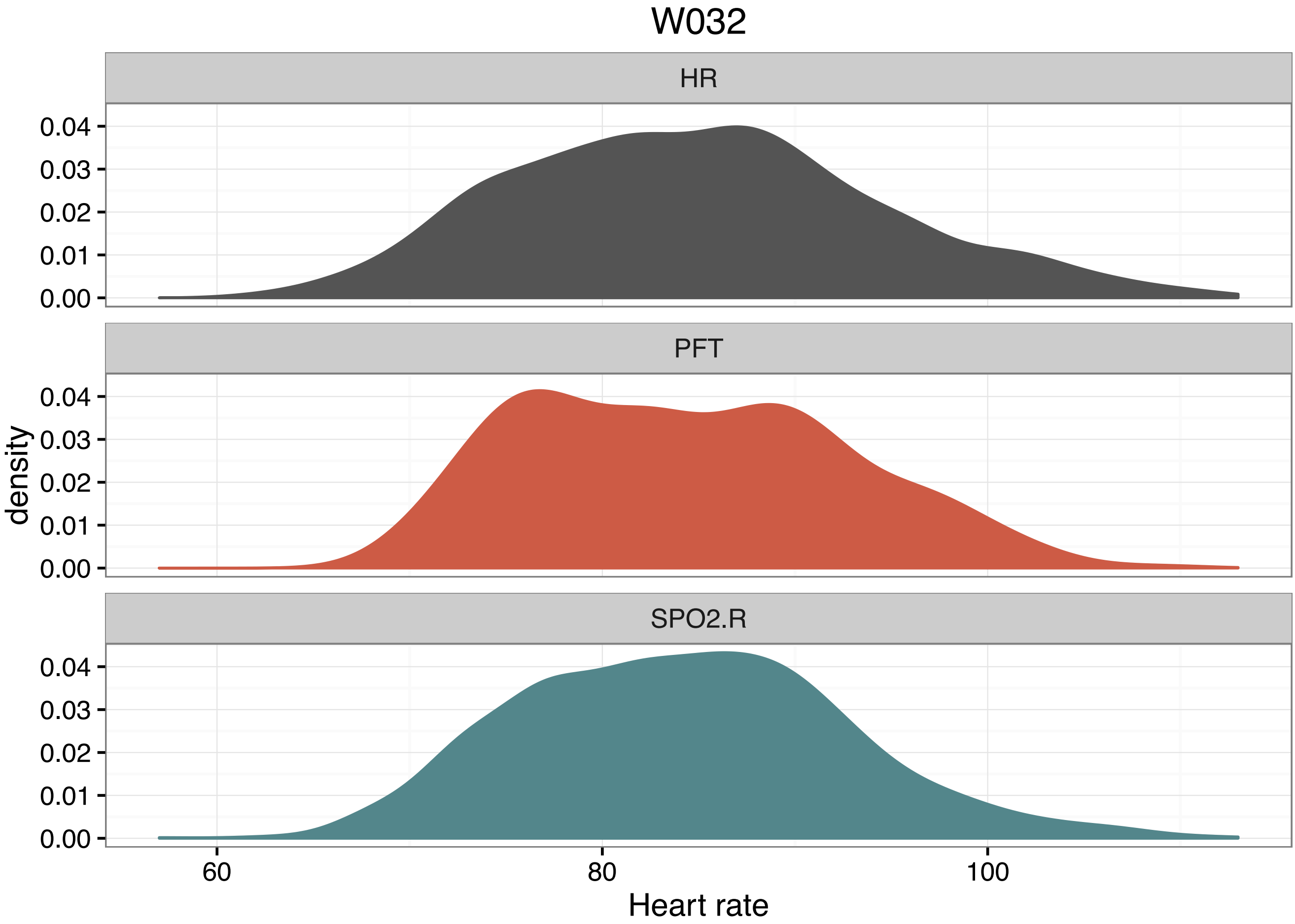

W032
HR
PFT
SPO2.R
density
0.04
0.03
0.02
0.01
0.00
60
80
100
Heart rate

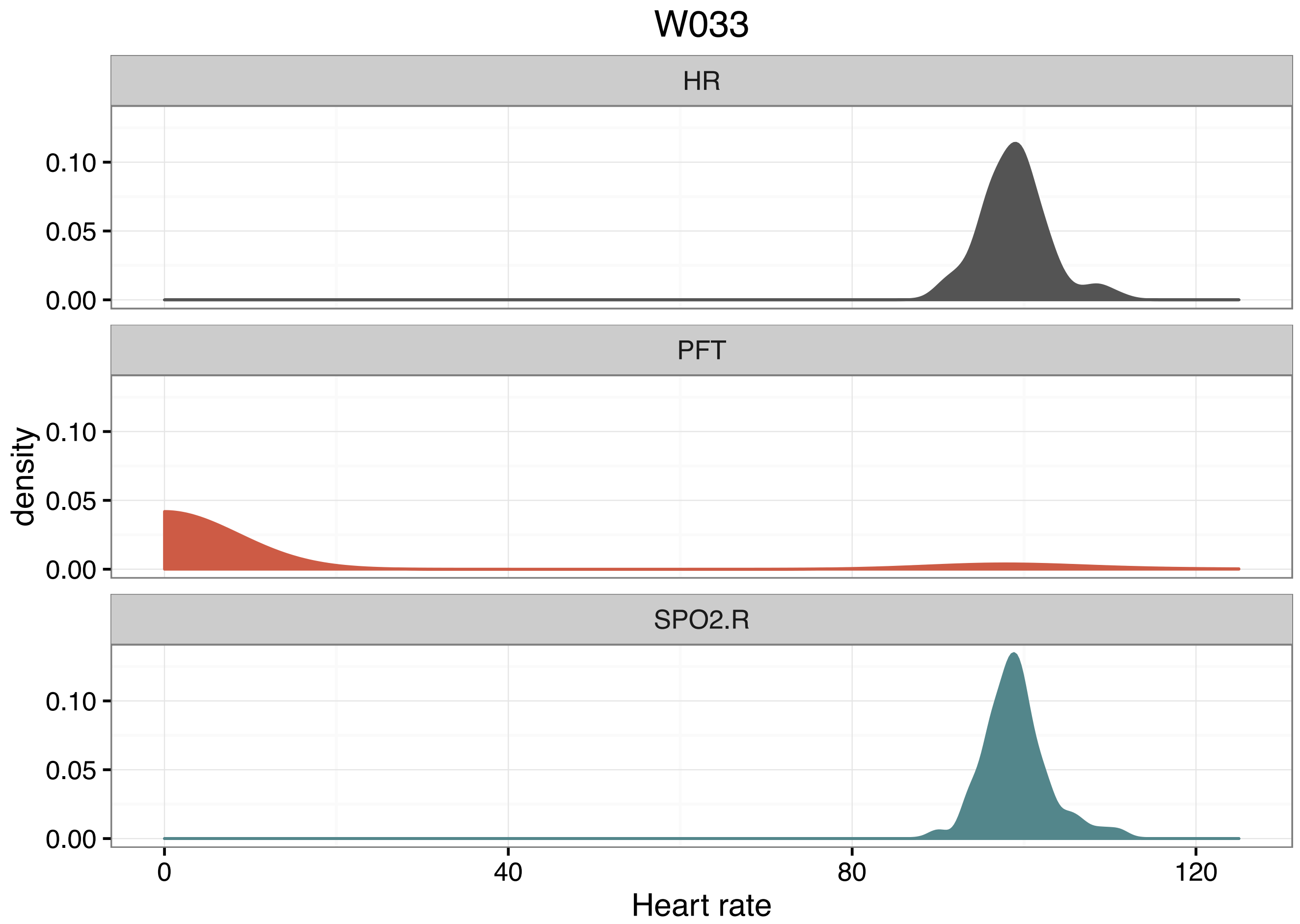

W033
HR
PFT
SPO2.R
density
0.00
0.05
0.10
0
40
80
120
Heart rate

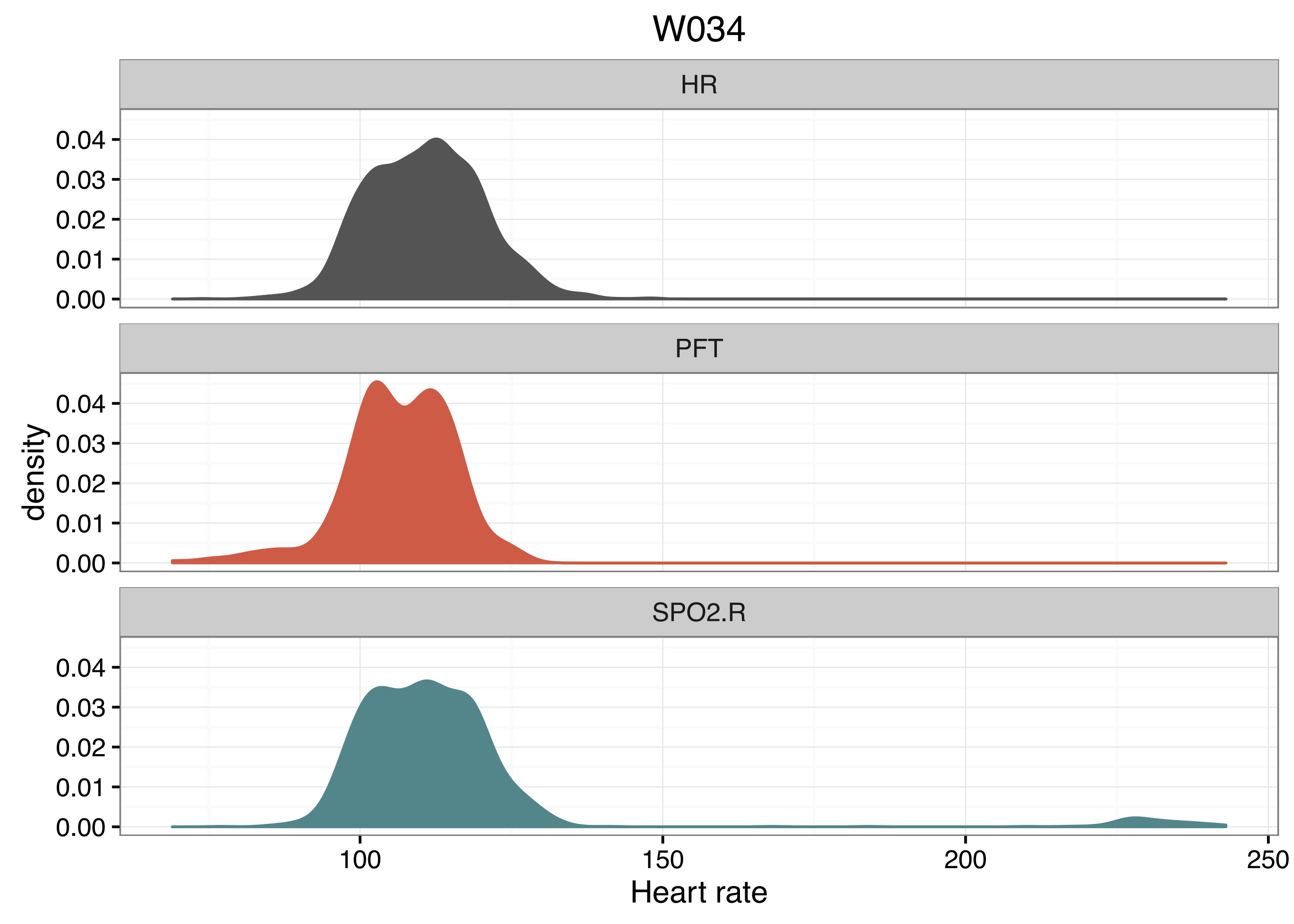
W034
HR
PFT
SPO2.R
density
0.00
0.01
0.02
0.03
0.04
100
150
200
250
Heart rate

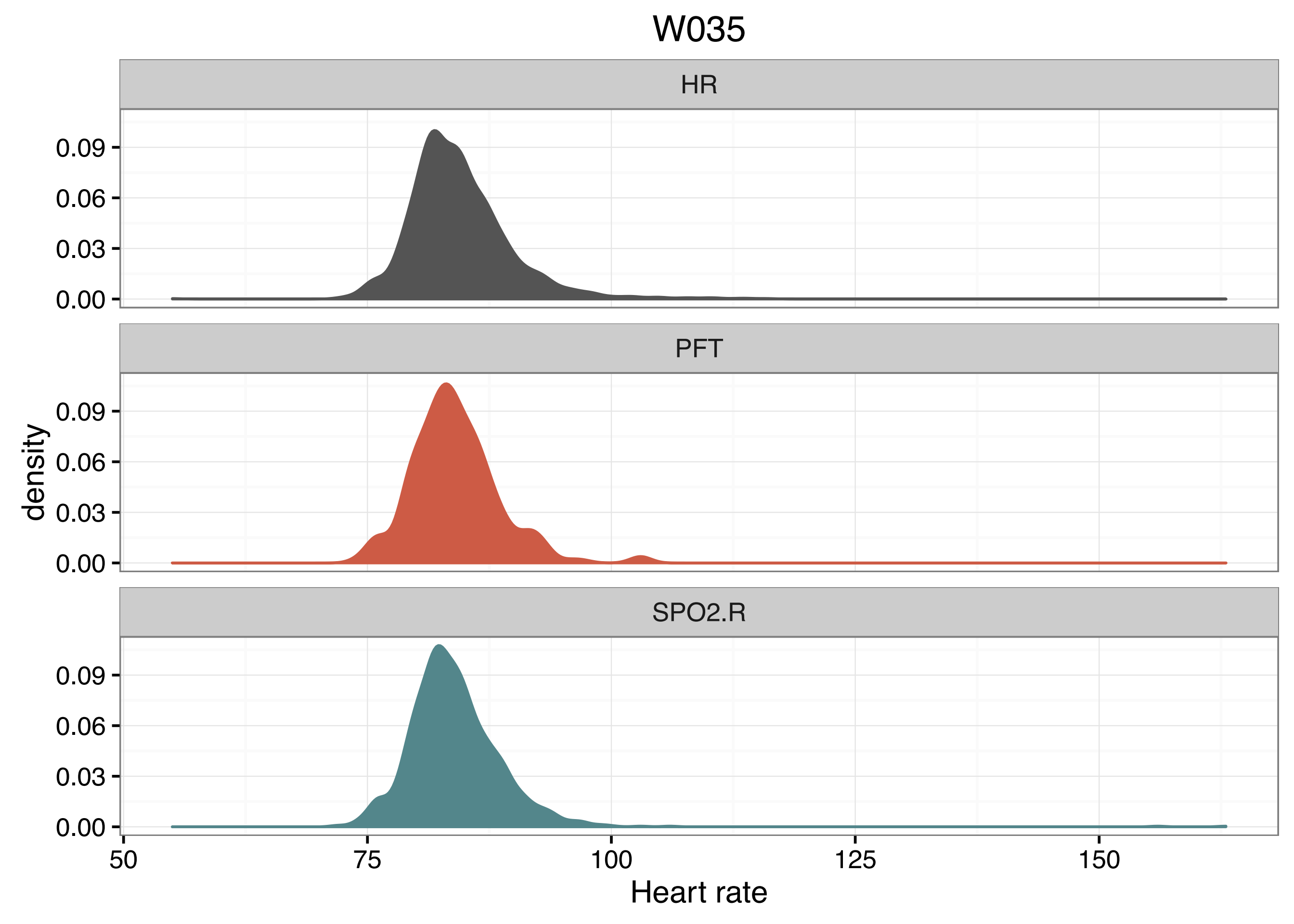
W035
HR
PFT
SPO2.R
density
0.00
0.03
0.06
0.09
50
75
100
125
150
Heart rate

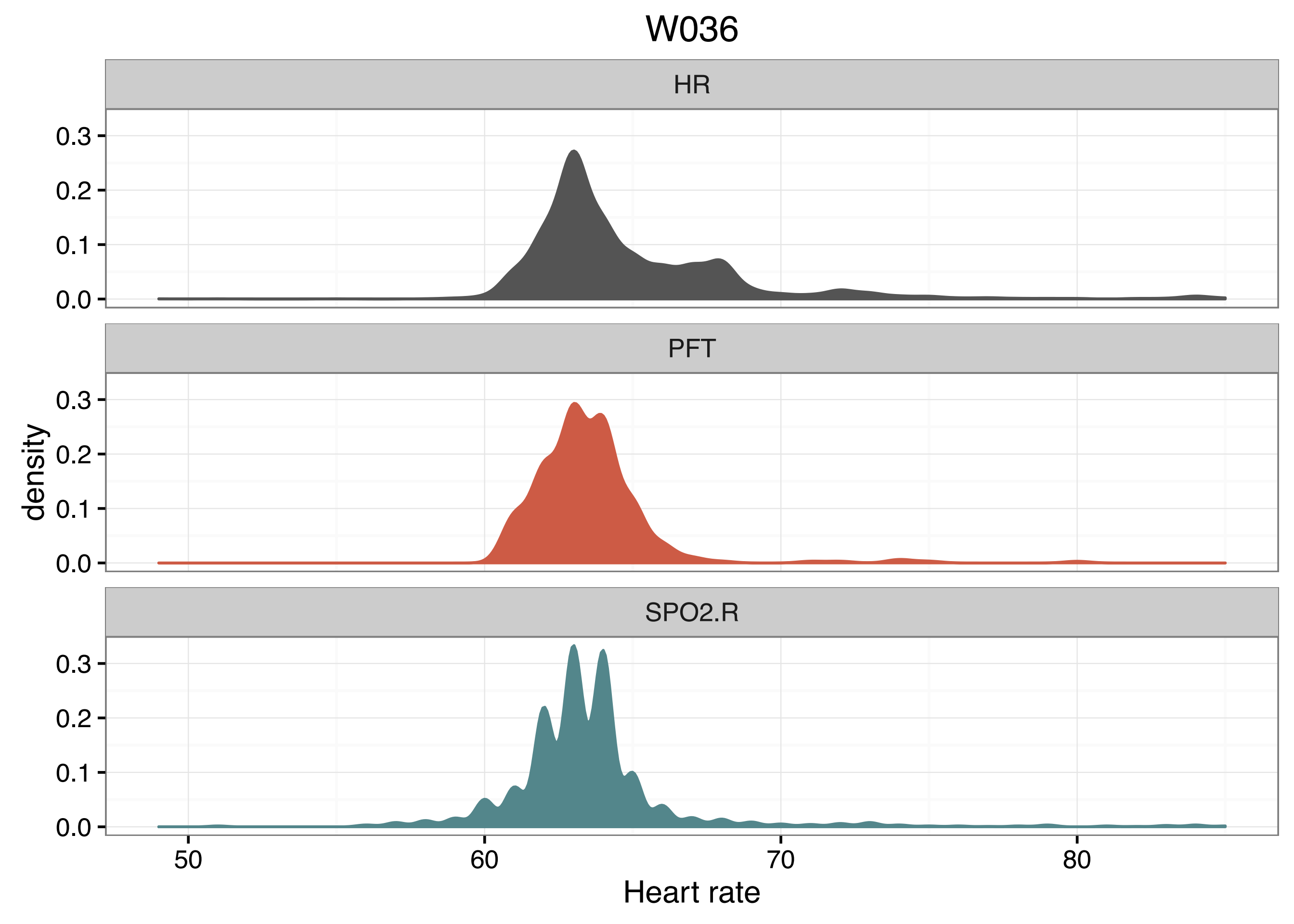
W036
HR
PFT
SPO2.R
density
0.0
0.1
0.2
0.3
50
60
70
80
Heart rate

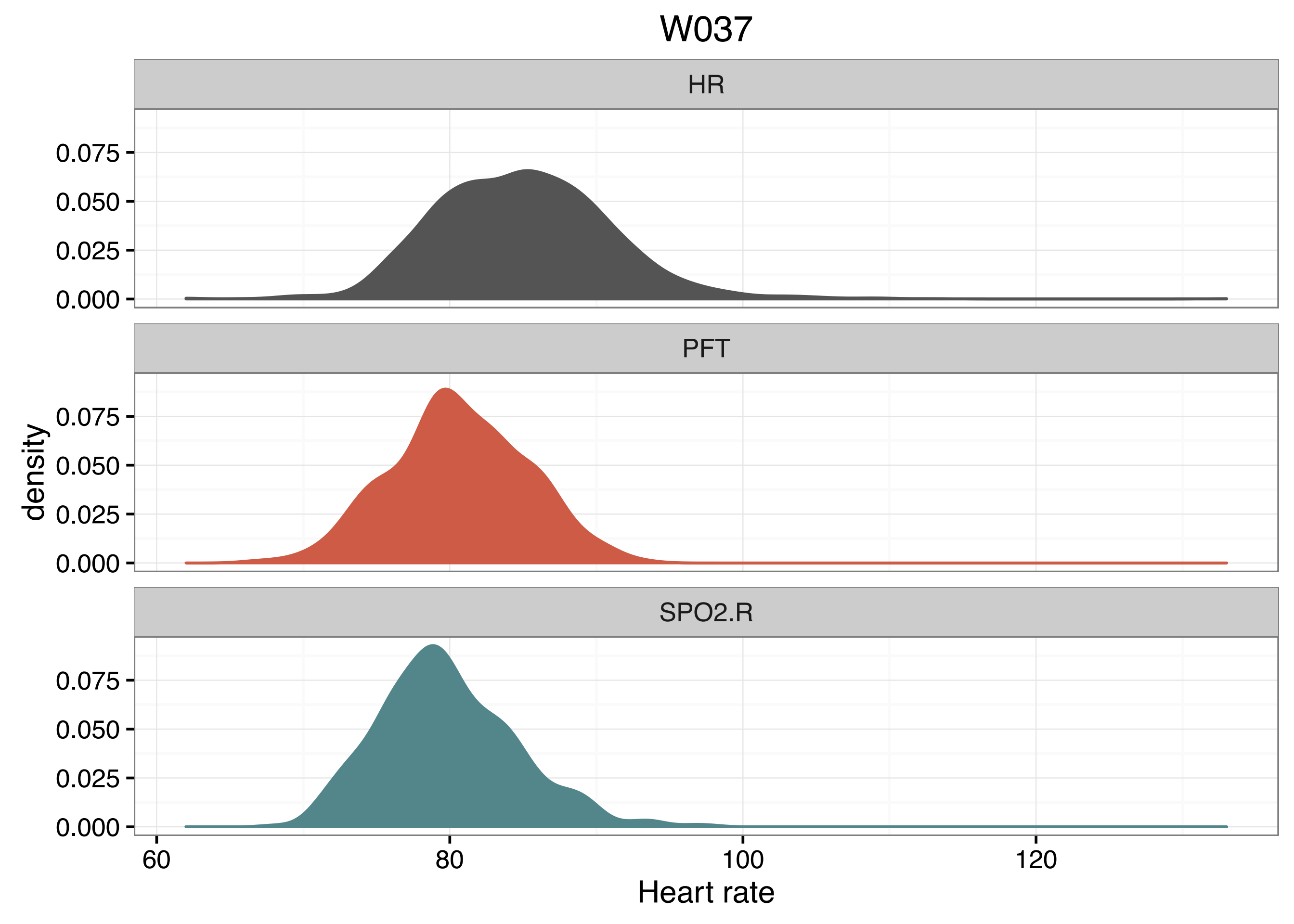
W037
HR
PFT
SPO2.R
density
0.075
0.050
0.025
0.000
60
80
100
120
Heart rate

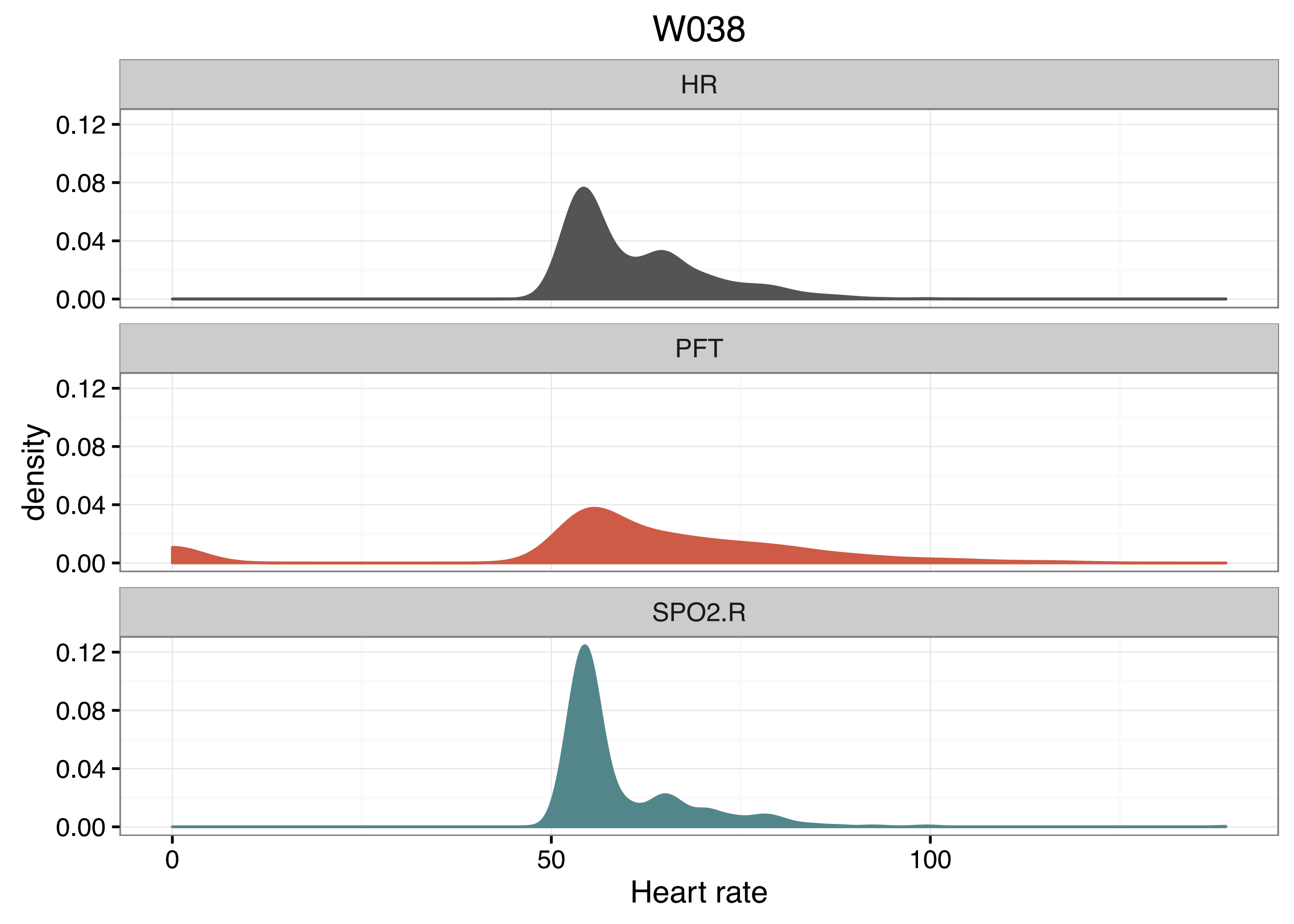

W038
HR
PFT
SPO2.R
density
0.12
0.08
0.04
0.00
0
50
100
Heart rate

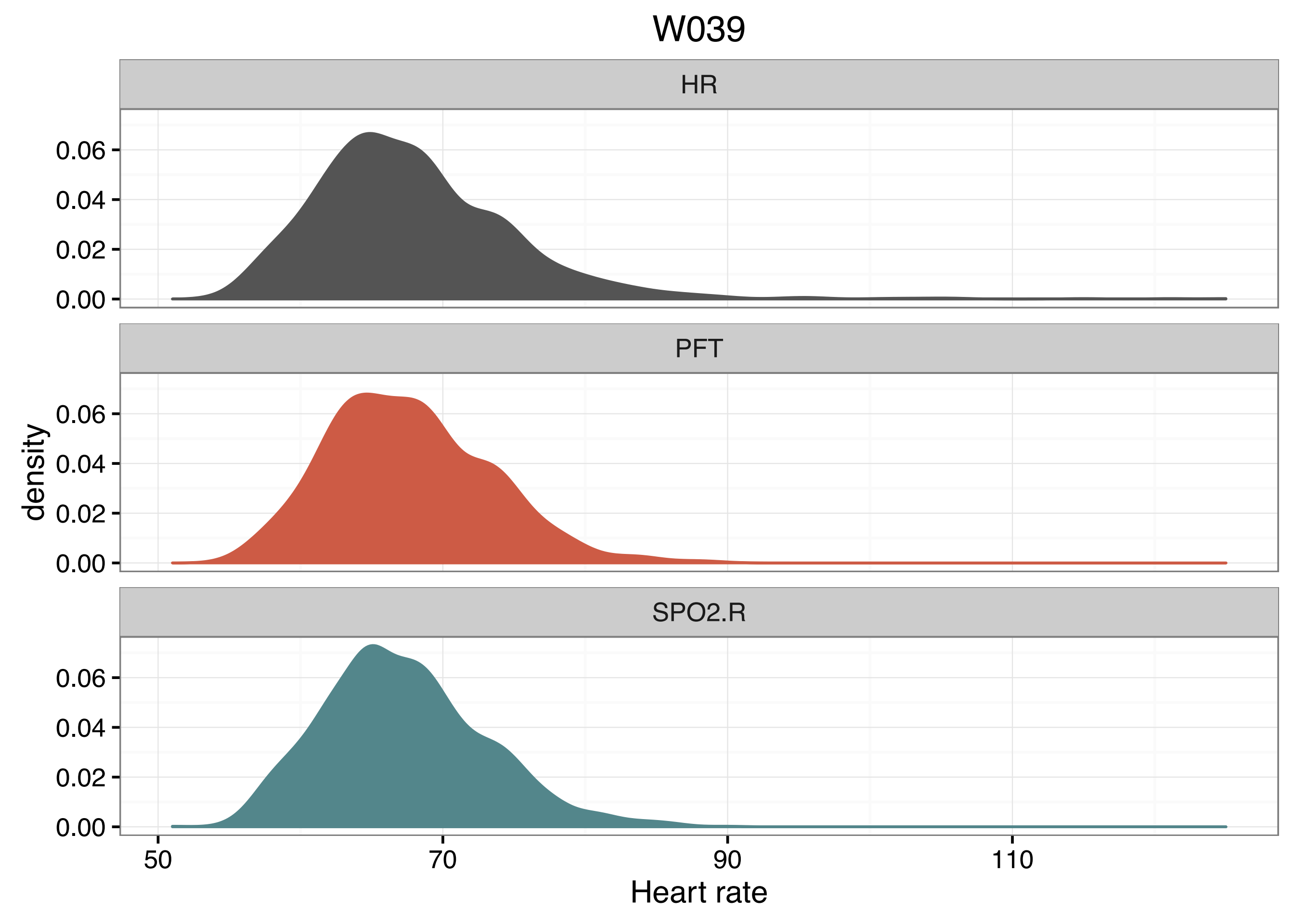
W039
HR
PFT
SPO2.R
density
0.06
0.04
0.02
0.00
50
70
90
110
Heart rate

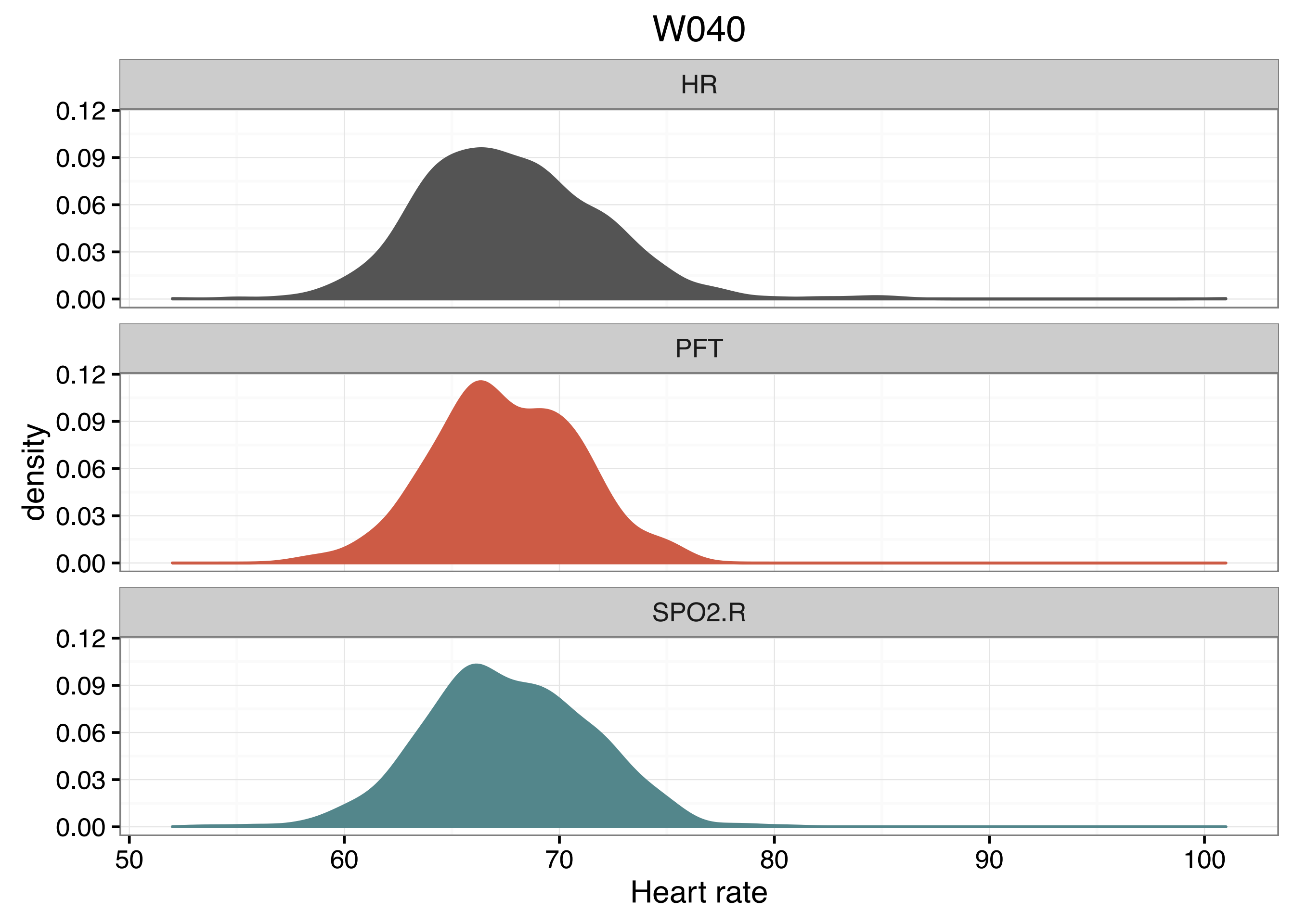
W040
HR
PFT
SPO2.R
density
0.12
0.09
0.06
0.03
0.00
50
60
70
80
90
100
Heart rate

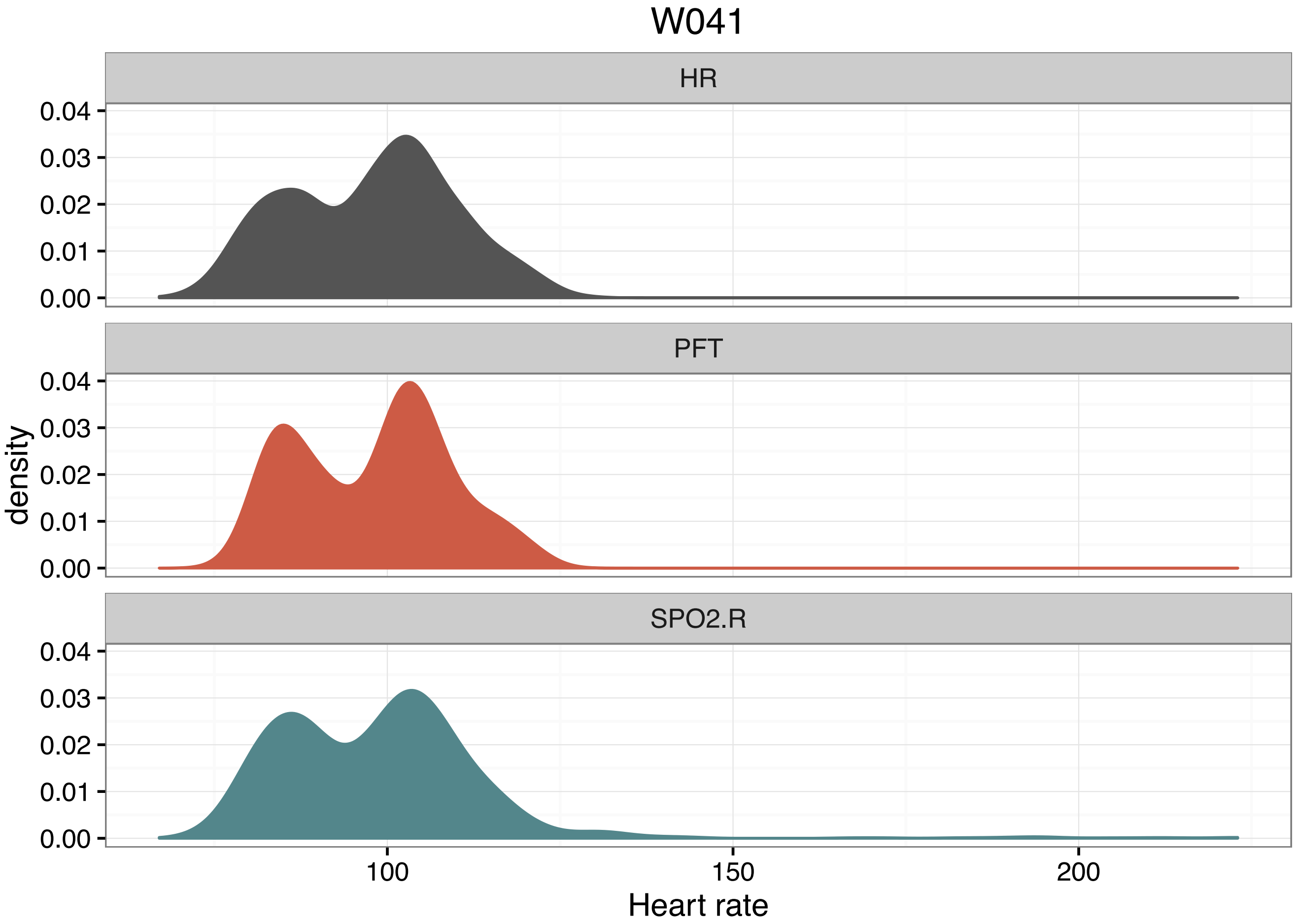

W041
HR
PFT
SPO2.R
density
0.04
0.03
0.02
0.01
0.00
100
150
200
Heart rate

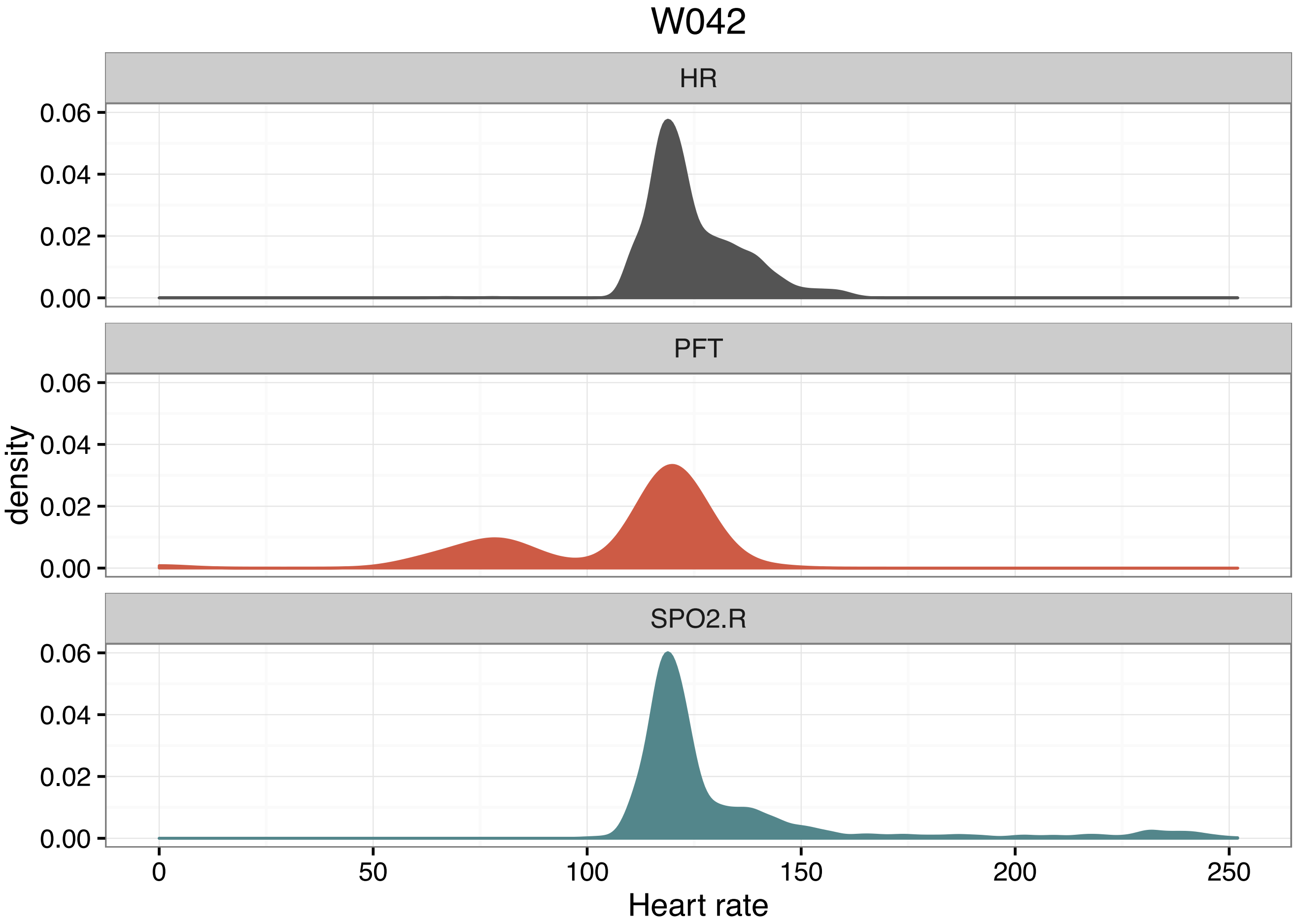
W042
HR
PFT
SPO2.R
density
0.06
0.04
0.02
0.00
0
50
100
150
200
250
Heart rate

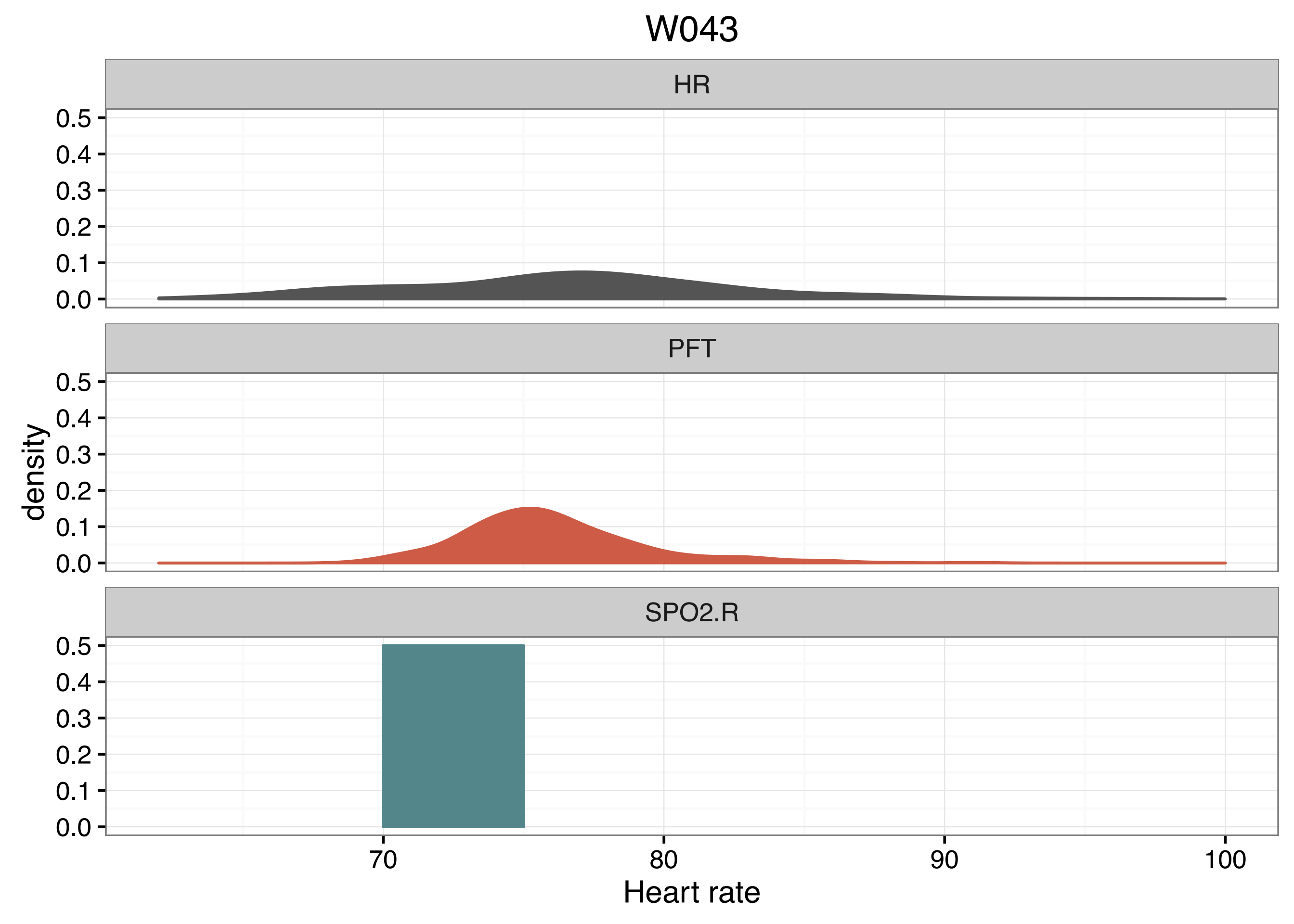
W043
HR
PFT
SPO2.R
density
0.0
0.1
0.2
0.3
0.4
0.5
70
80
90
100
Heart rate

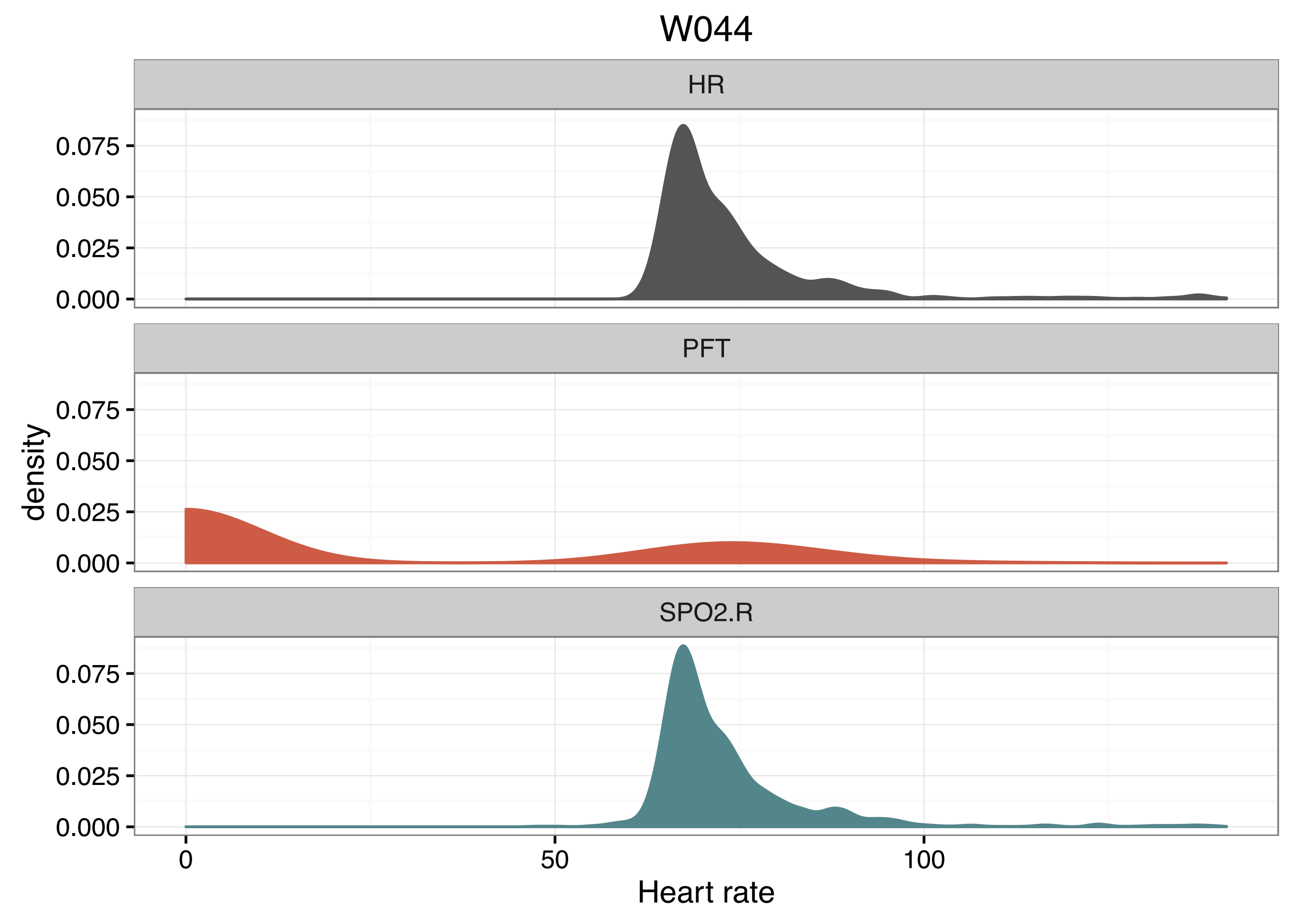
W044
HR
PFT
SPO2.R
density
0.075
0.050
0.025
0.000
0
50
100
Heart rate

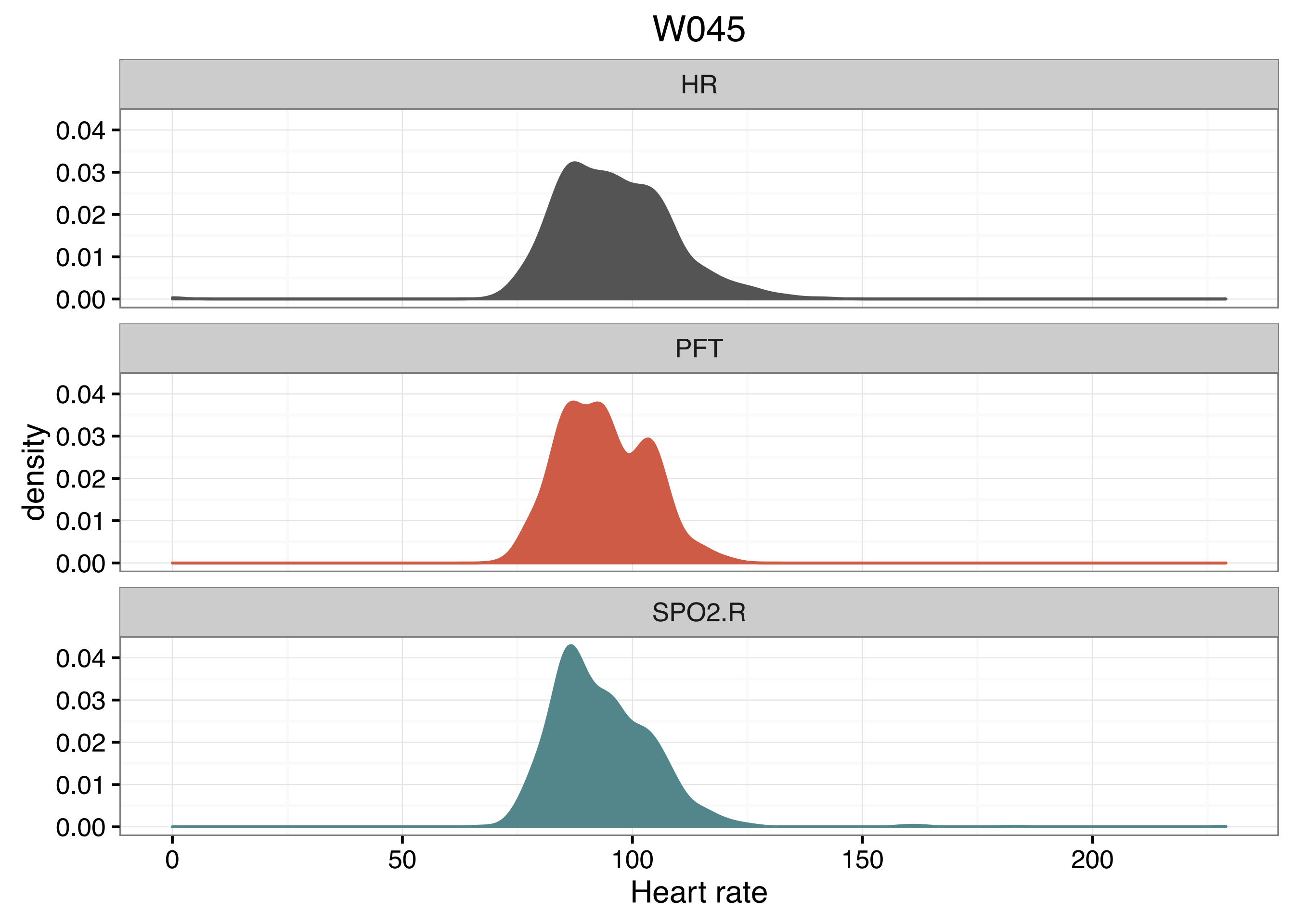
W045
HR
PFT
SPO2.R
density
0.04
0.03
0.02
0.01
0.00
0
50
100
150
200
Heart rate

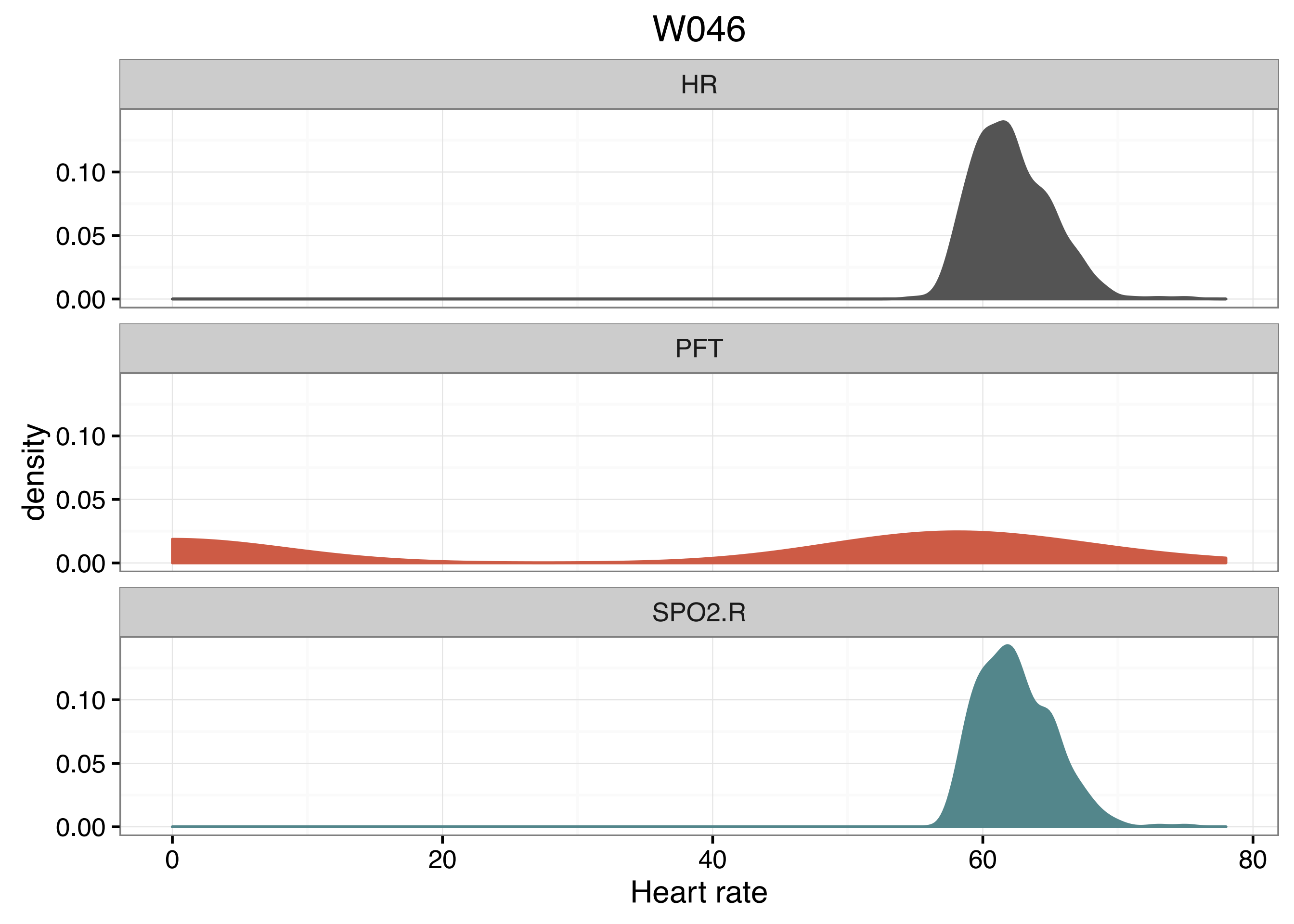

W046
HR
PFT
SPO2.R
density
0.10
0.05
0.00
0
20
40
60
80
Heart rate

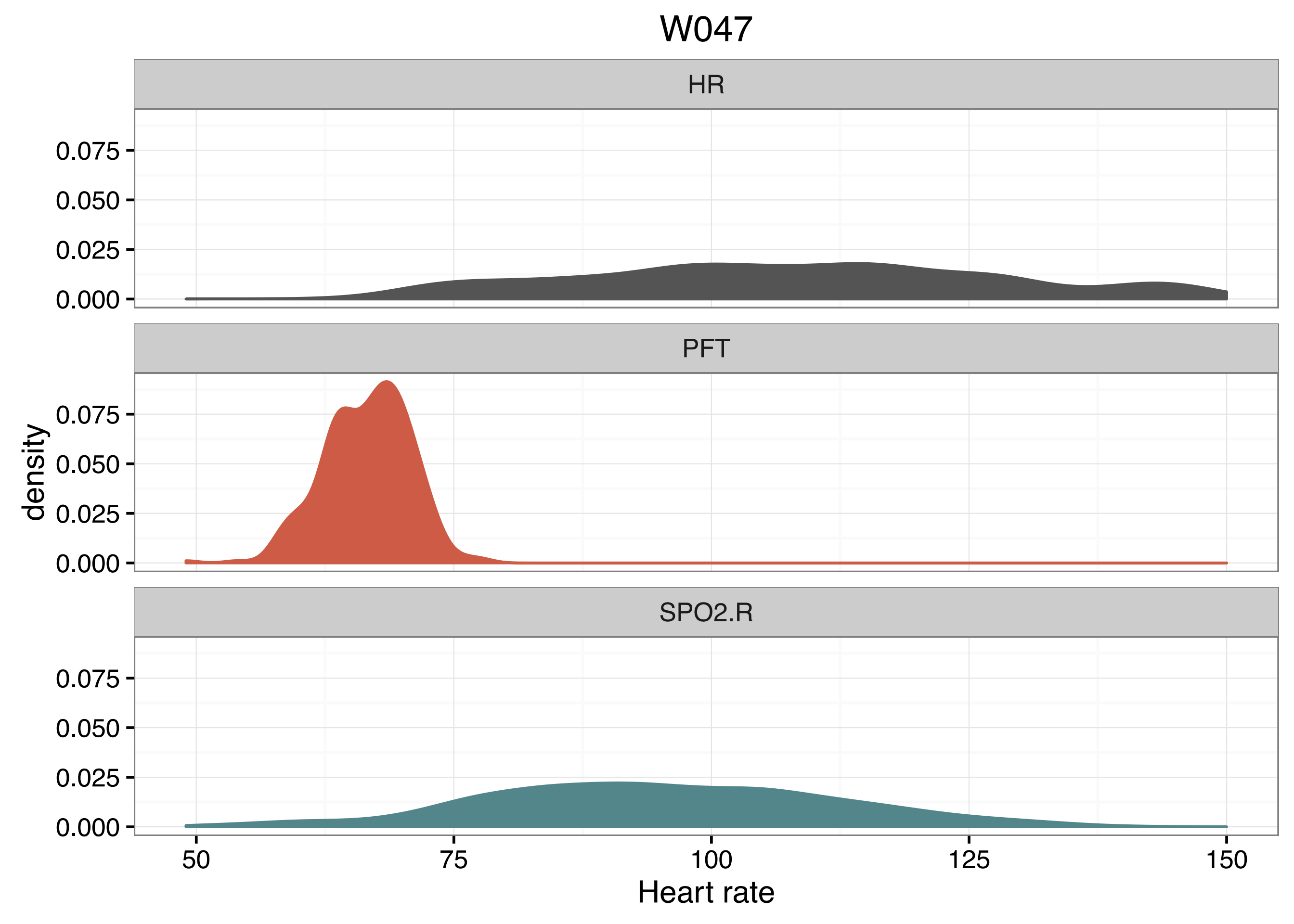
W047
HR
PFT
SPO2.R
density
0.075
0.050
0.025
0.000
50
75
100
125
150
Heart rate

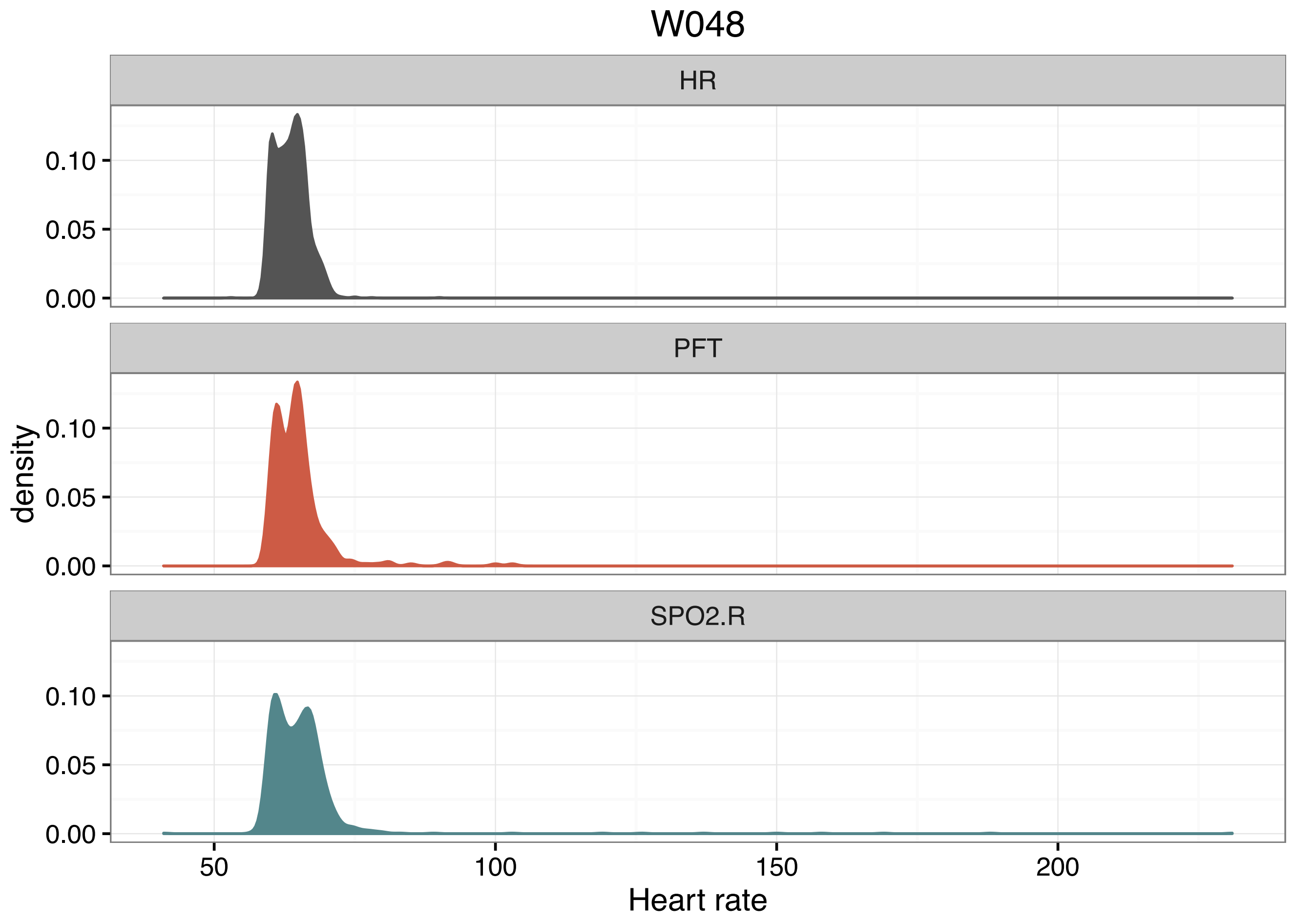
W048
HR
PFT
SPO2.R
density
0.10
0.05
0.00
50
100
150
200
Heart rate

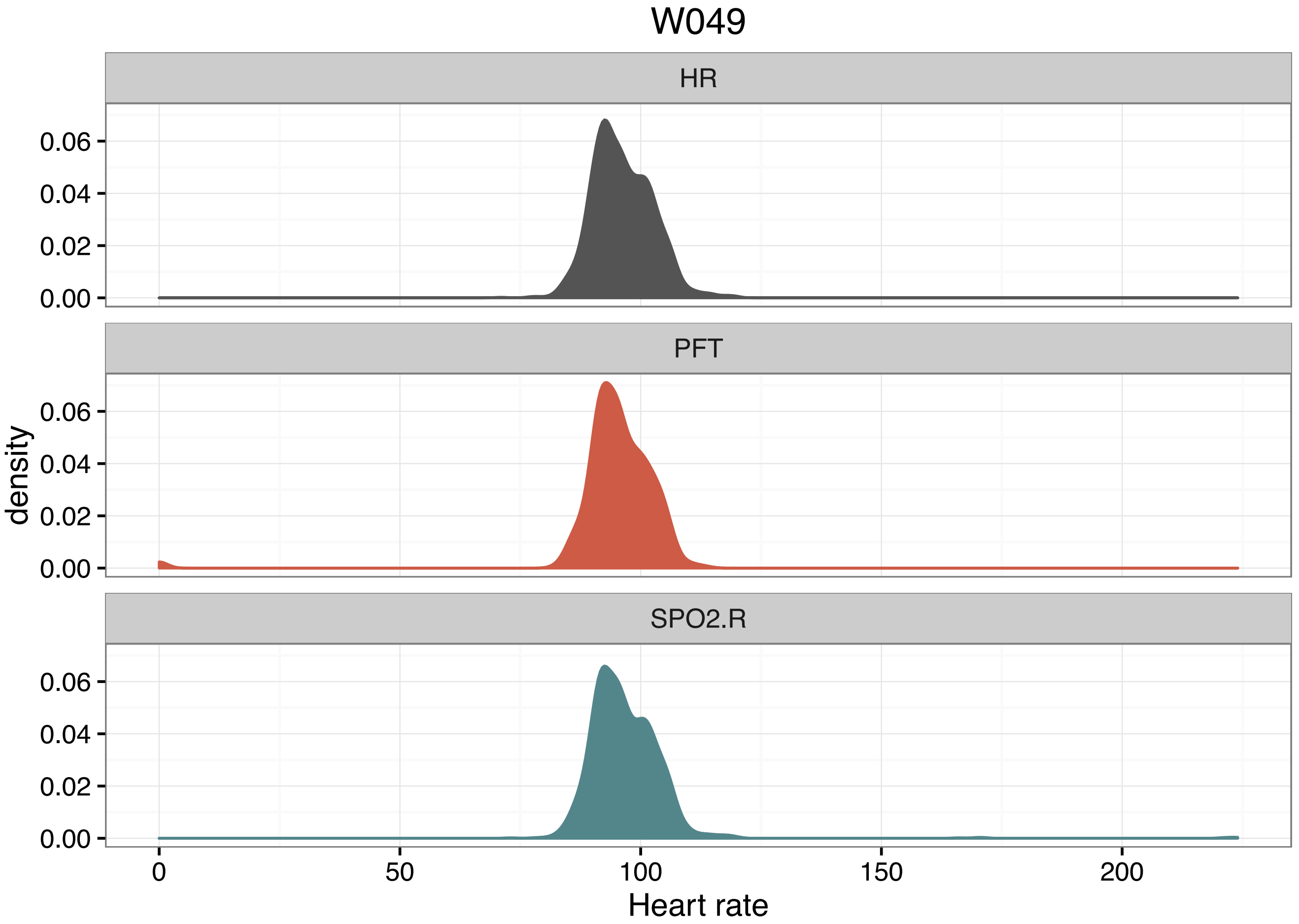

W049
HR
PFT
SPO2.R
density
0.06
0.04
0.02
0.00
0
50
100
150
200
Heart rate

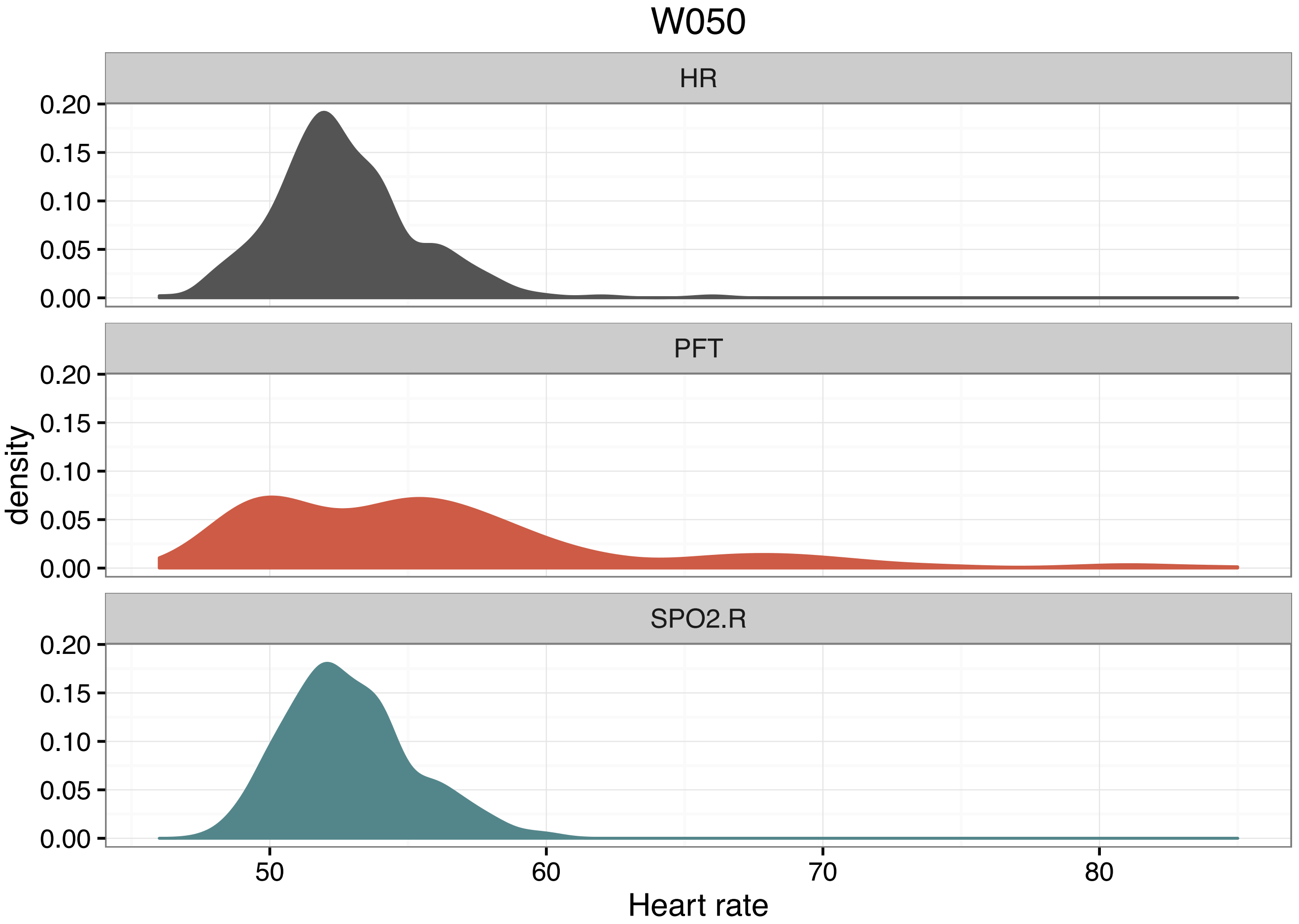
W050
HR
PFT
SPO2.R
density
0.20
0.15
0.10
0.05
0.00
50
60
70
80
Heart rate

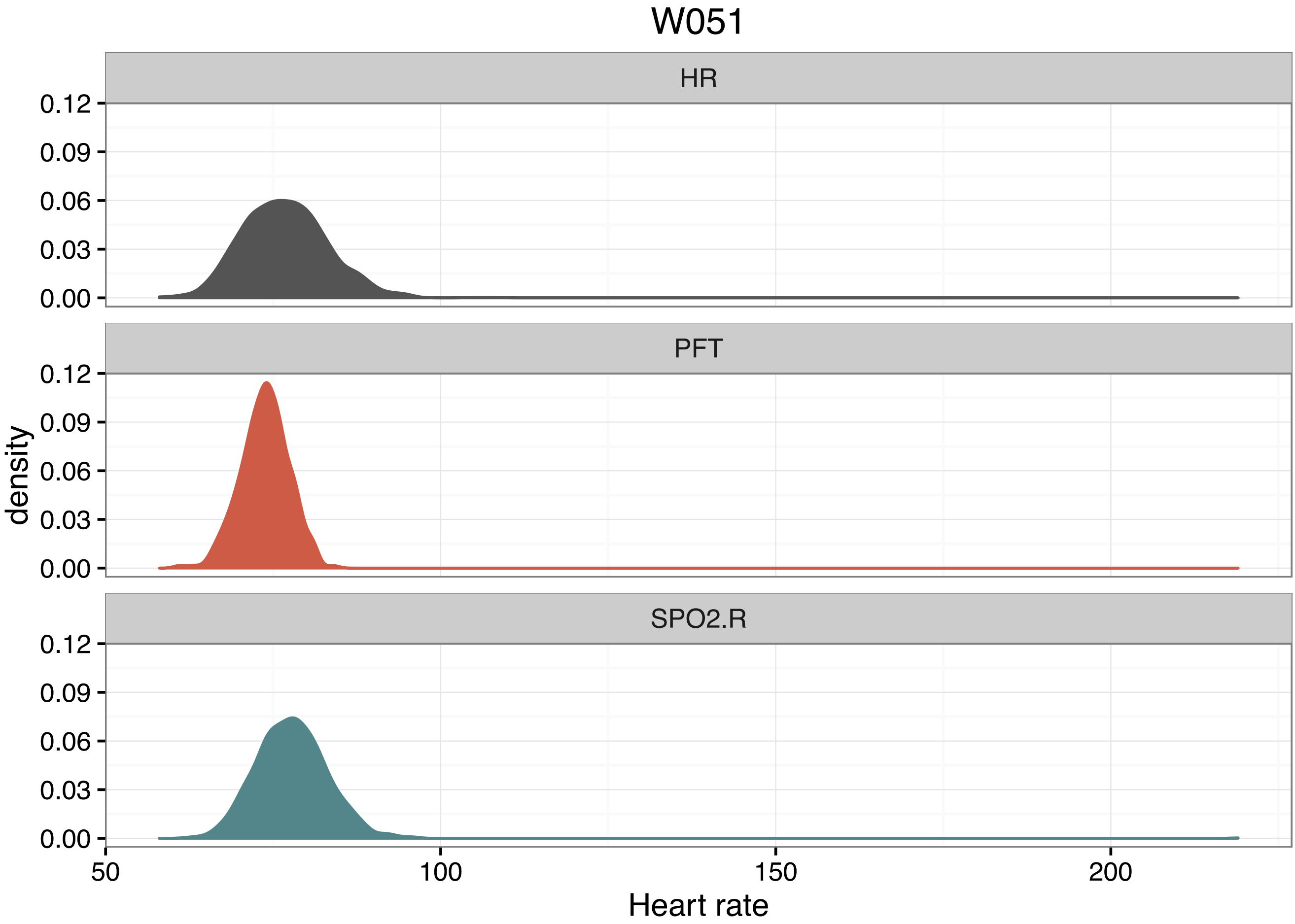
W051
HR
PFT
SPO2.R
density
0.12
0.09
0.06
0.03
0.00
50
100
150
200
Heart rate

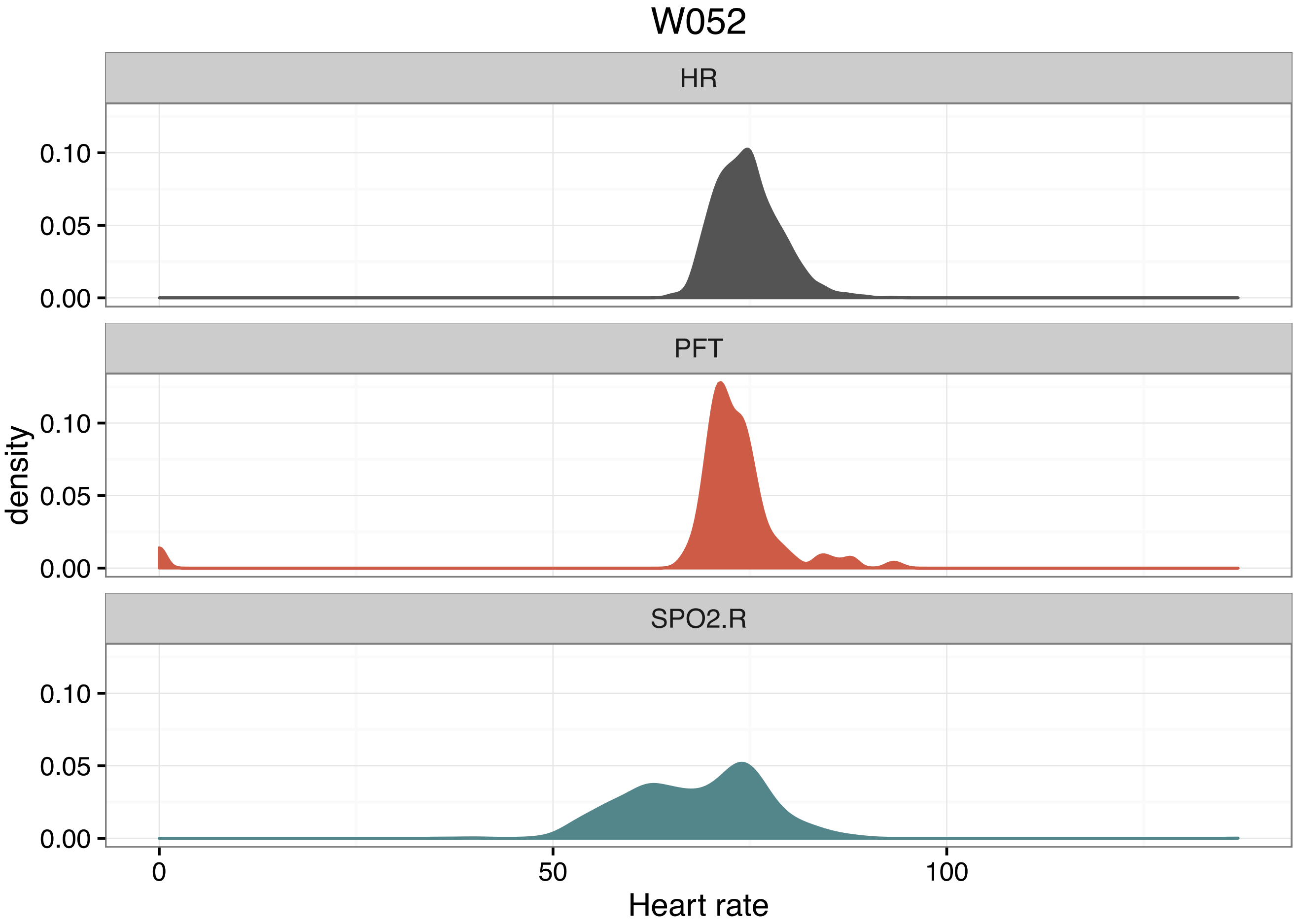
W052
HR
PFT
SPO2.R
density
0.10
0.05
0.00
0
50
100
Heart rate
