# Supplementary material for: Accuracy of a Wrist-Worn Wearable Device for Monitoring Heart Rates in Hospital Inpatients: A Prospective Observational Study
Source: J Med Internet Res. 2016 Sep 20;18(9):e253. doi: 10.2196/jmir.6025 (PMC5050383; doi:10.2196/jmir.6025)
Supplement: Multimedia Appendix 2 [file jmir_v18i9e253_app2.pdf]

Supplementary Table 2. Results from the pooled analysis of heart rate pairs. N.B. zeros in PFT = missing data

	**PFT**	**SPO2.R**
Pairs (n)	12360	56394
Median difference (bpm)	1	0
IQR (bpm)	5	2
Correlation coefficient	0.74	0.91
Wilcox p-value	2.2E-16	0.1837
Bland Altman analysis		
Mean difference (bpm)	-4.7 (95% CI-4.91 to -4.44)	-0.2 (95% CI -0.30 to -0.16)
Lower limit of agreement (bpm)	-31 (95% CI -31.22 to -30.40)	-17 (95% CI -17.01 to -16.77)
Upper limit of agreement (bpm)	21 (95% CI 21.06 to 21.87)	16 (95% CI 16.31 to 16.55)
