# Supplementary figures and images for: Accuracy of a Wrist-Worn Wearable Device for Monitoring Heart Rates in Hospital Inpatients: A Prospective Observational Study
Source: J Med Internet Res. 2016 Sep 20;18(9):e253. doi: 10.2196/jmir.6025 (PMC5050383; doi:10.2196/jmir.6025)

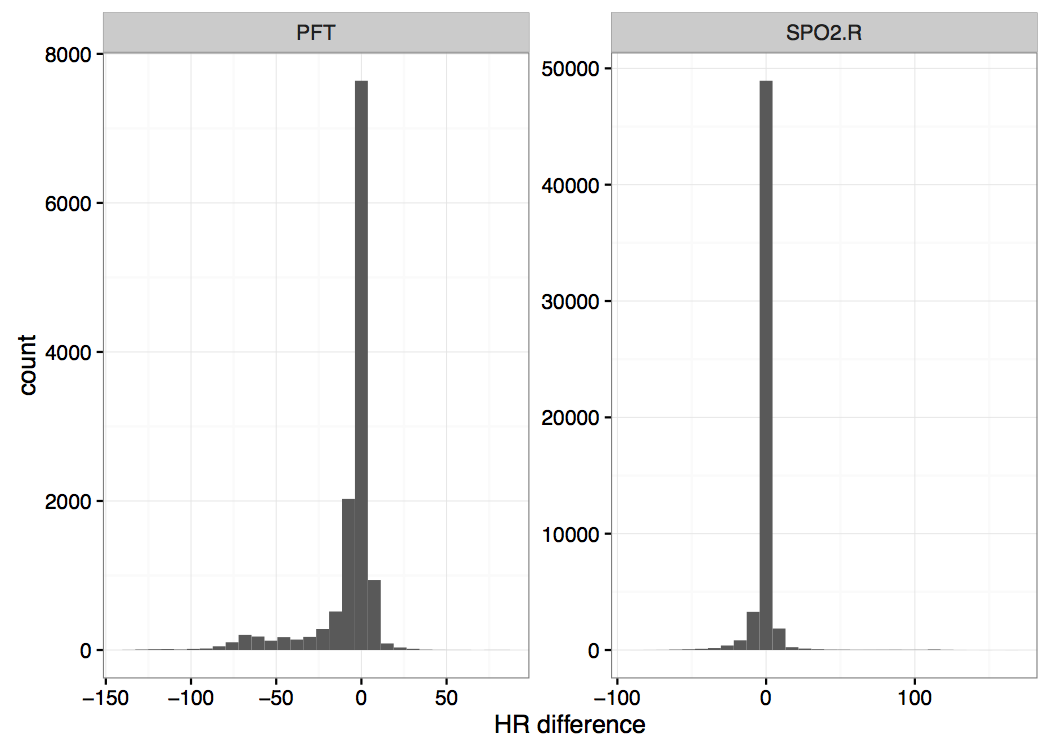

Supplement: Multimedia Appendix 3 [file jmir_v18i9e253_app3.png]
