# Supplementary material for: Accuracy of a Wrist-Worn Wearable Device for Monitoring Heart Rates in Hospital Inpatients: A Prospective Observational Study
Source: J Med Internet Res. 2016 Sep 20;18(9):e253. doi: 10.2196/jmir.6025 (PMC5050383; doi:10.2196/jmir.6025)
Supplement: Multimedia Appendix 4 [file jmir_v18i9e253_app4.pdf]

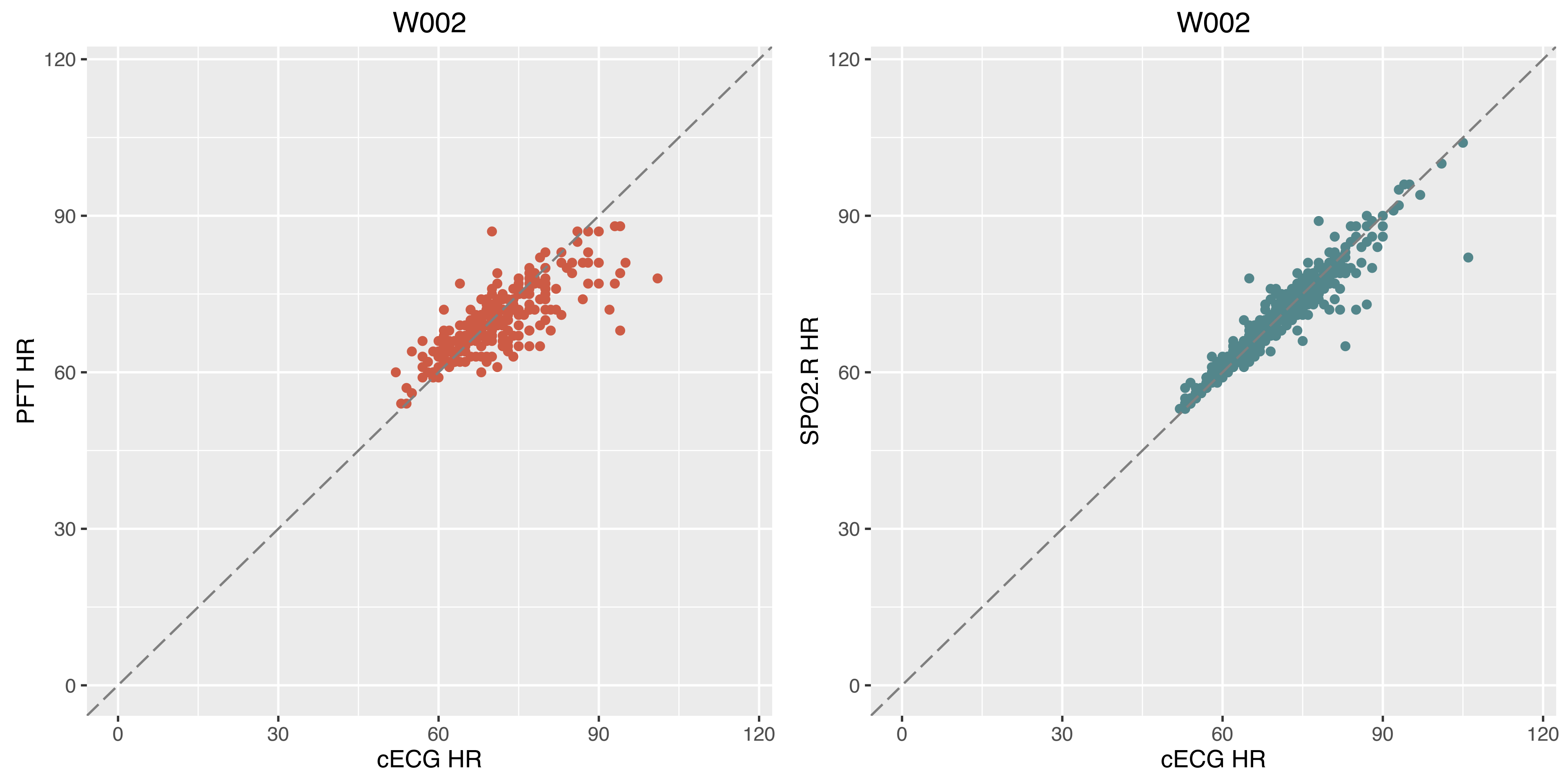
W002
PFT HR
cECG HR
0
30
60
90
120
W002
SPO2.R HR
cECG HR
0
30
60
90
120

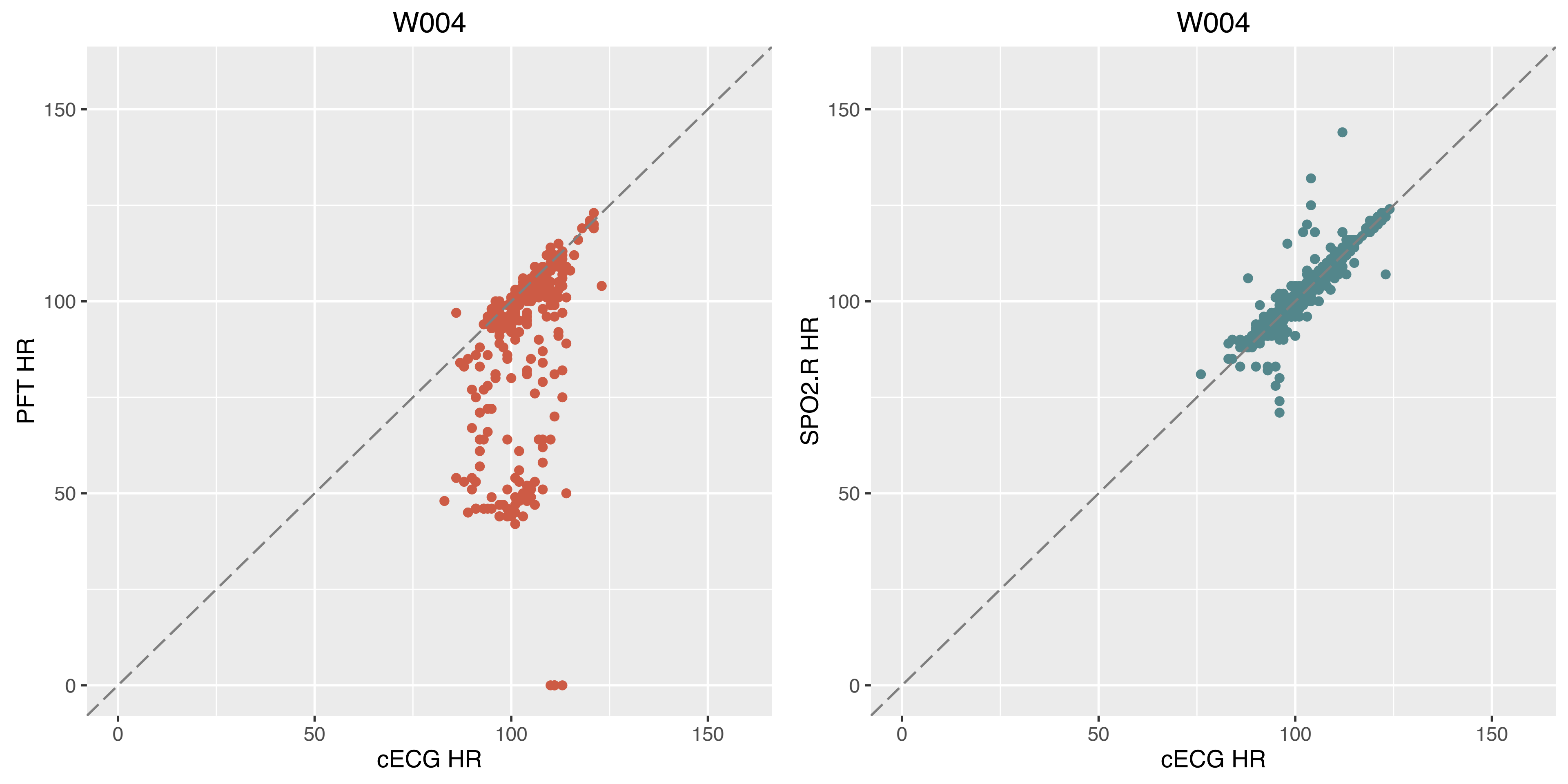
W004
PFT HR
cECG HR
0
50
100
150
W004
SPO2.R HR
cECG HR
0
50
100
150

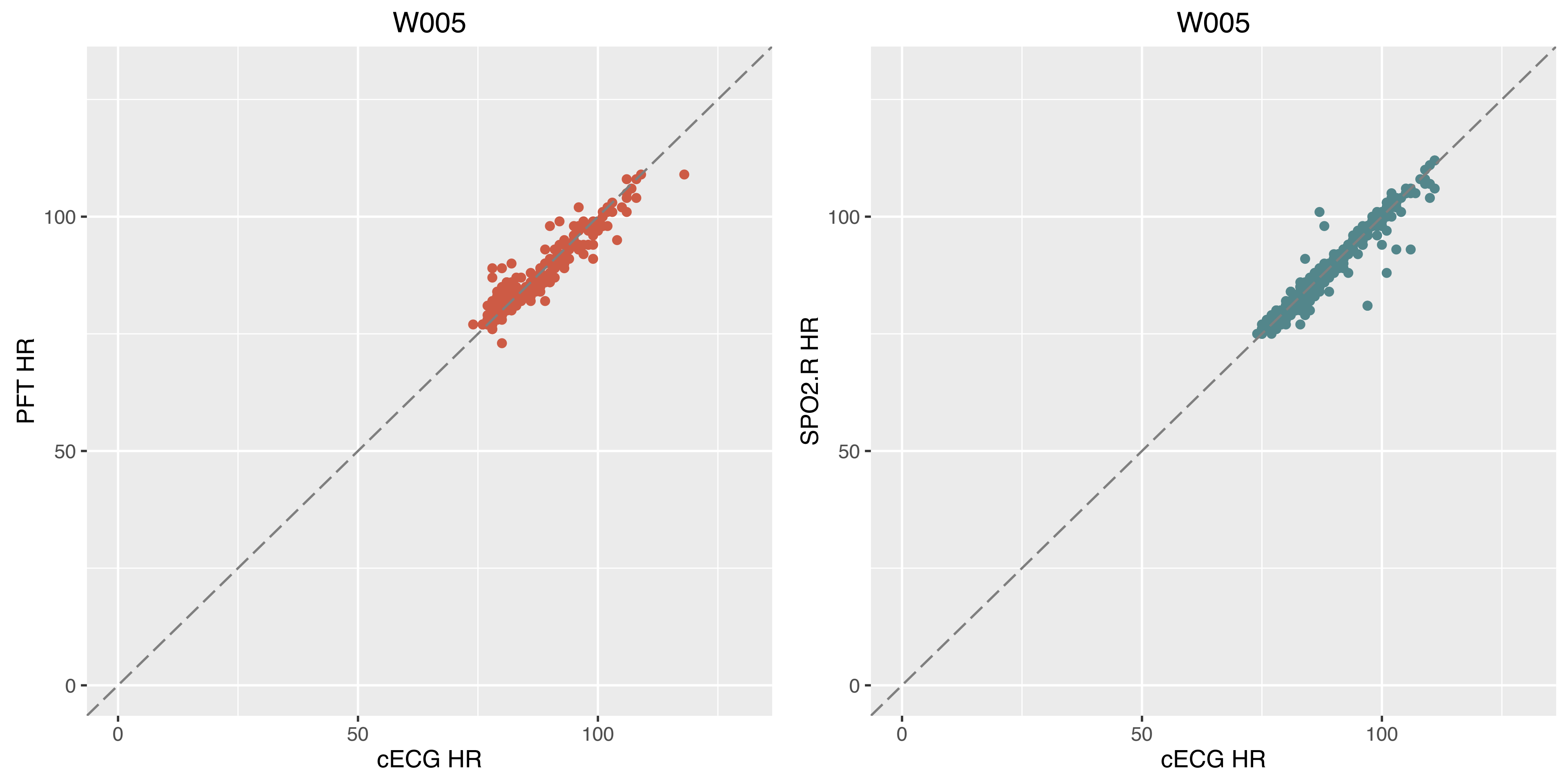

W005
PFT HR
cECG HR
100
50
0
0
50
100
W005
SPO2.R HR
cECG HR
100
50
0
0
50
100

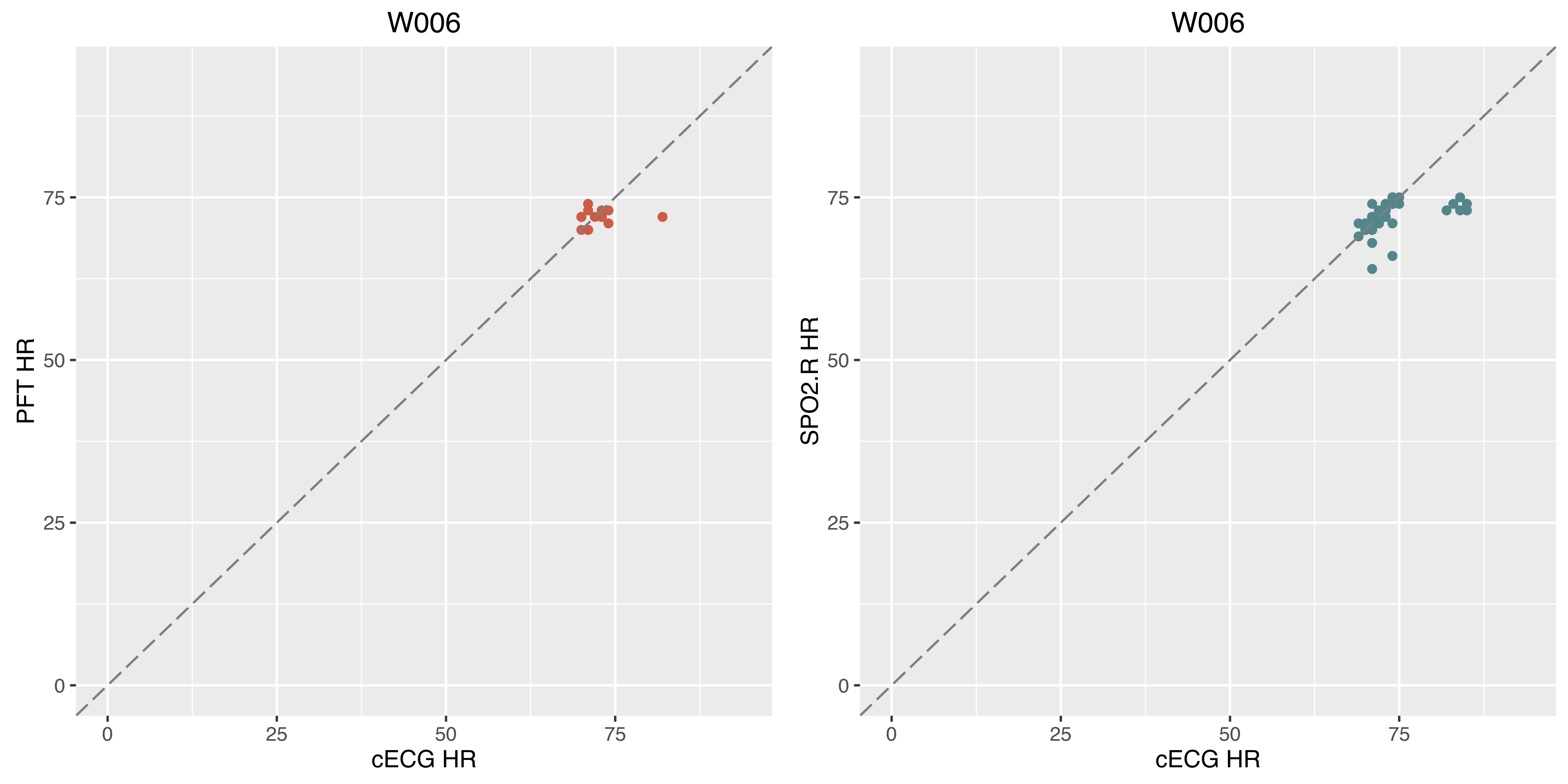
W006
PFT HR
cECG HR
0
25
50
75
W006
SPO2.R HR
cECG HR

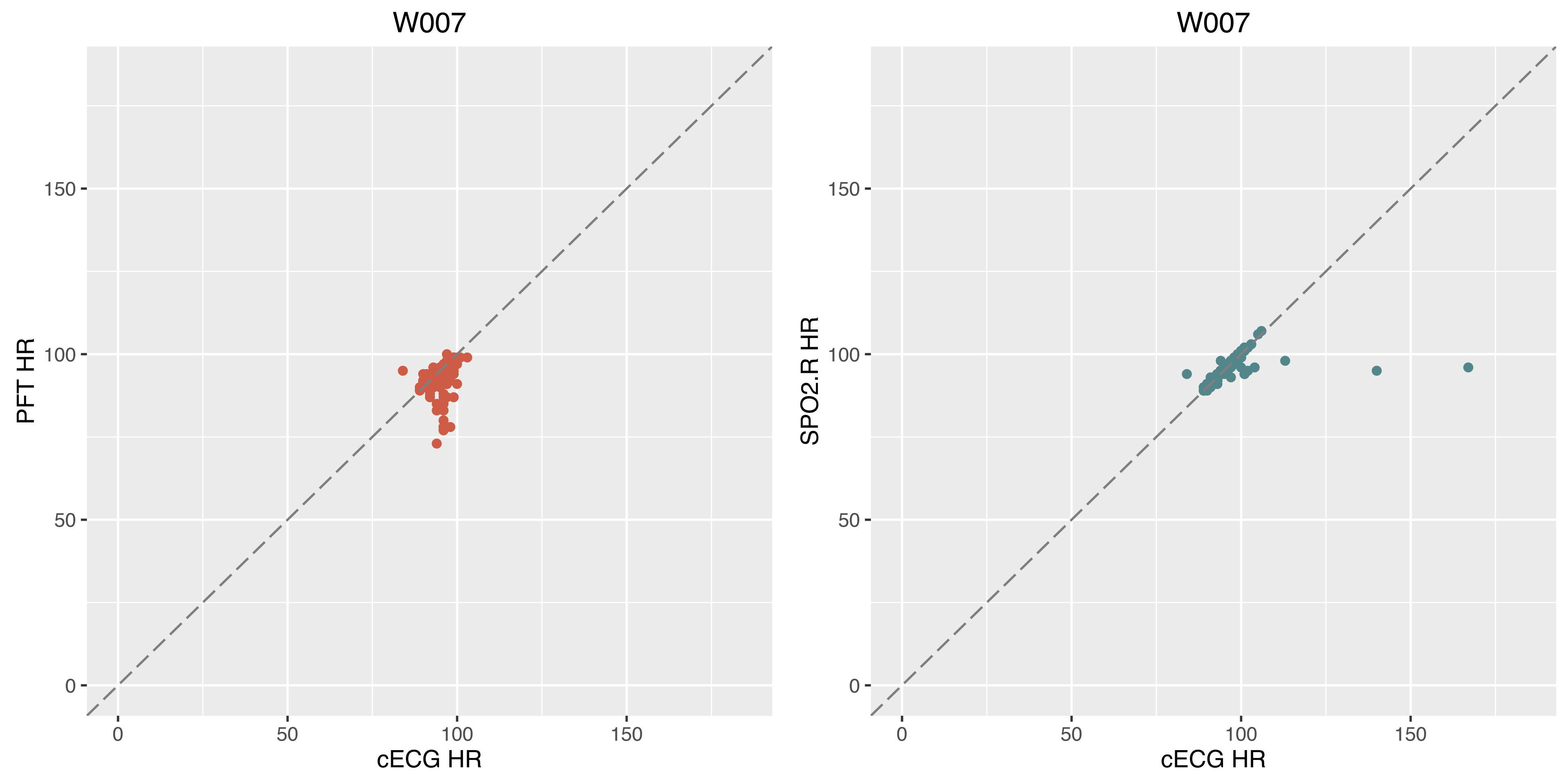
W007
PFT HR
cECG HR
150
100
50
0
0
50
100
150
W007
SPO2.R HR
cECG HR
150
100
50
0
0
50
100
150

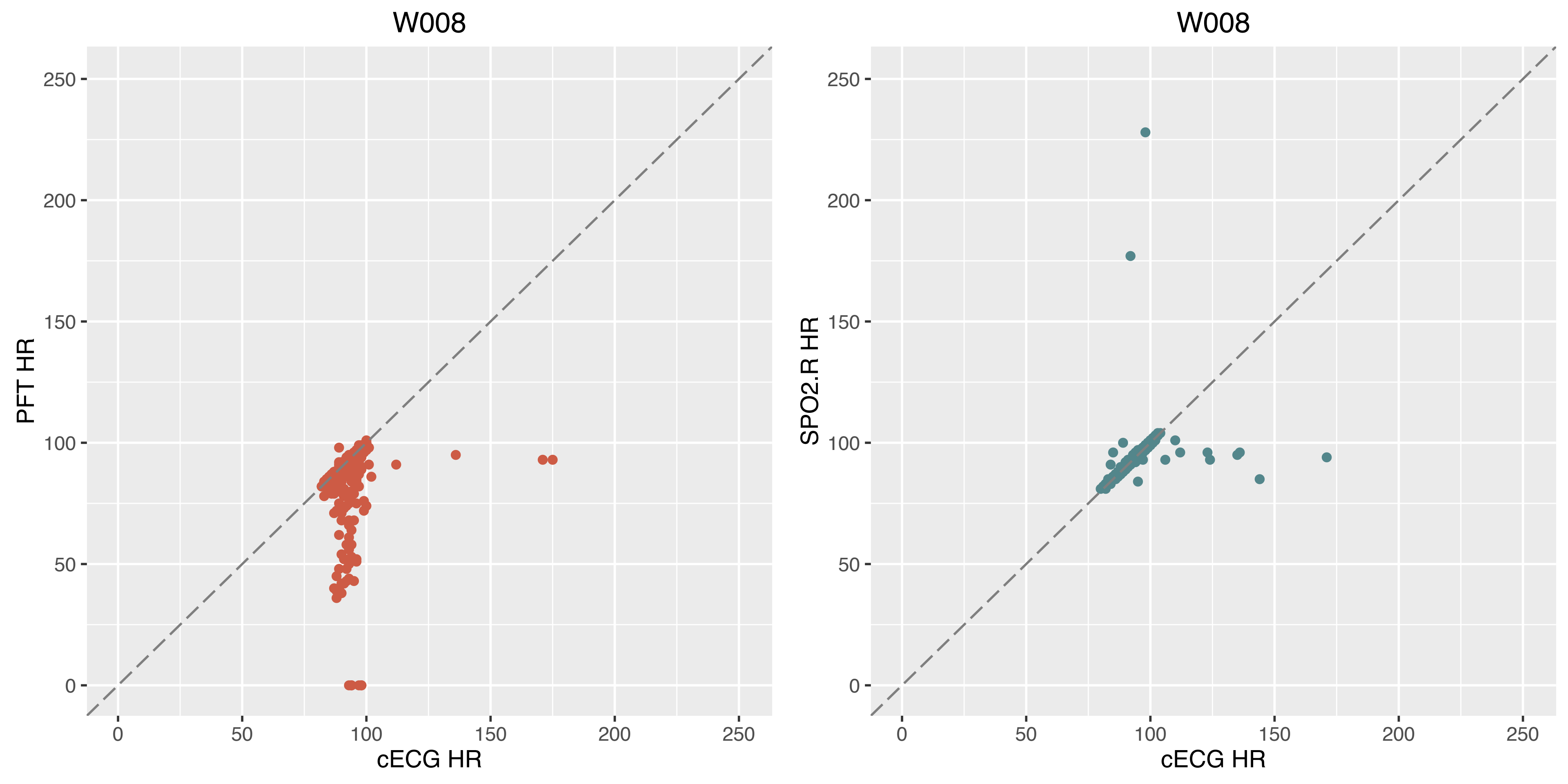
W008
PFT HR
cECG HR
W008
SPO2.R HR
cECG HR
0
50
100
150
200
250

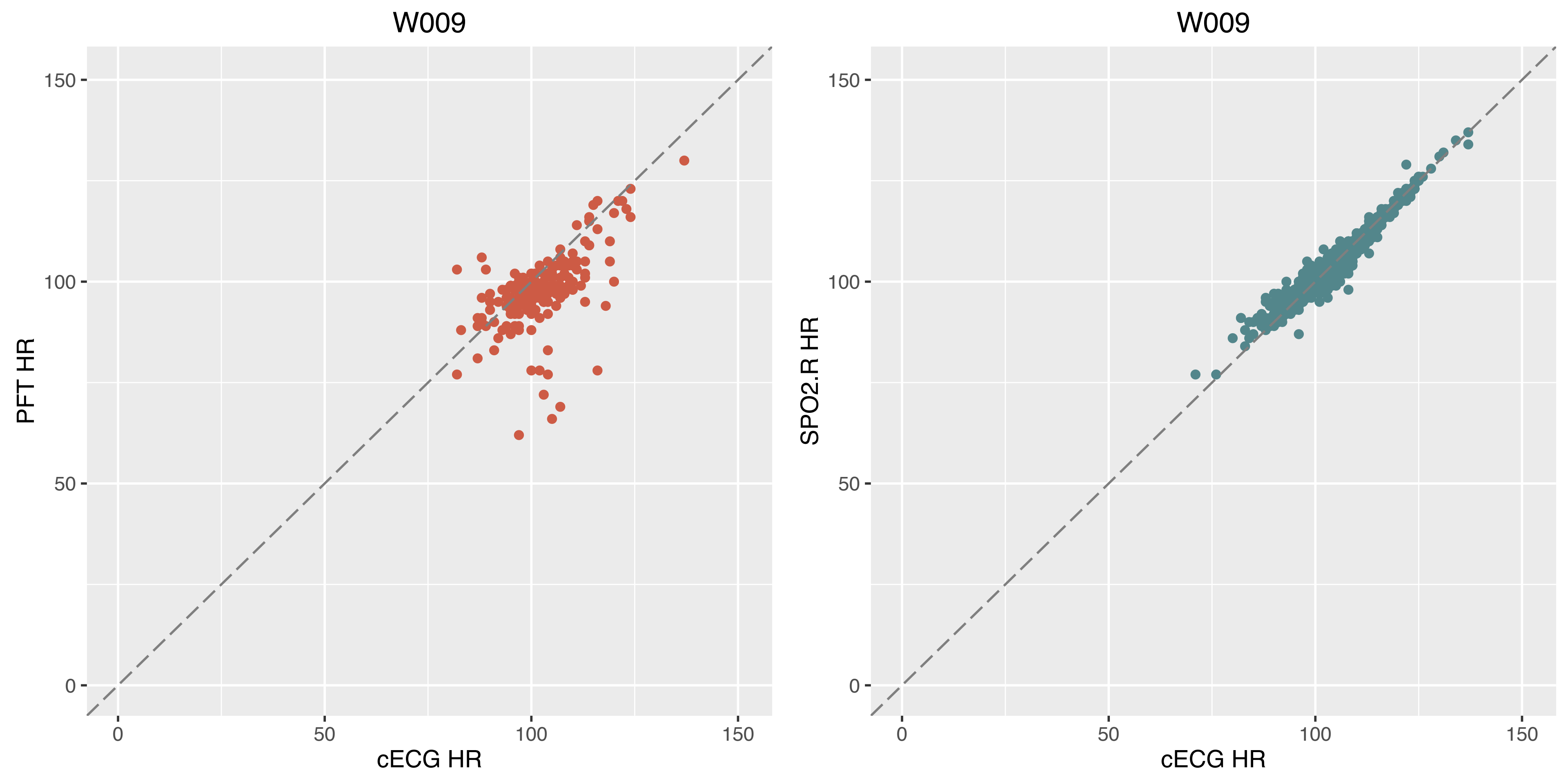

W009
PFT HR
cECG HR
W009
SPO2.R HR
cECG HR
0
50
100
150

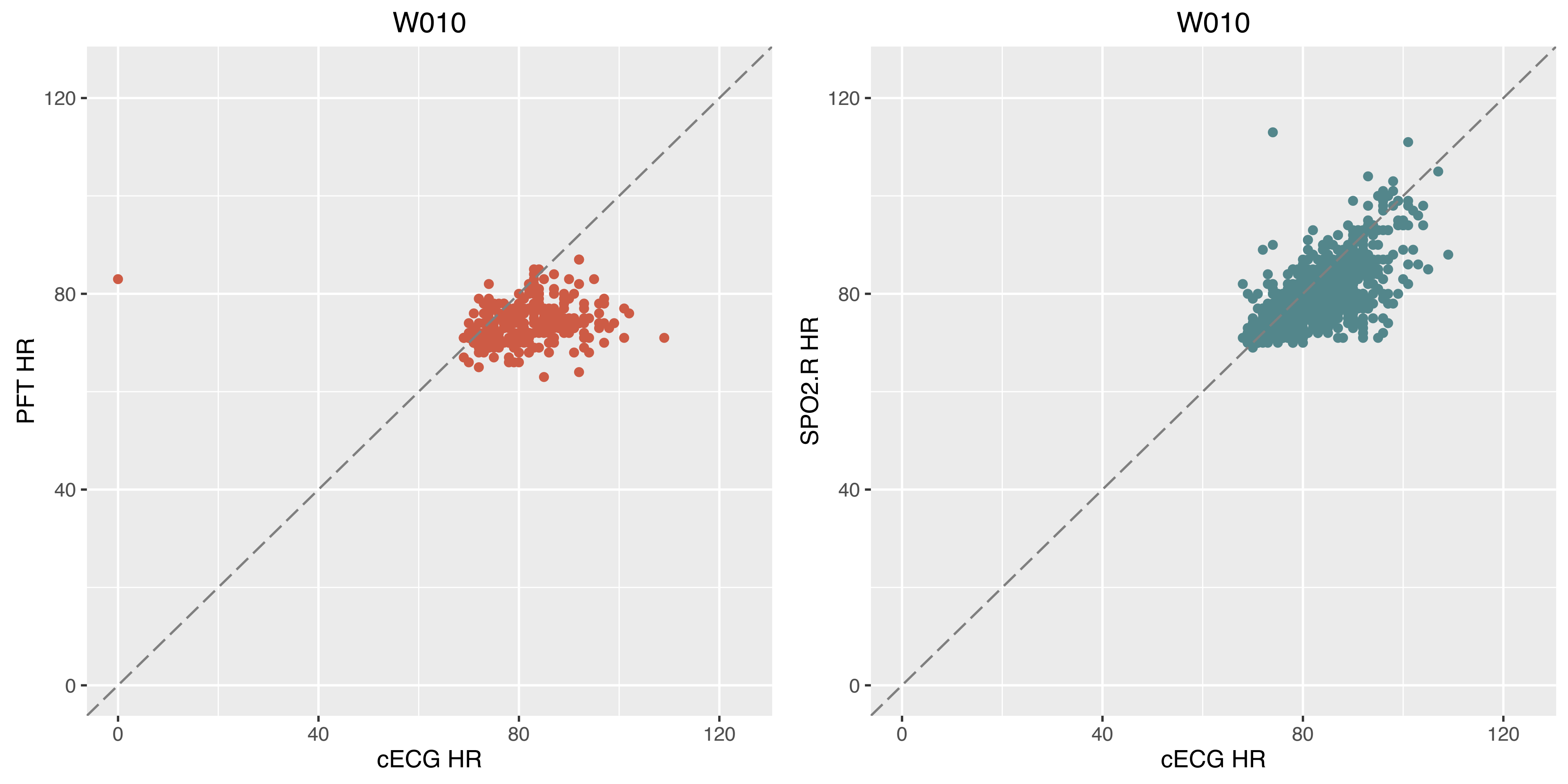
W010
PFT HR
cECG HR
W010
SPO2.R HR
cECG HR
0
40
80
120

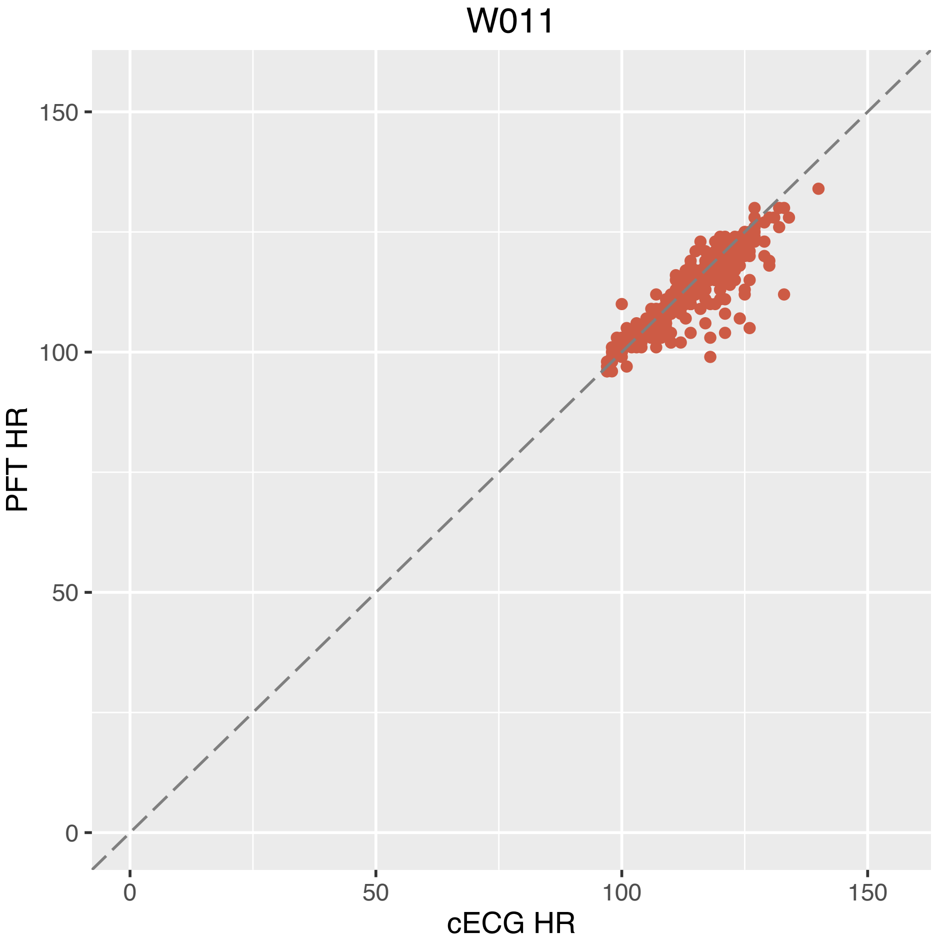

W011
PFT HR
cECG HR
150
100
50
0
0
50
100
150

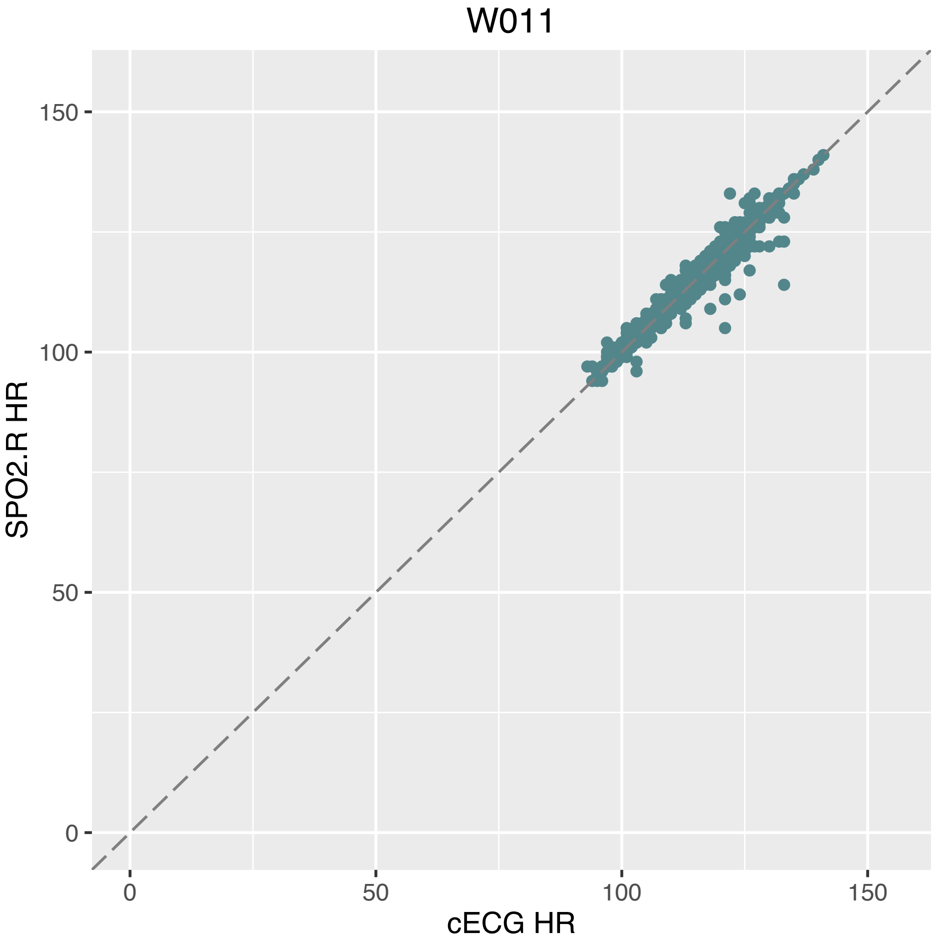

W011
SPO2.R HR
cECG HR
150
100
50
0
0
50
100
150

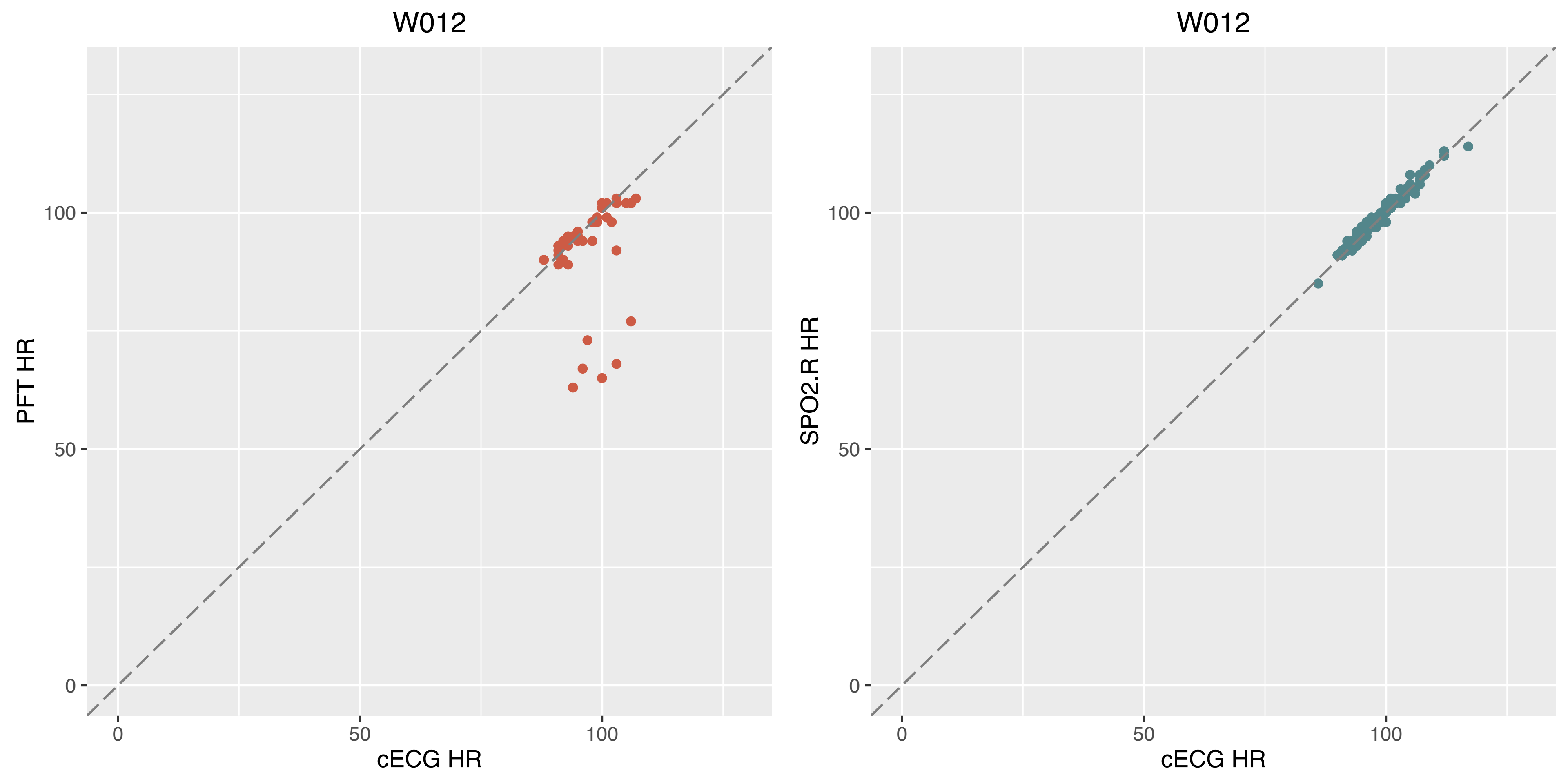

W012
PFT HR
cECG HR
0
50
100
W012
SPO2.R HR
cECG HR
0
50
100

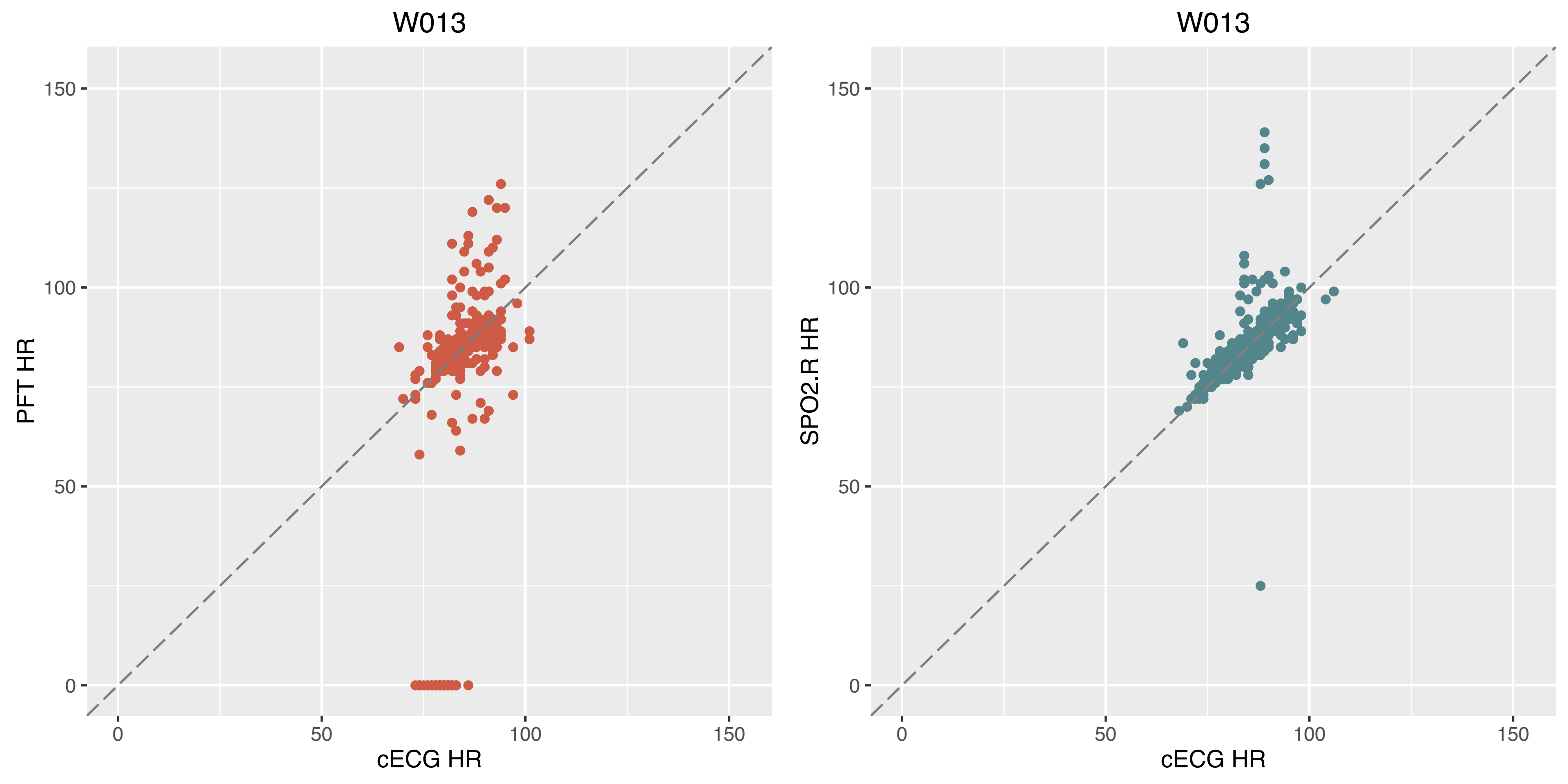
W013
W013
PFT HR
SPO2.R HR
cECG HR
cECG HR
0
50
100
150
0
50
100
150

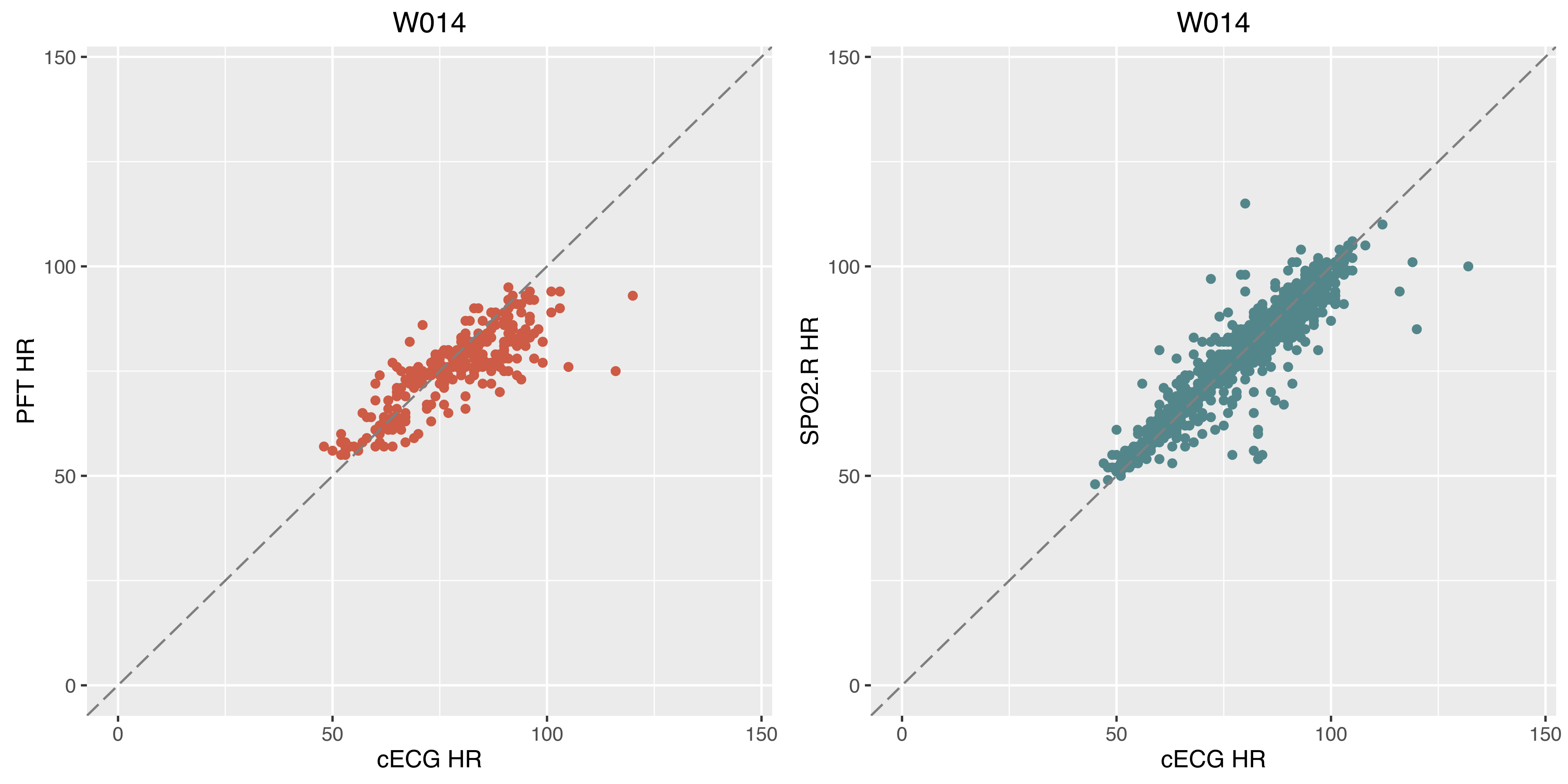

W014
PFT HR
cECG HR
0
50
100
150
W014
SPO2.R HR
cECG HR
0
50
100
150

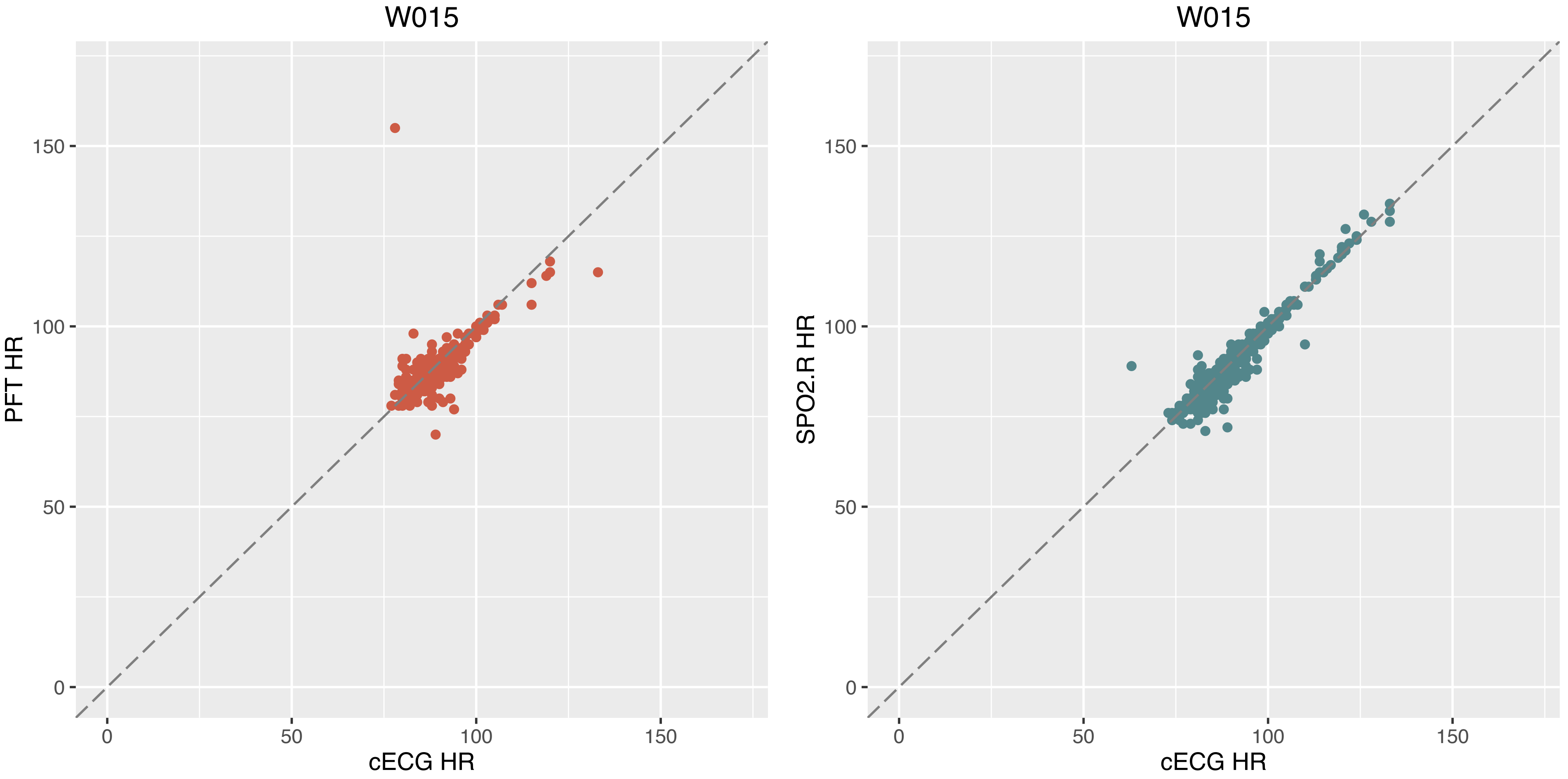
W015
PFT HR
cECG HR
150
100
50
0
0
50
100
150
W015
SPO2.R HR
cECG HR
150
100
50
0
0
50
100
150

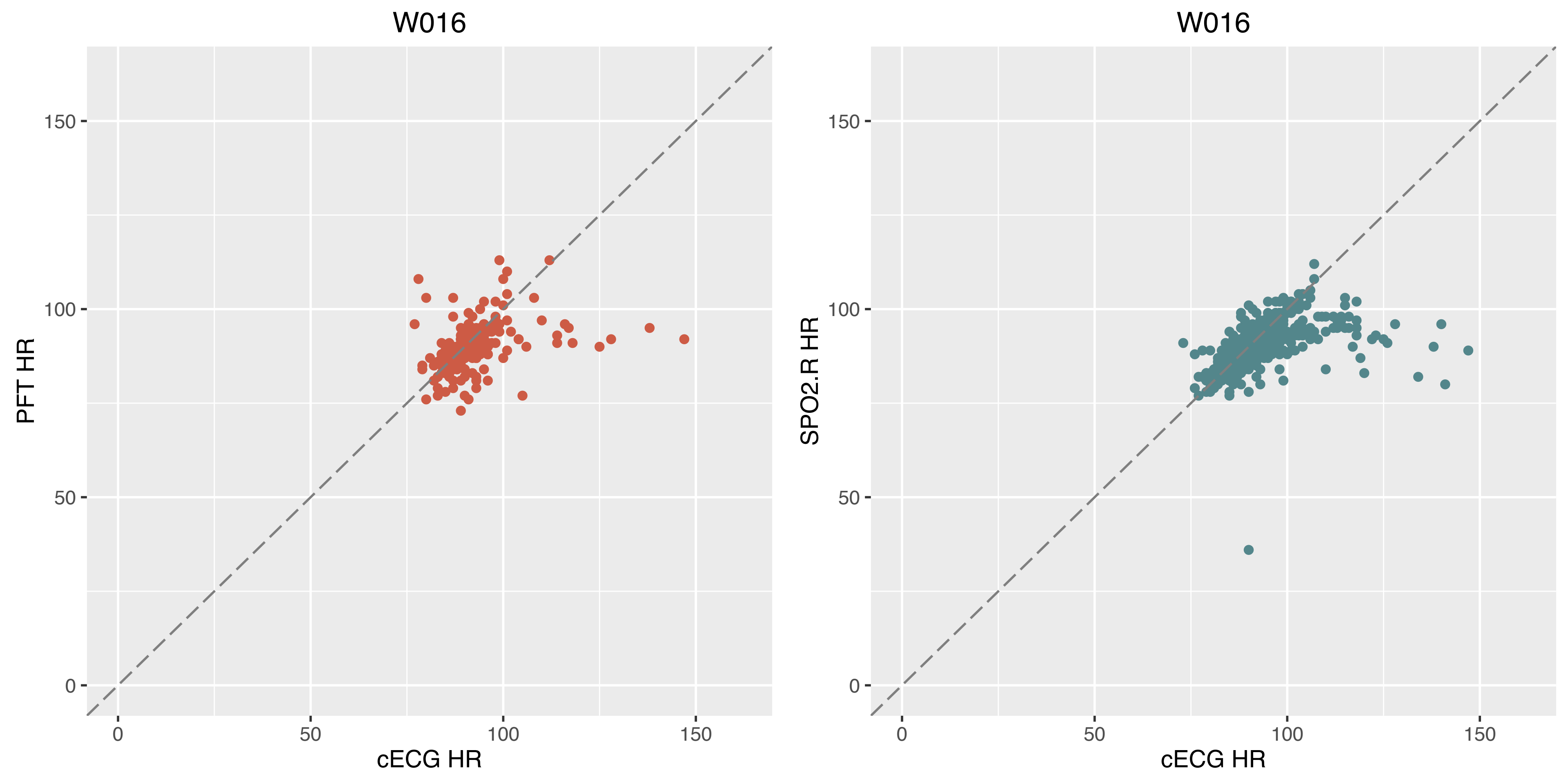
W016
PFT HR
cECG HR
150
100
50
0
W016
SPO2.R HR
cECG HR

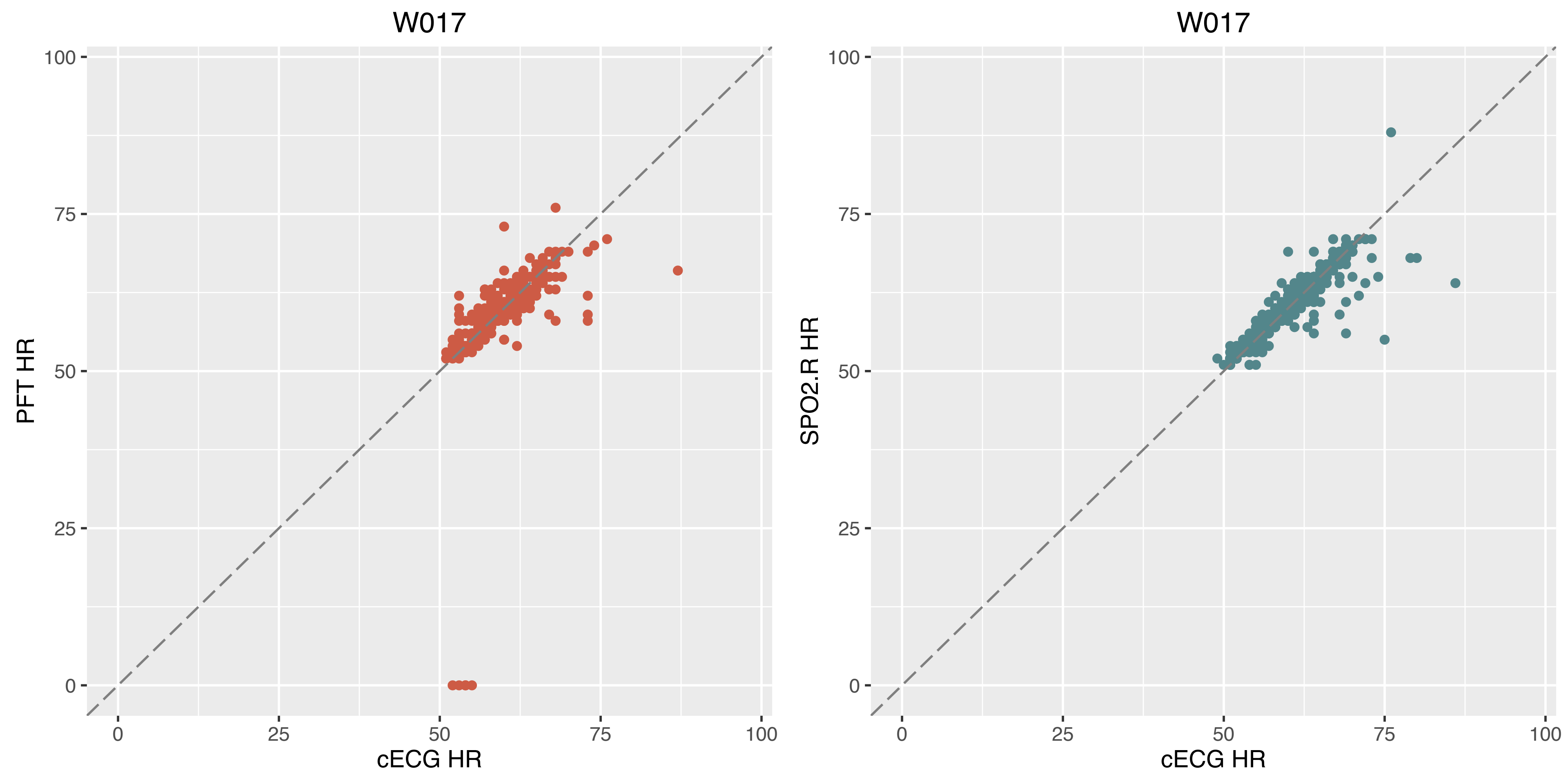

W017
PFT HR
cECG HR
W017
SPO2.R HR
cECG HR
0
25
50
75
100

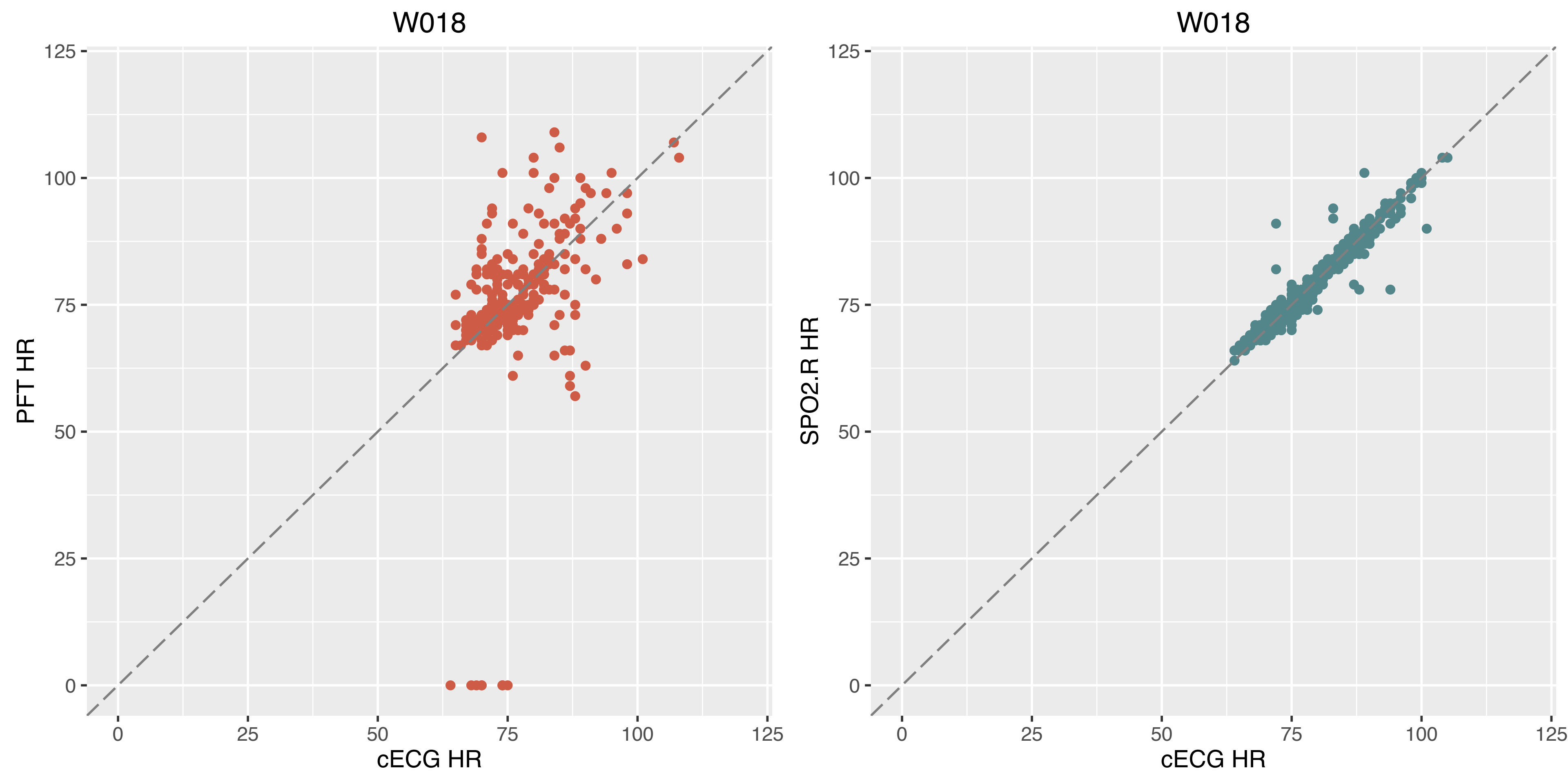

W018
PFT HR
cECG HR
W018
SPO2.R HR
cECG HR
0
25
50
75
100
125

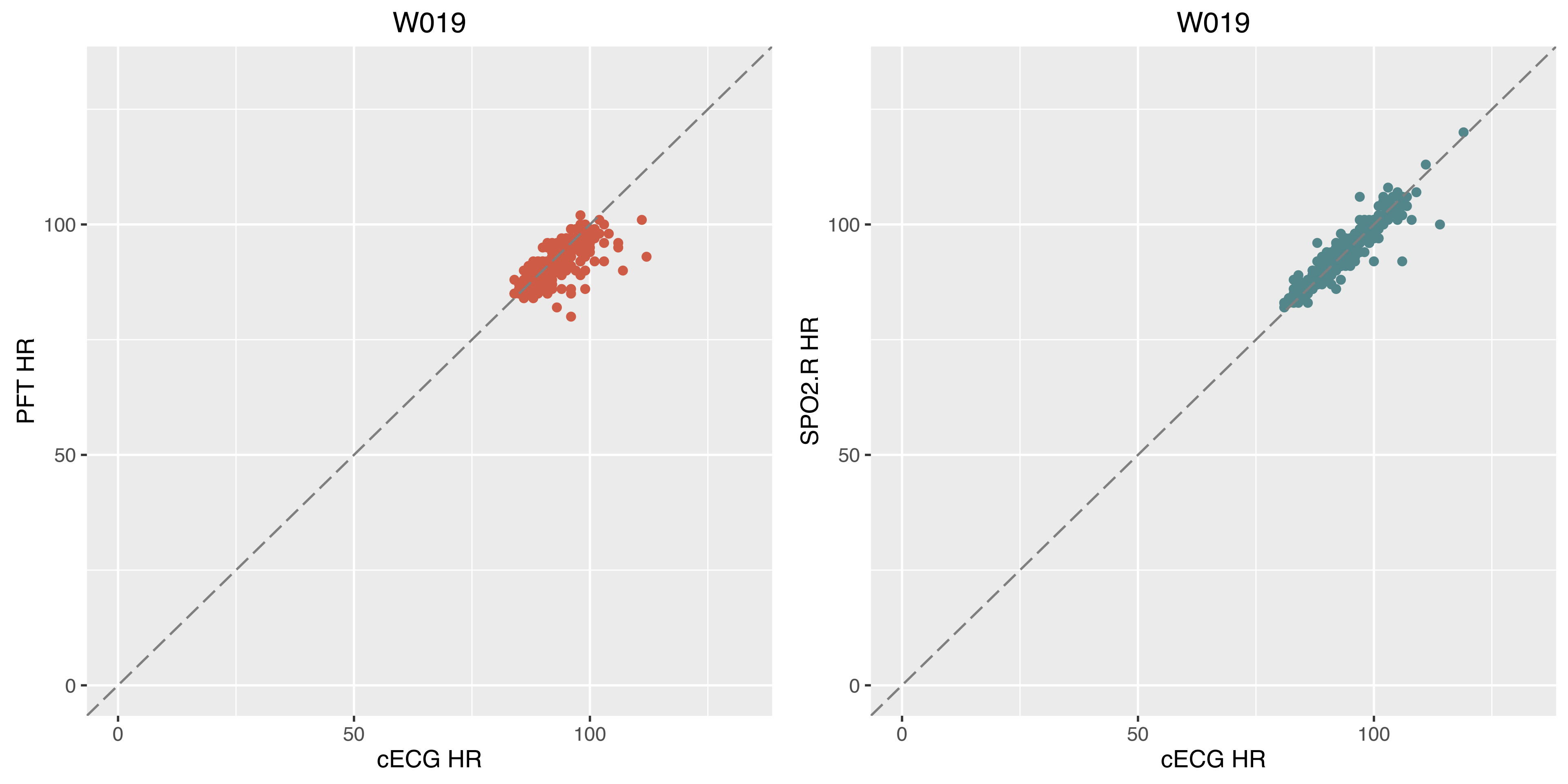

W019
PFT HR
cECG HR
0
50
100
W019
SPO2.R HR
cECG HR
0
50
100

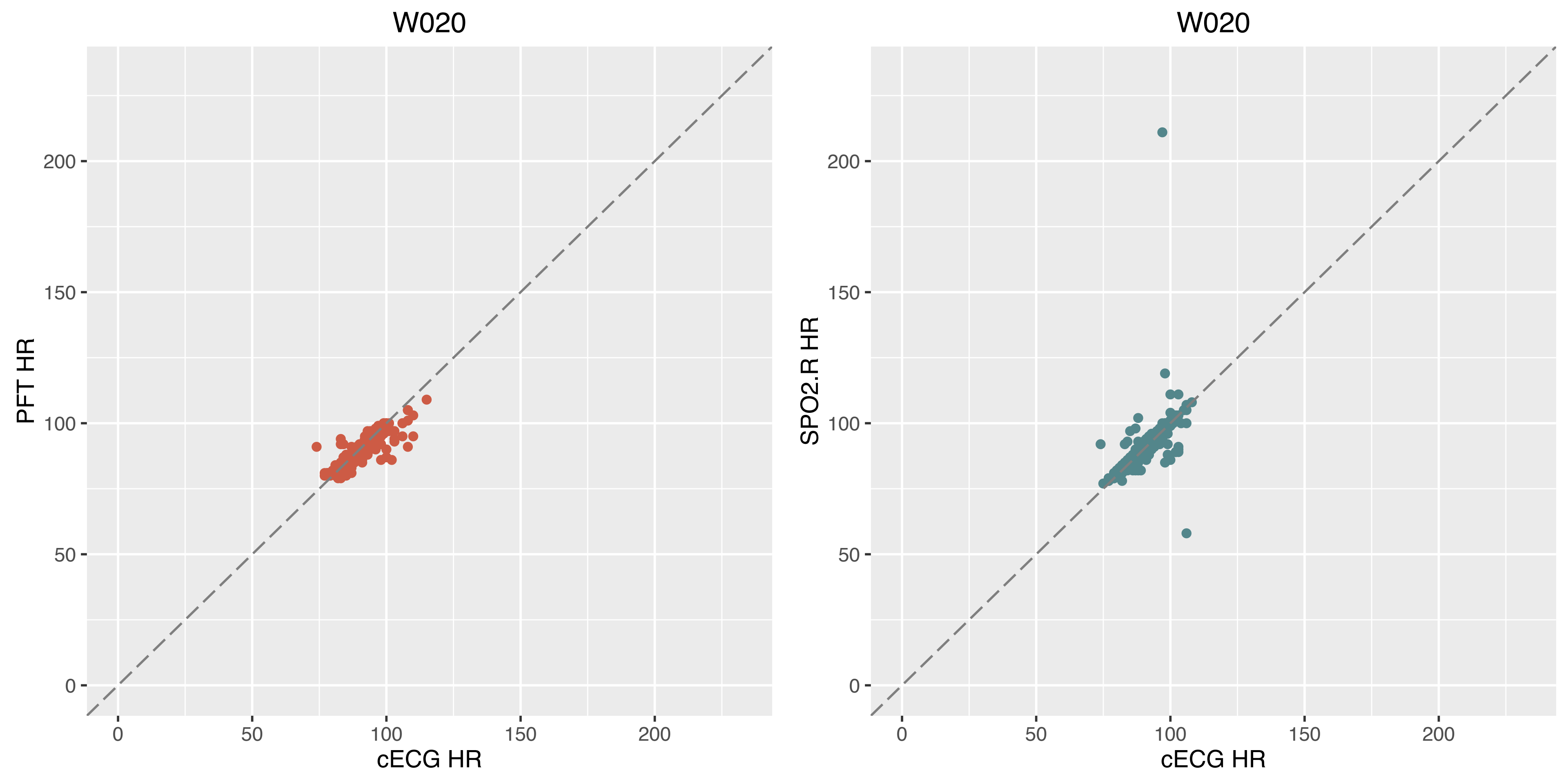
W020
PFT HR
cECG HR
0
50
100
150
200
W020
SPO2.R HR
cECG HR

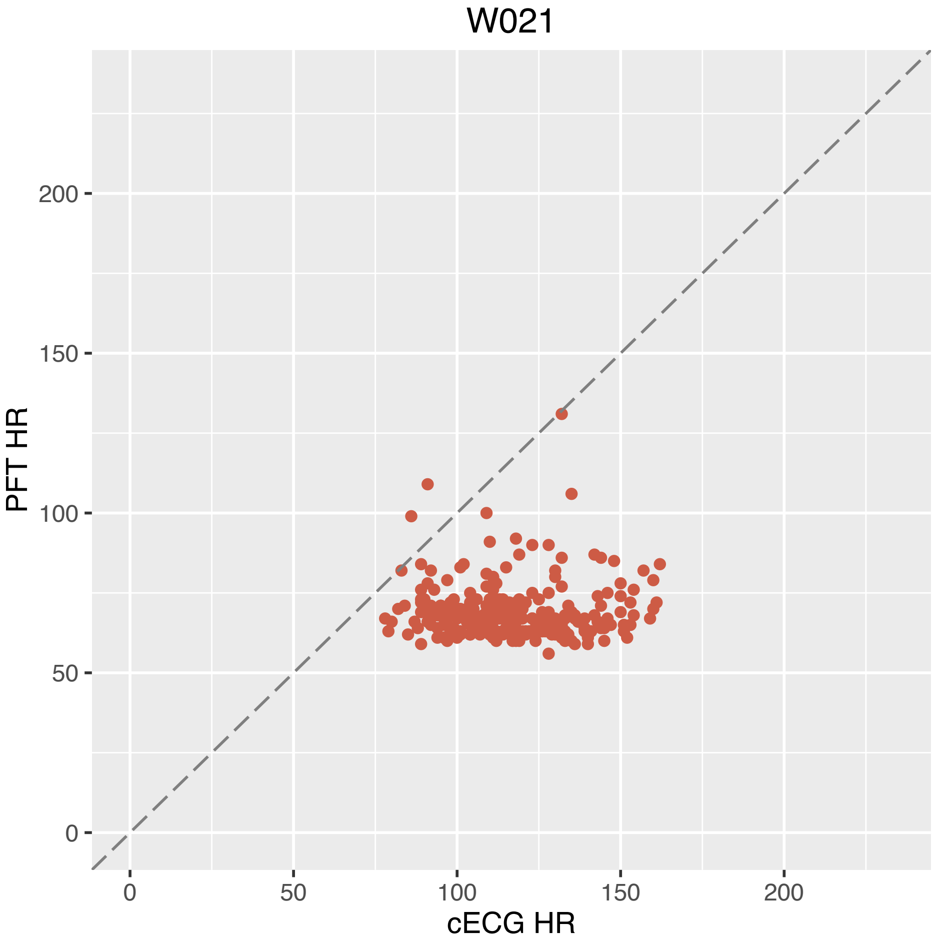

W021
PFT HR
cECG HR
0
50
100
150
200

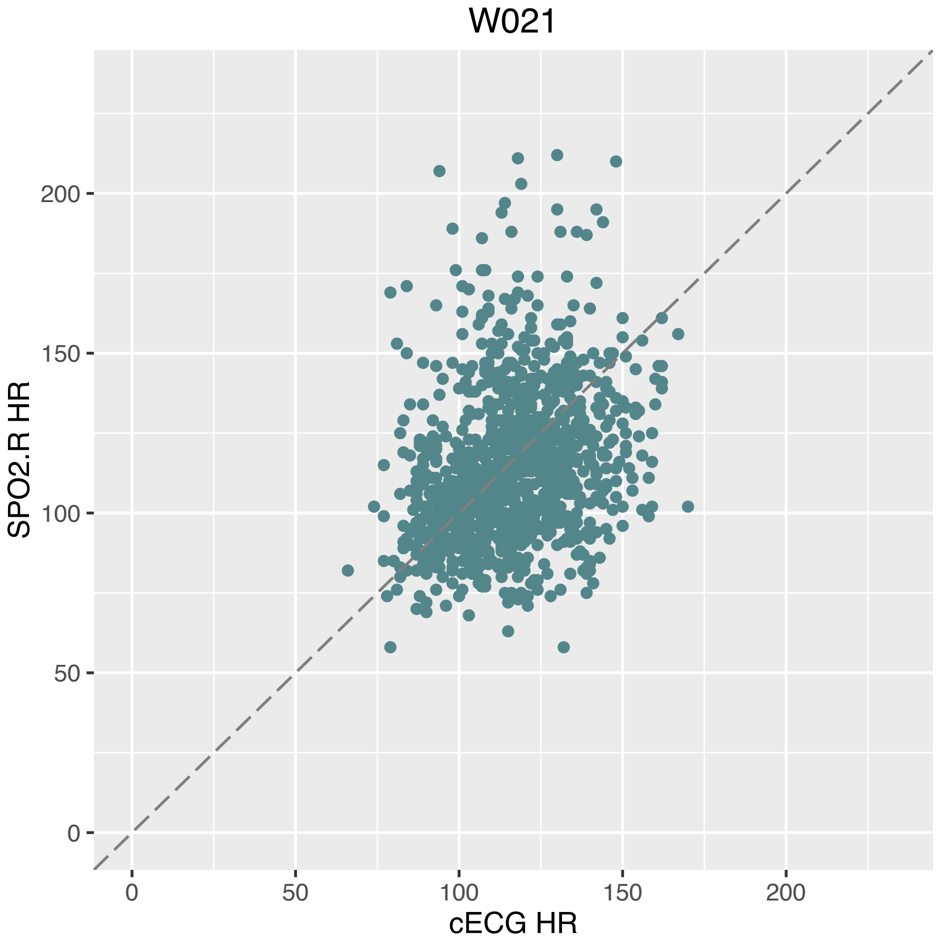

W021
SPO2.R HR
cECG HR
0
50
100
150
200

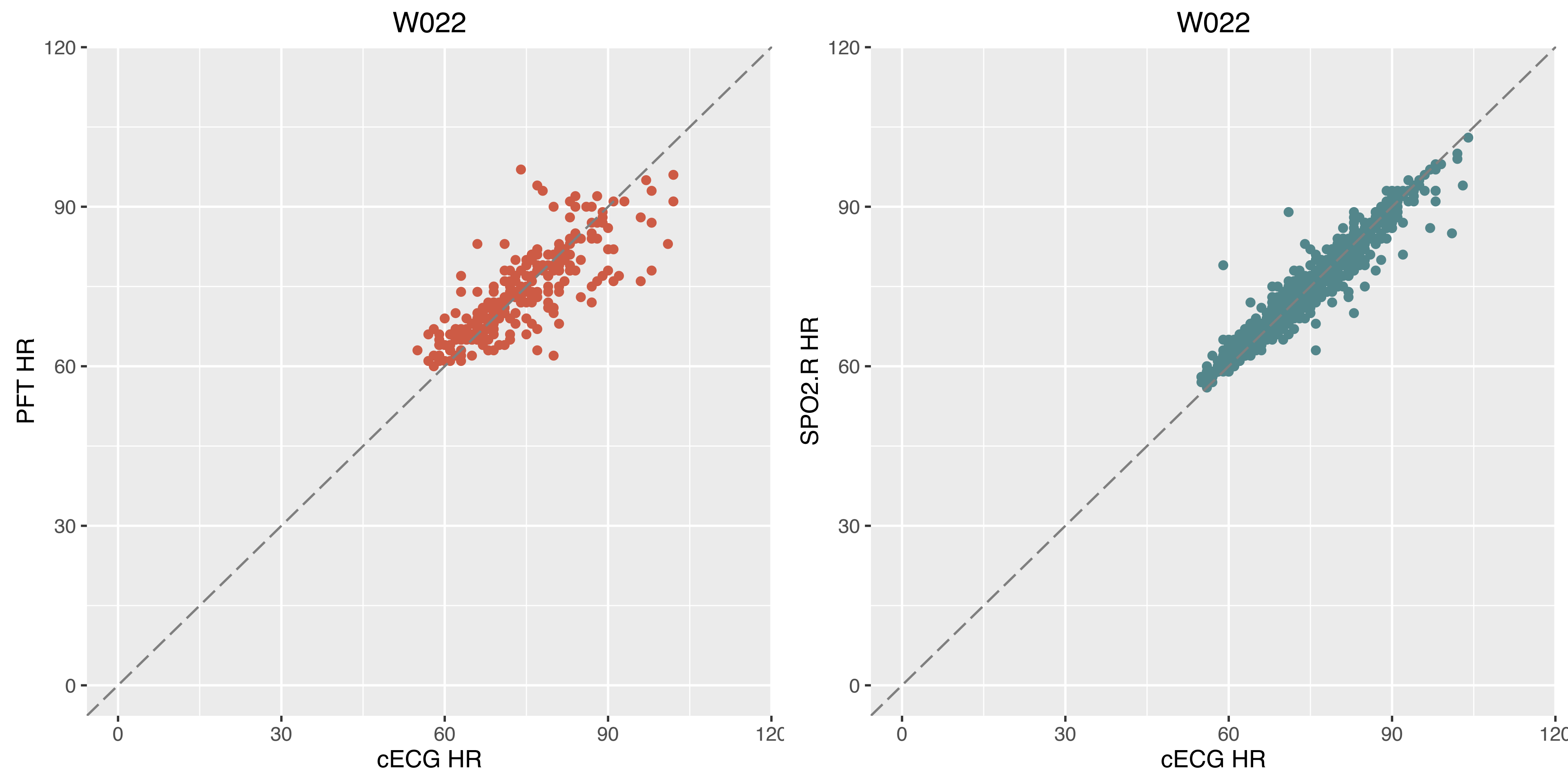

W022
PFT HR
cECG HR
0
30
60
90
120
W022
SPO2.R HR
cECG HR

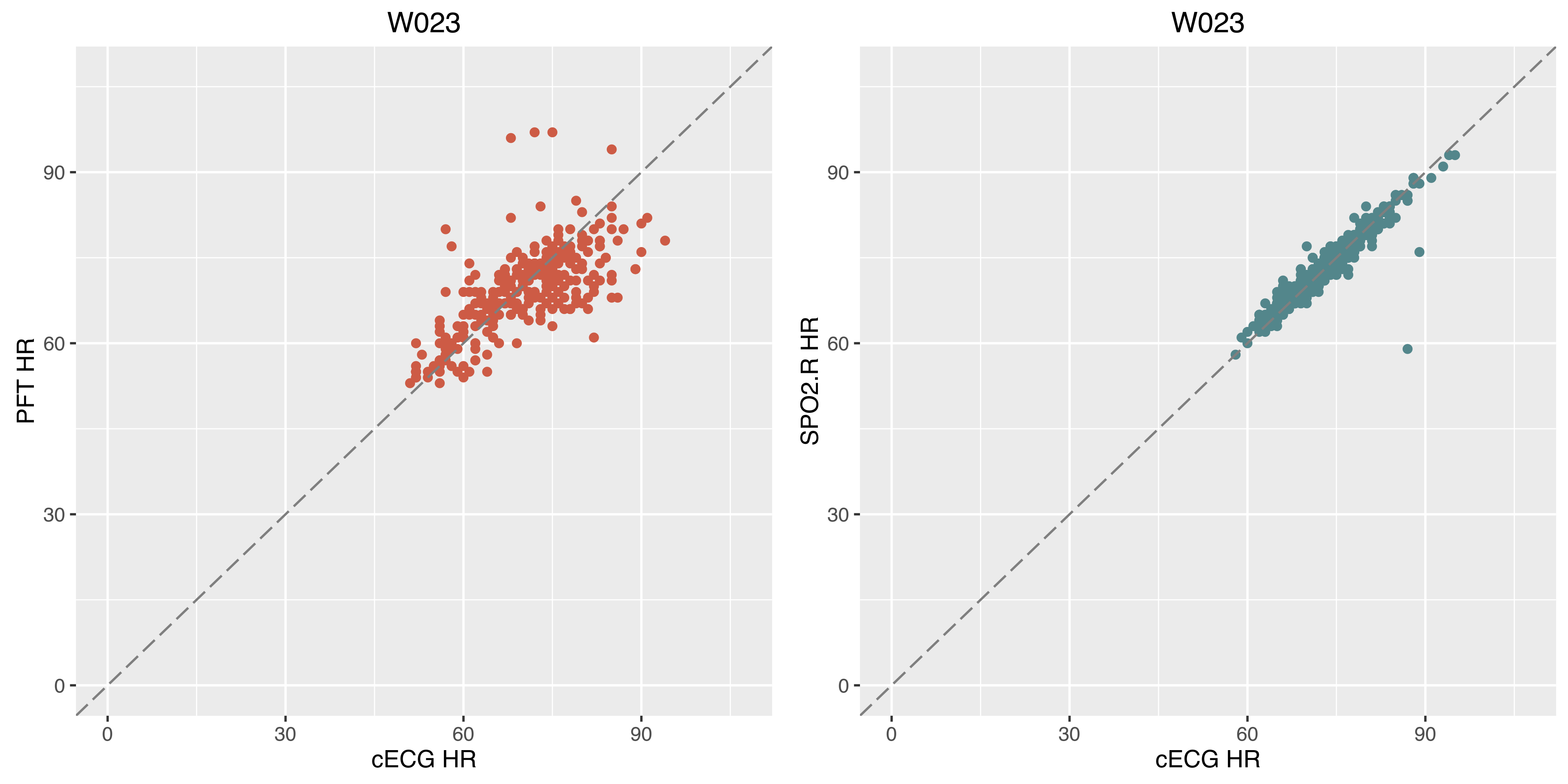

W023
PFT HR
cECG HR
0
30
60
90
W023
SPO2.R HR
cECG HR

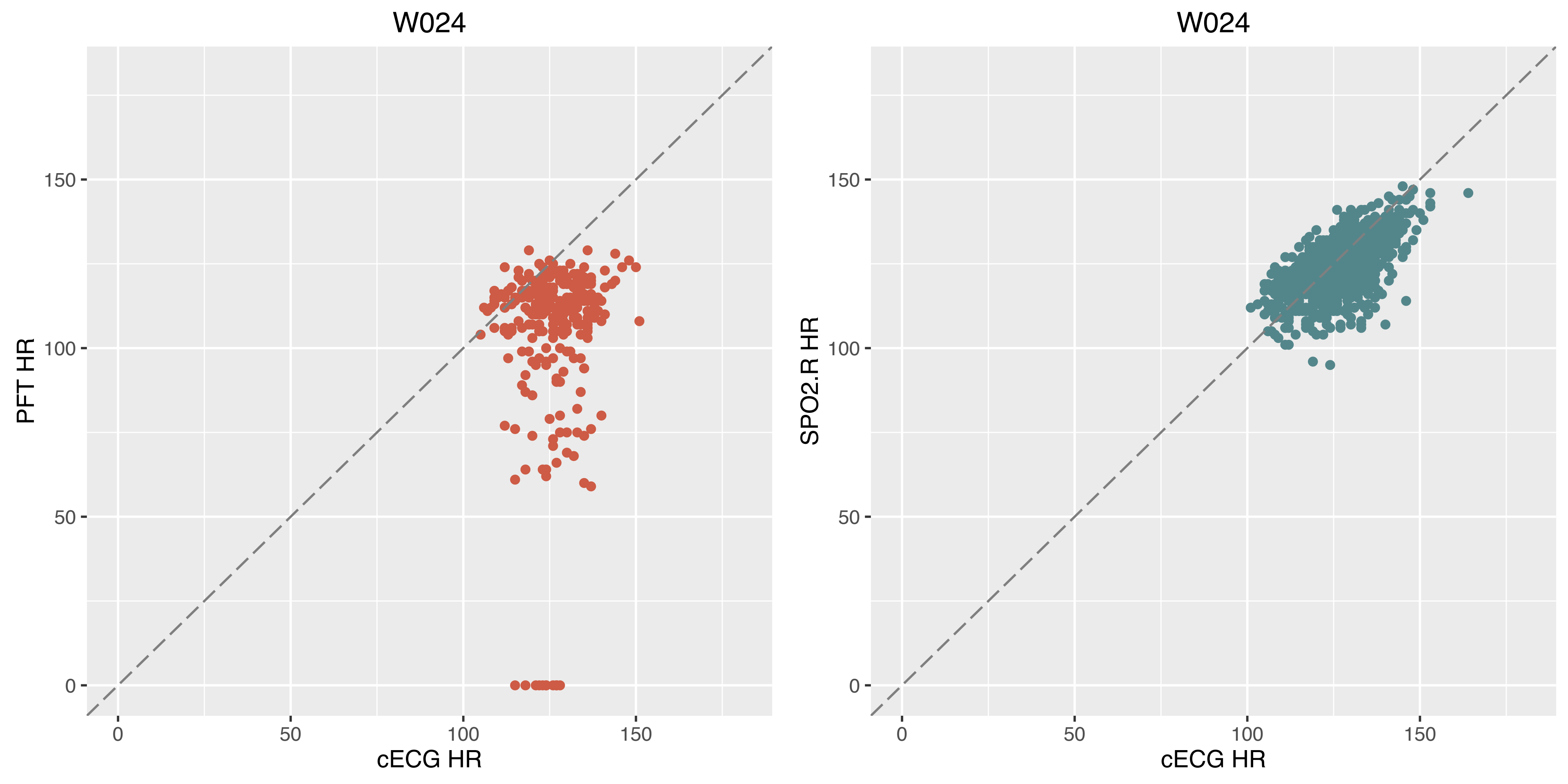
W024
PFT HR
cECG HR
150
100
50
0
W024
SPO2.R HR
cECG HR

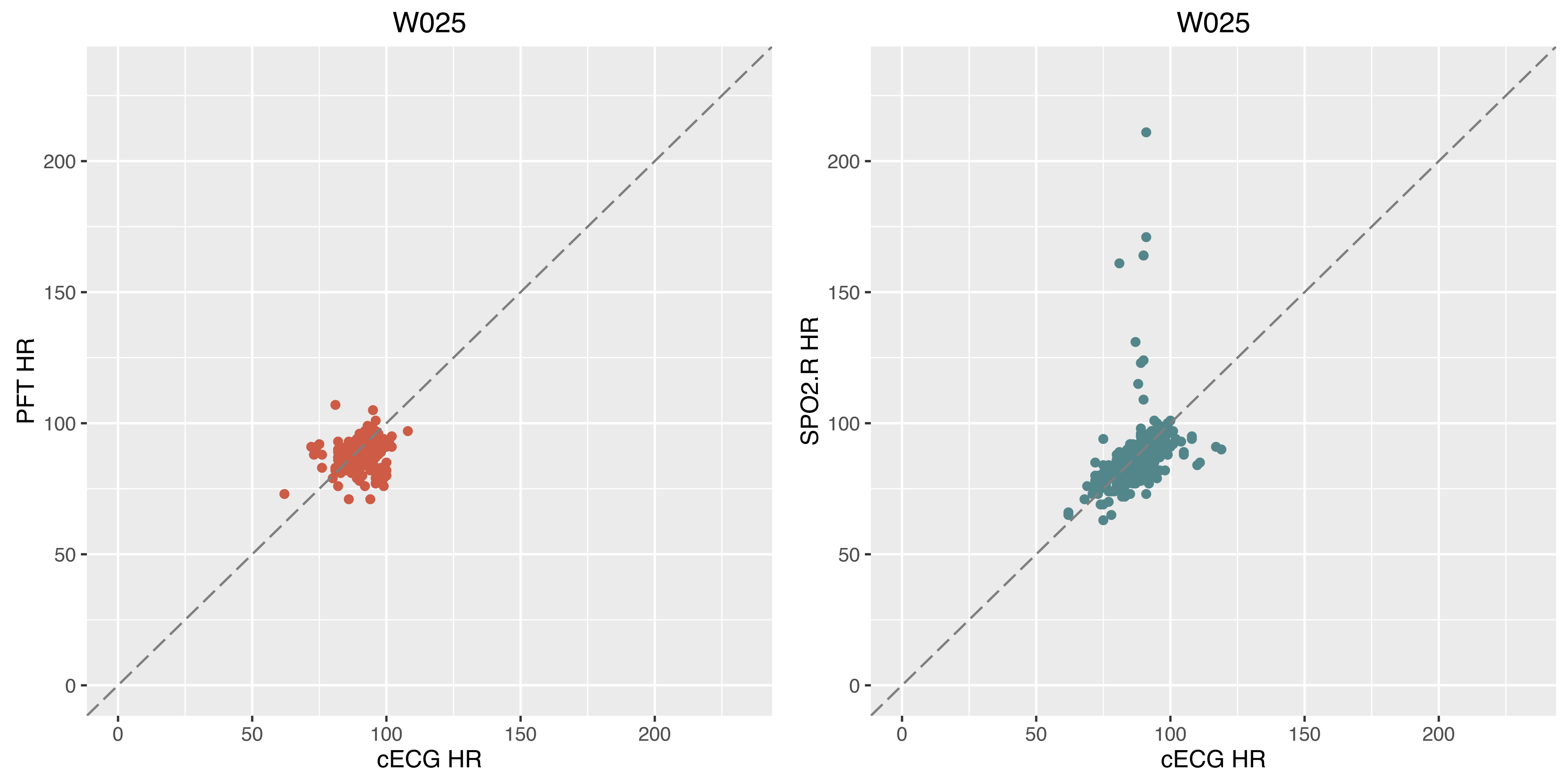
W025
PFT HR
cECG HR
W025
SPO2.R HR
cECG HR
0
50
100
150
200

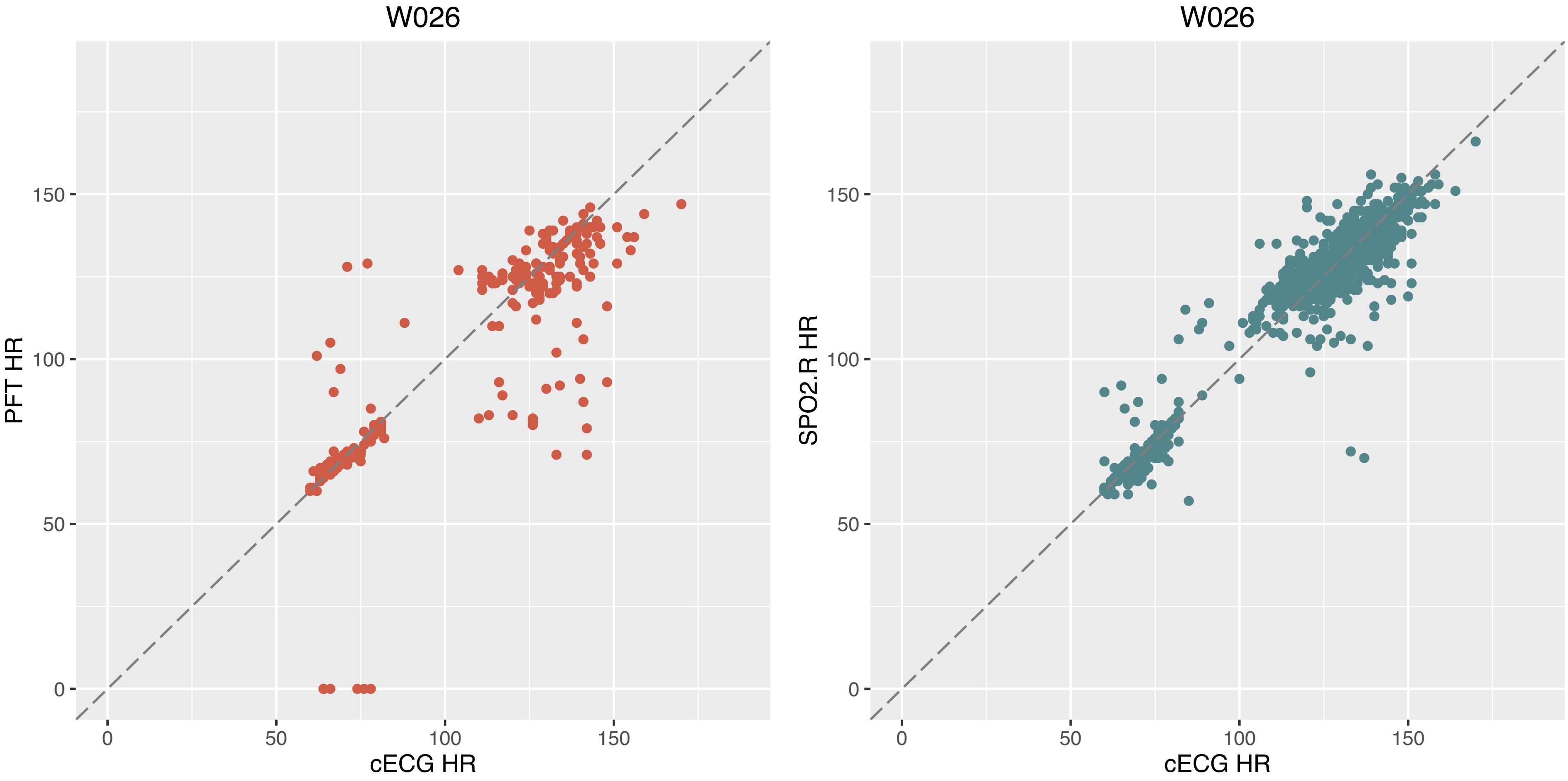
W026
PFT HR
cECG HR
150
100
50
0
0
50
100
150
W026
SPO2.R HR
cECG HR
150
100
50
0
0
50
100
150

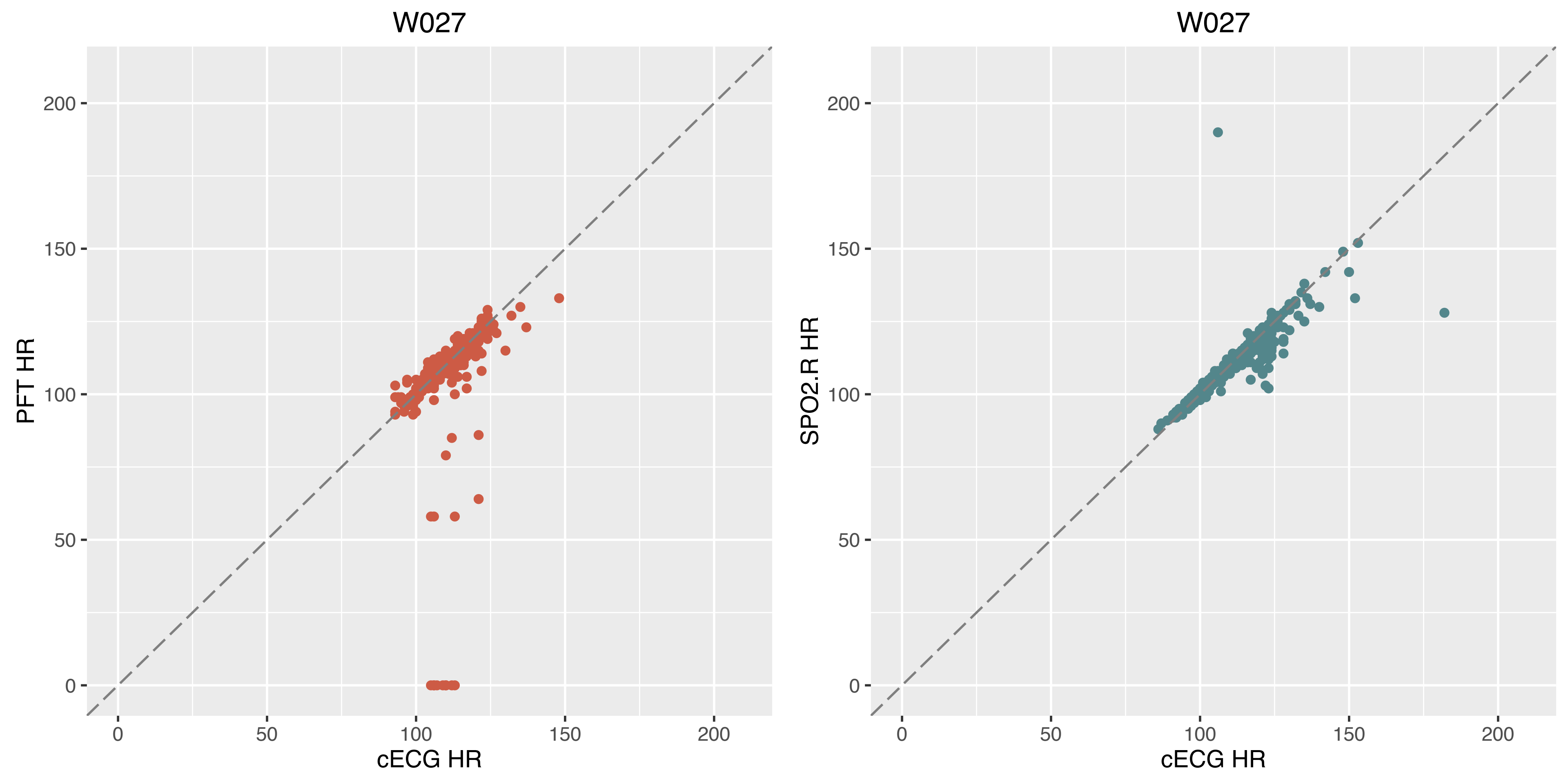
W027
PFT HR
cECG HR
W027
SPO2.R HR
cECG HR
0
50
100
150
200

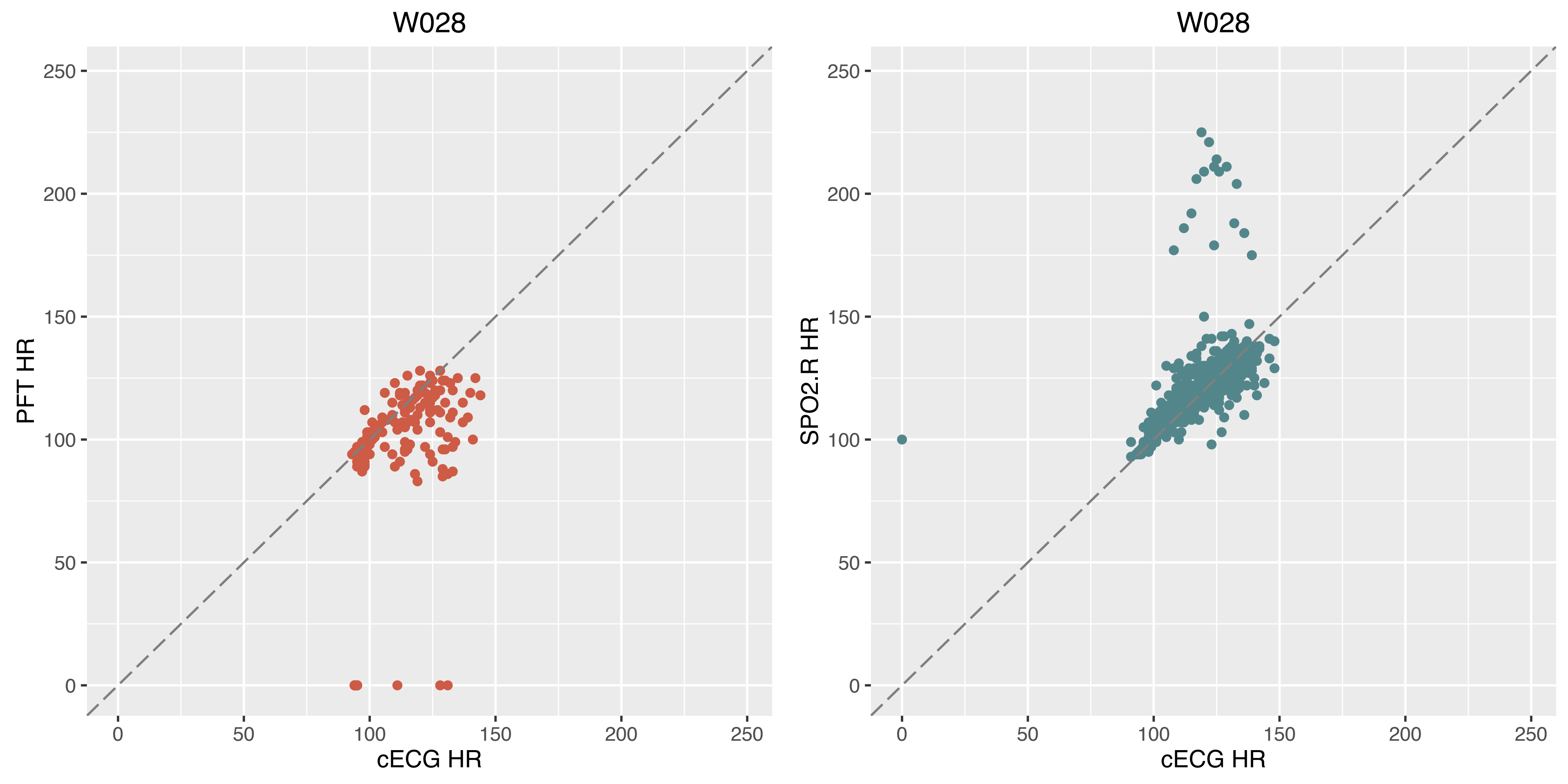
W028
PFT HR
cECG HR
W028
SPO2.R HR
cECG HR
0
50
100
150
200
250

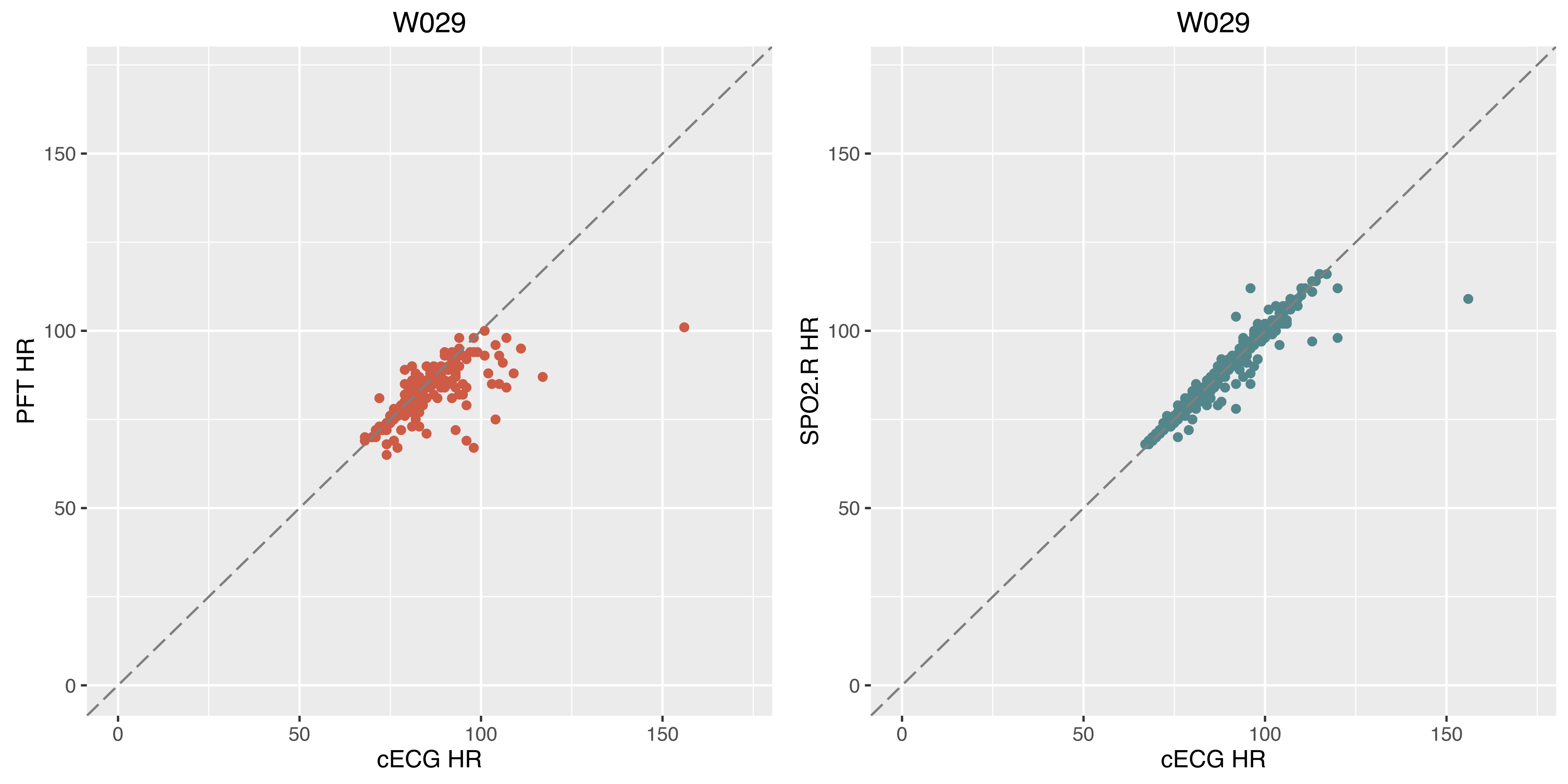
W029
PFT HR
cECG HR
150
100
50
0
0
50
100
150
W029
SPO2.R HR
cECG HR
150
100
50
0
0
50
100
150

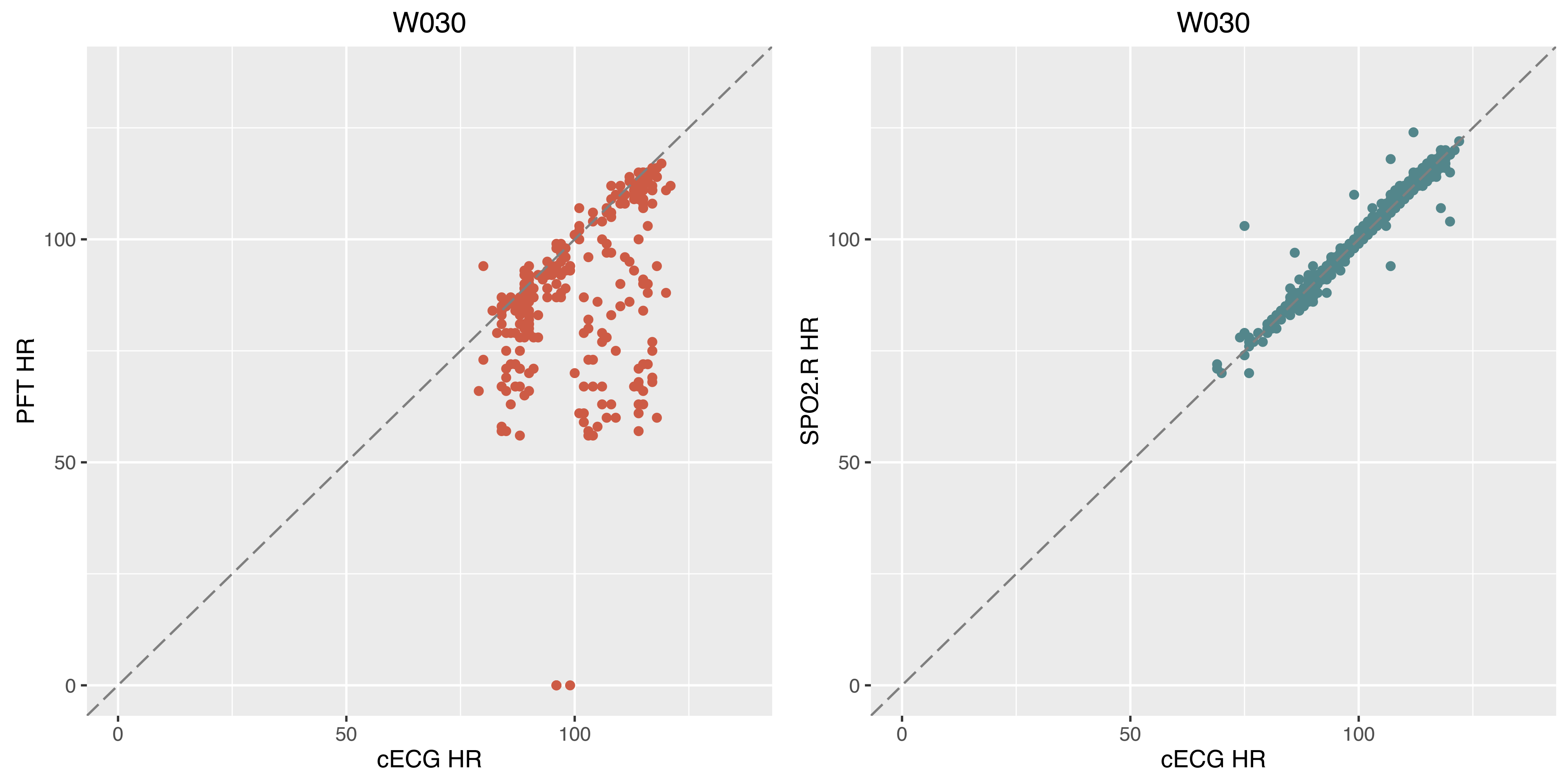
W030
PFT HR
cECG HR
0
50
100
W030
SPO2.R HR
cECG HR
0
50
100

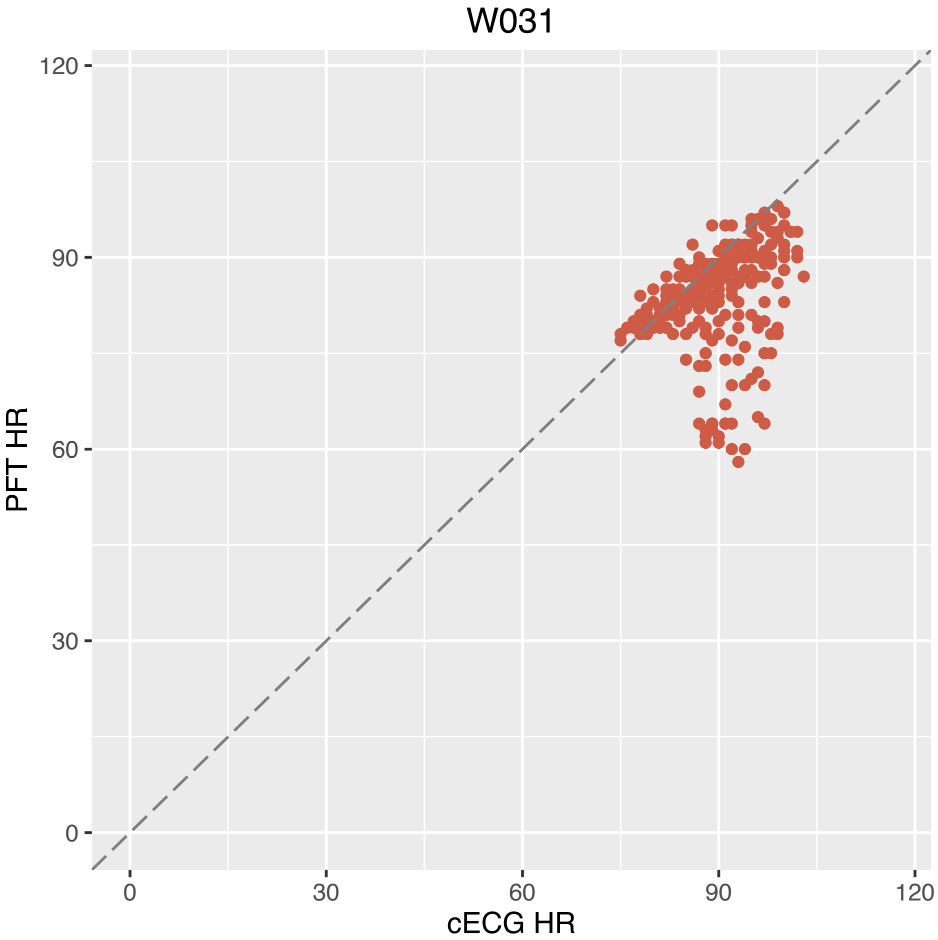
W031
PFT HR
cECG HR
0
30
60
90
120
0
30
60
90
120
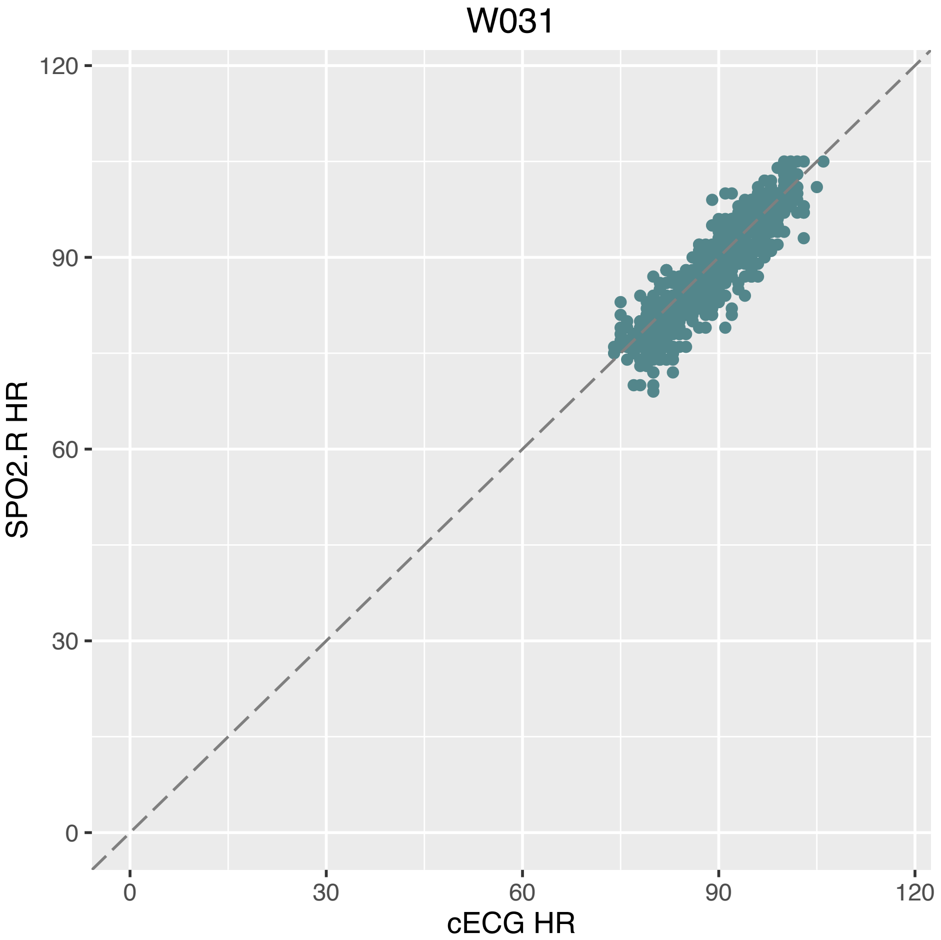
W031
SPO2.R HR
cECG HR
0
30
60
90
120
0
30
60
90
120

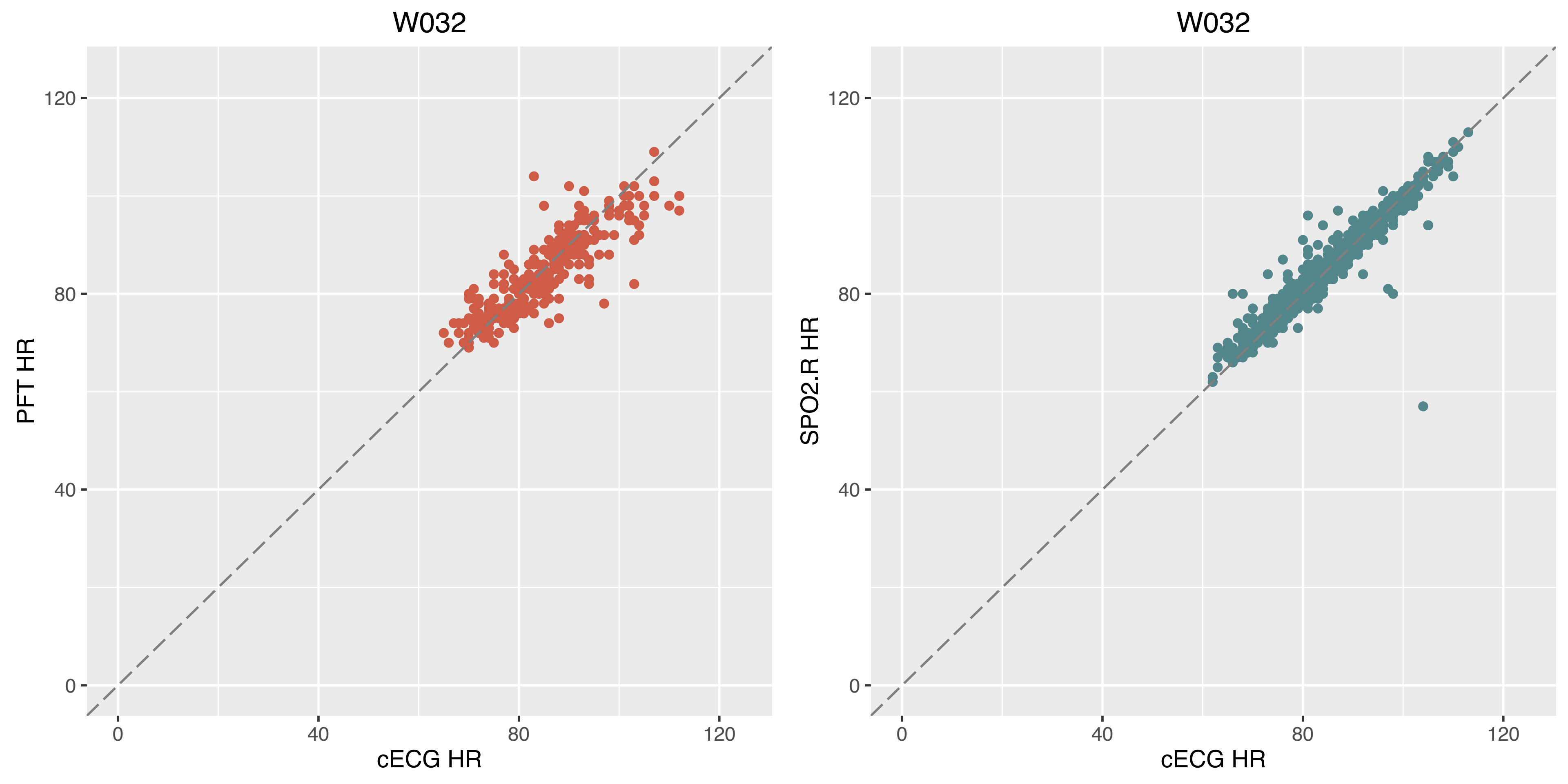
W032
PFT HR
cECG HR
0
40
80
120
W032
SPO2.R HR
cECG HR
0
40
80
120

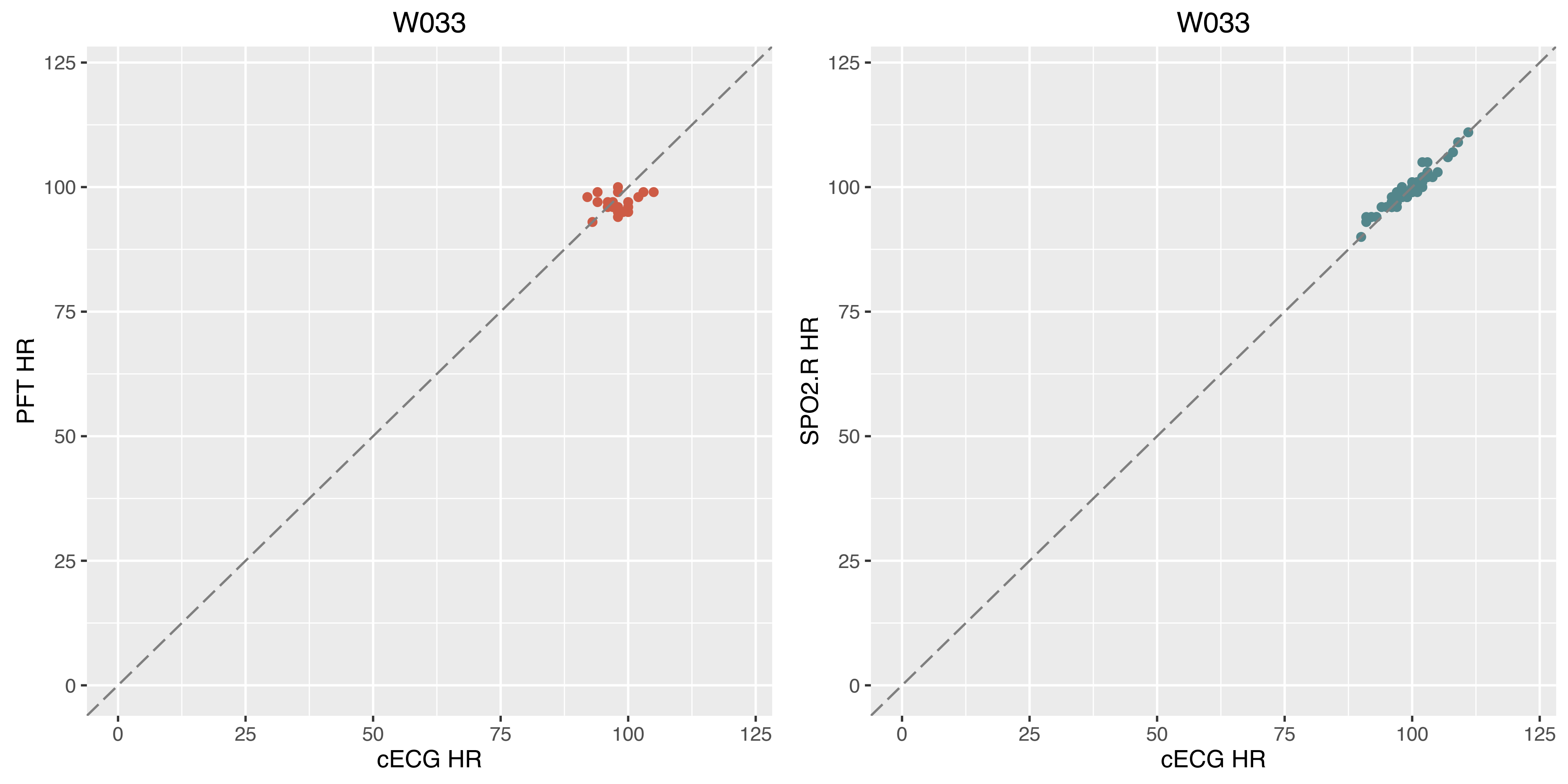
W033
PFT HR
cECG HR
W033
SPO2.R HR
cECG HR
0
25
50
75
100
125

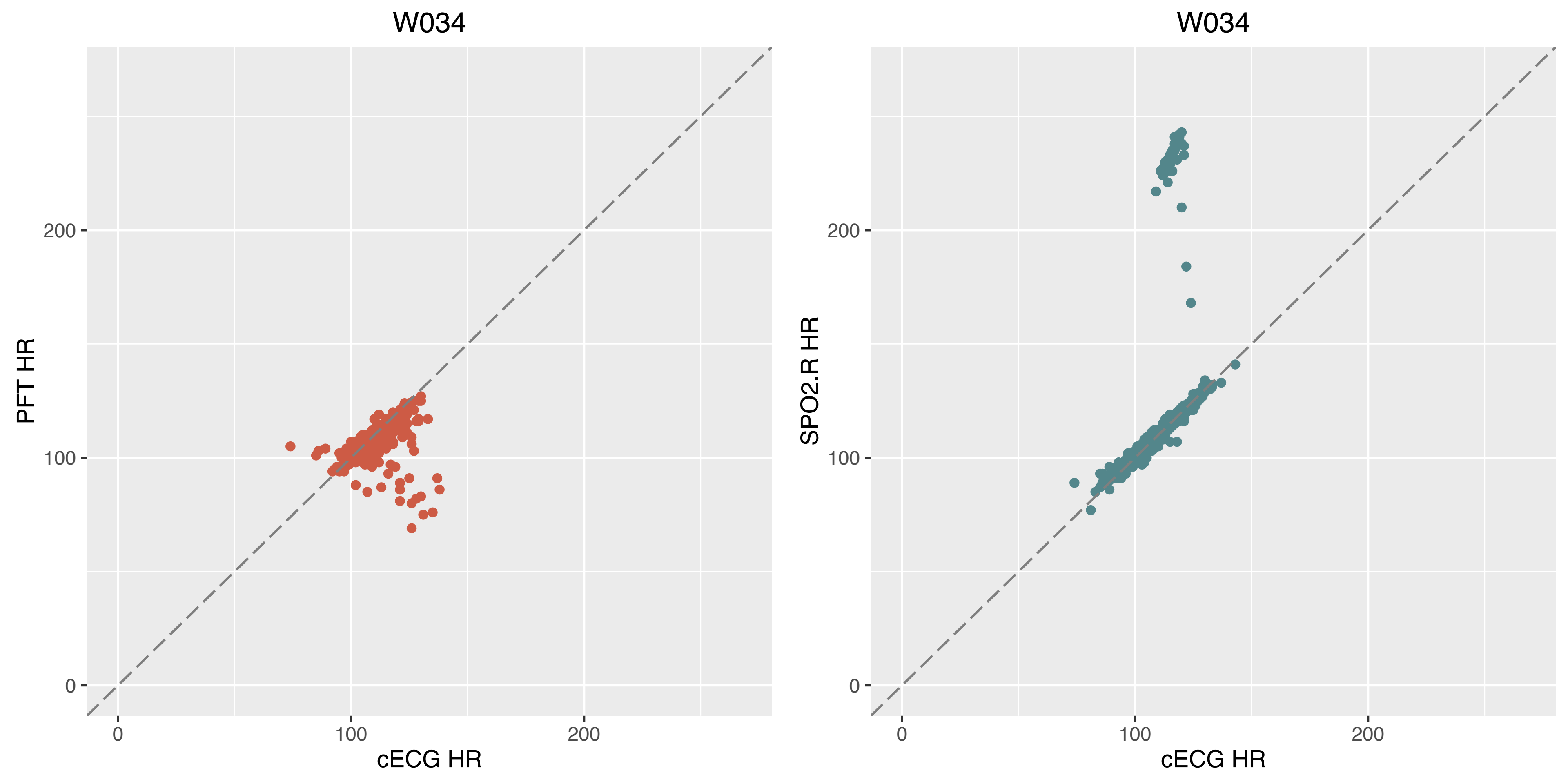

W034
PFT HR
200
100
0
0
100
200
cECG HR
W034
SPO2.R HR
200
100
0
0
100
200
cECG HR

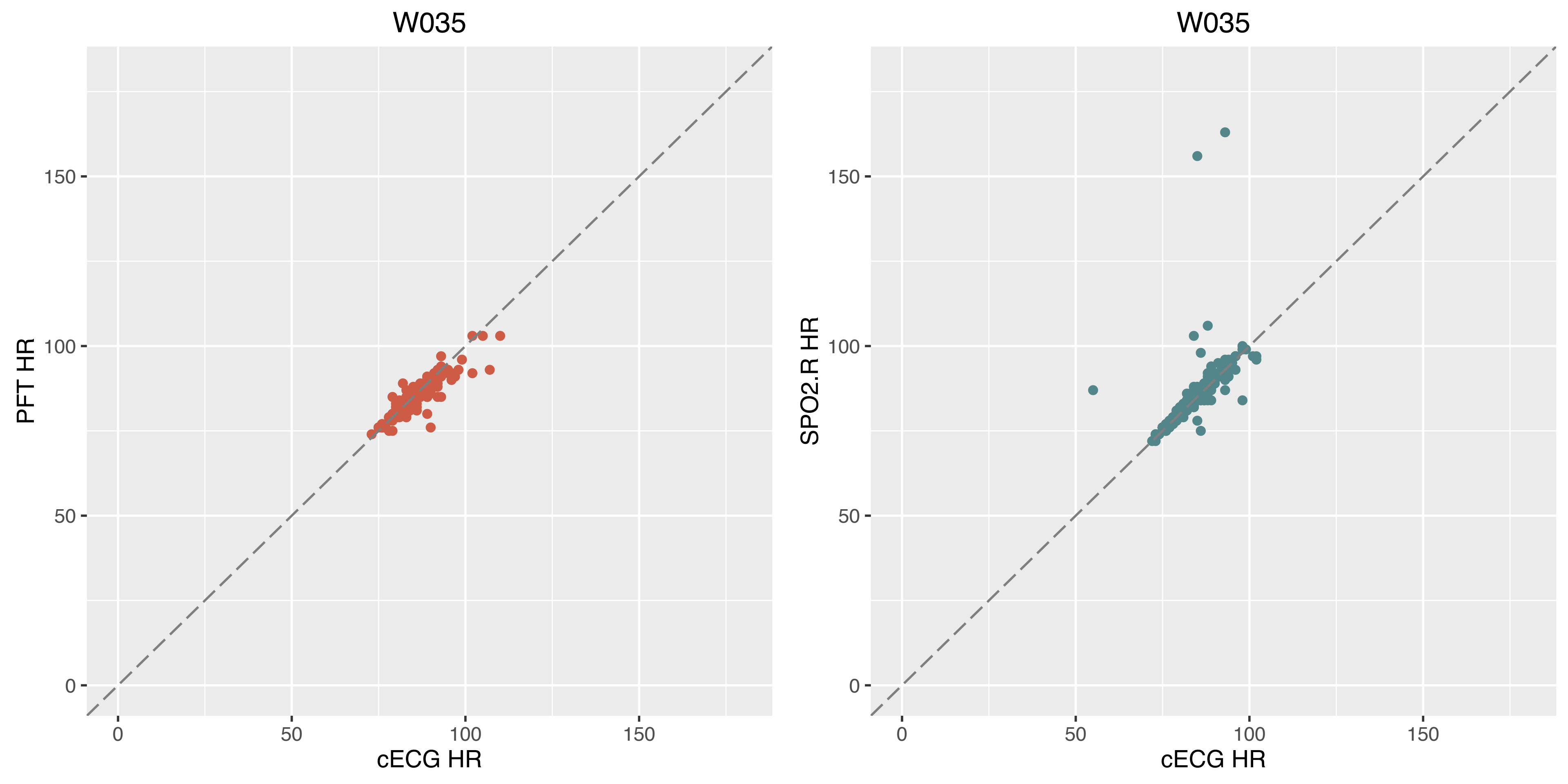
W035
PFT HR
cECG HR
0
50
100
150
W035
SPO2.R HR
cECG HR
0
50
100
150

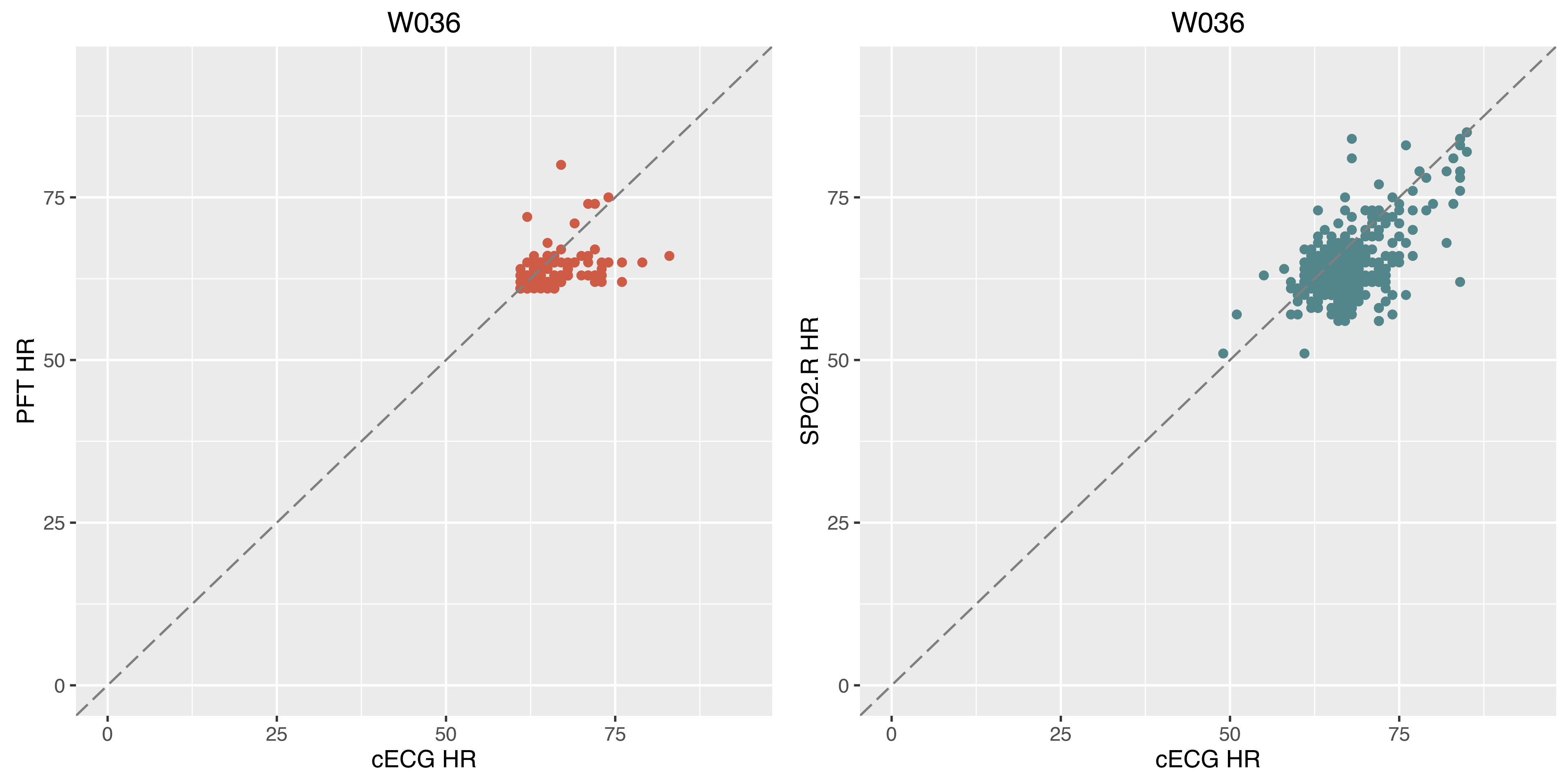
W036
PFT HR
cECG HR
0
25
50
75
W036
SPO2.R HR
cECG HR
0
25
50
75

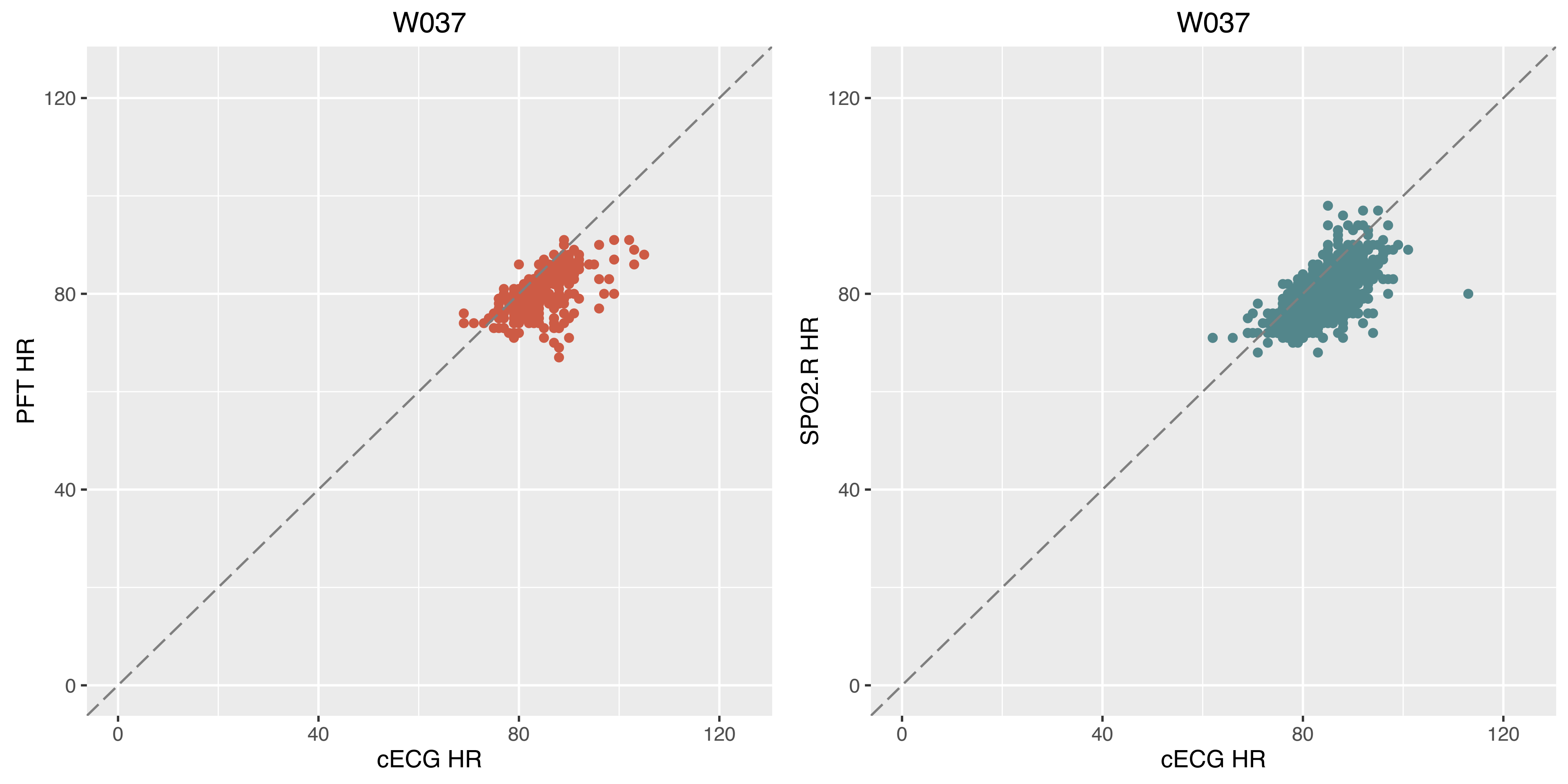
W037
W037
PFT HR
SPO2.R HR
cECG HR
cECG HR
0
40
80
120

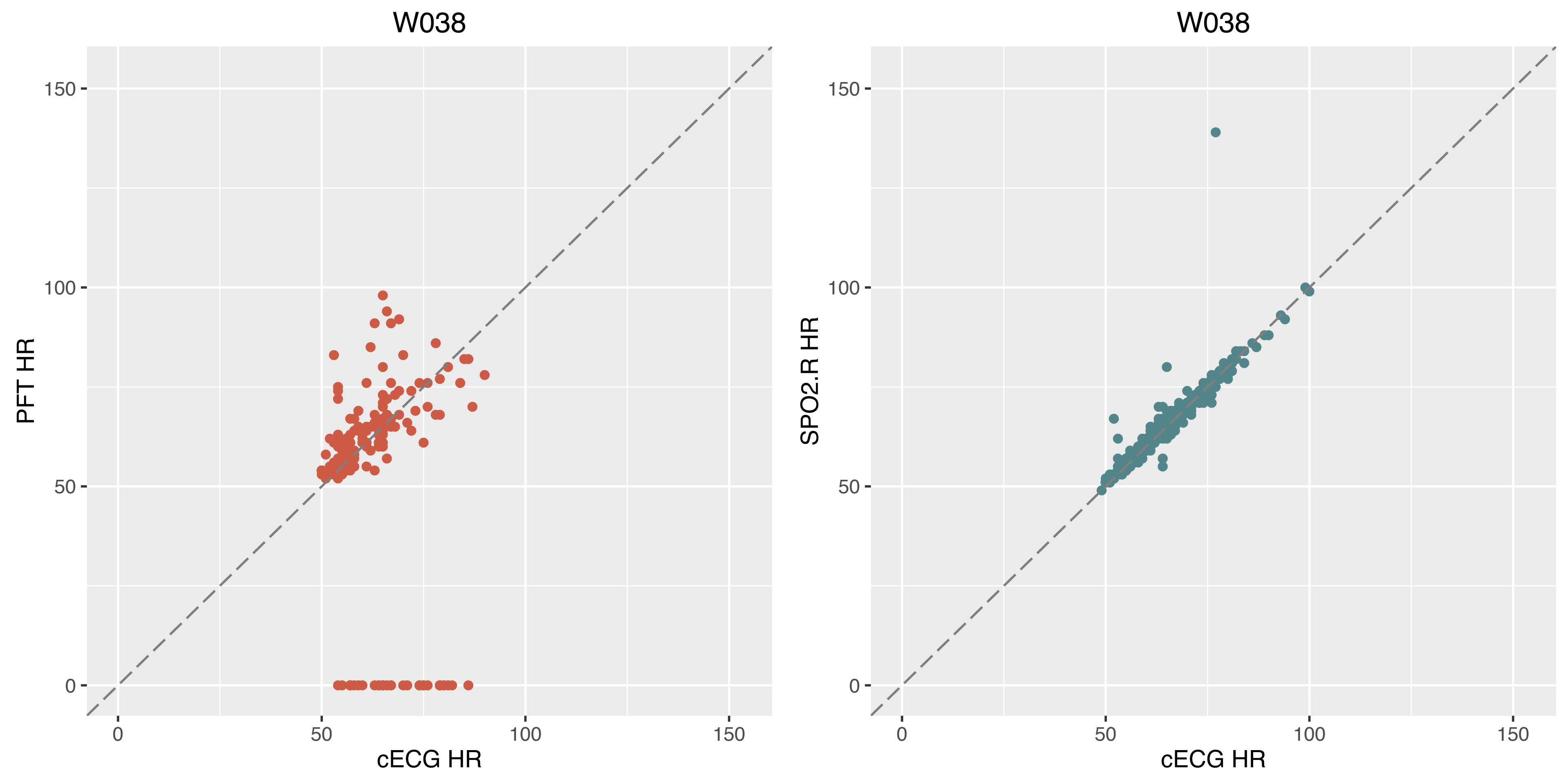
W038
PFT HR
cECG HR
W038
SPO2.R HR
cECG HR
0
50
100
150

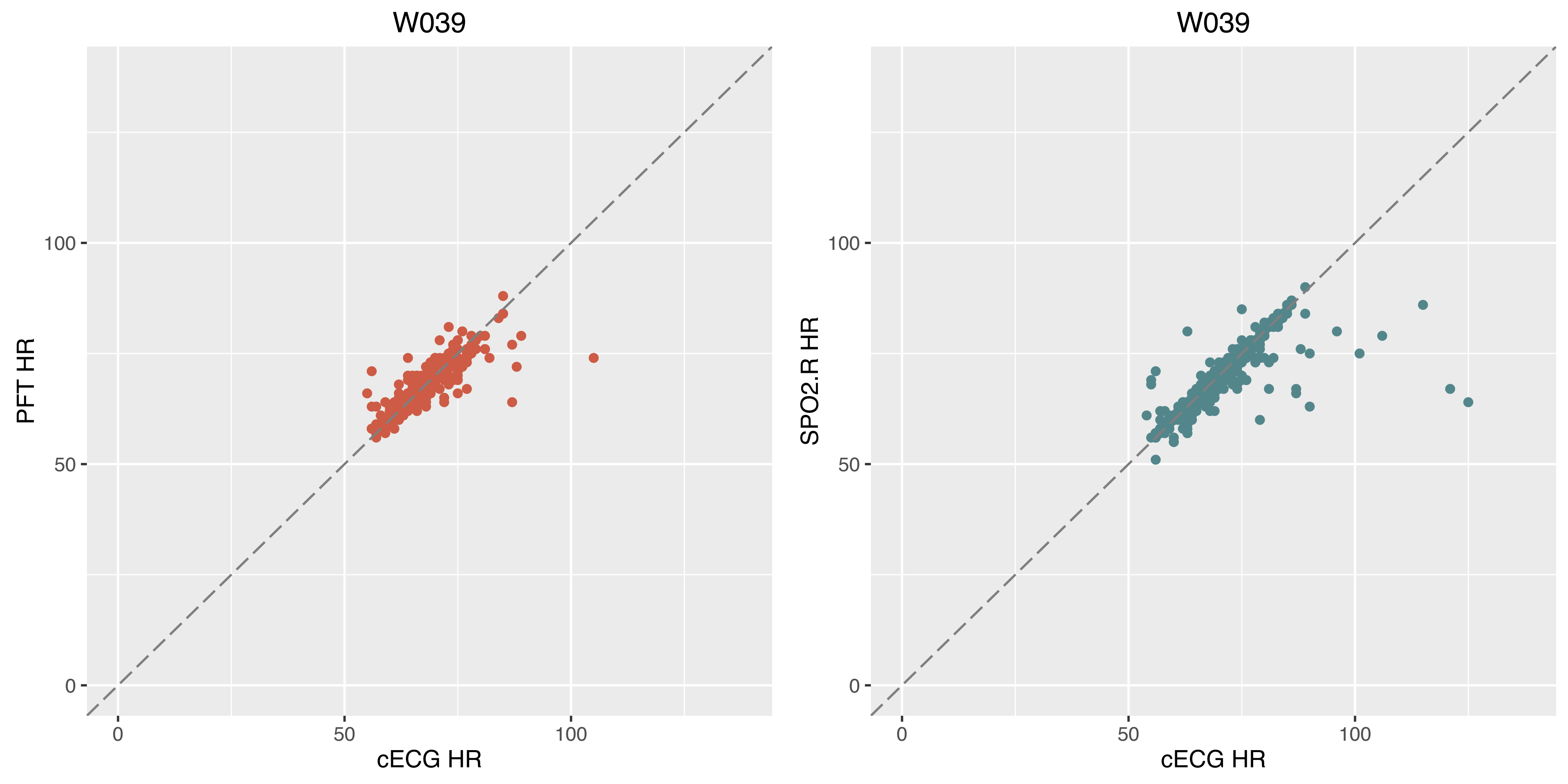
W039
W039
PFT HR
SPO2.R HR
cECG HR
cECG HR
0
50
100

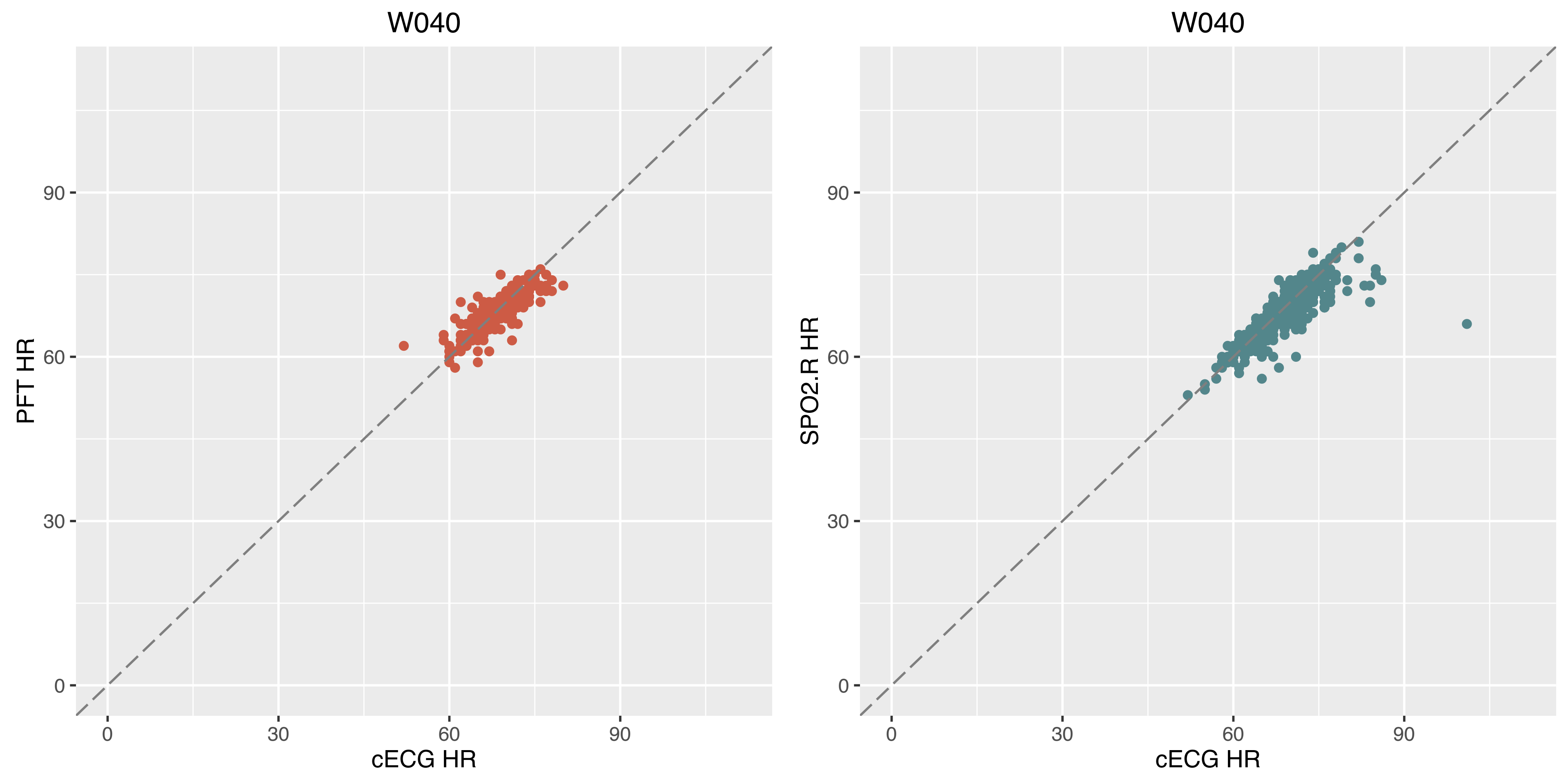

W040
PFT HR
cECG HR
0
30
60
90
W040
SPO2.R HR
cECG HR

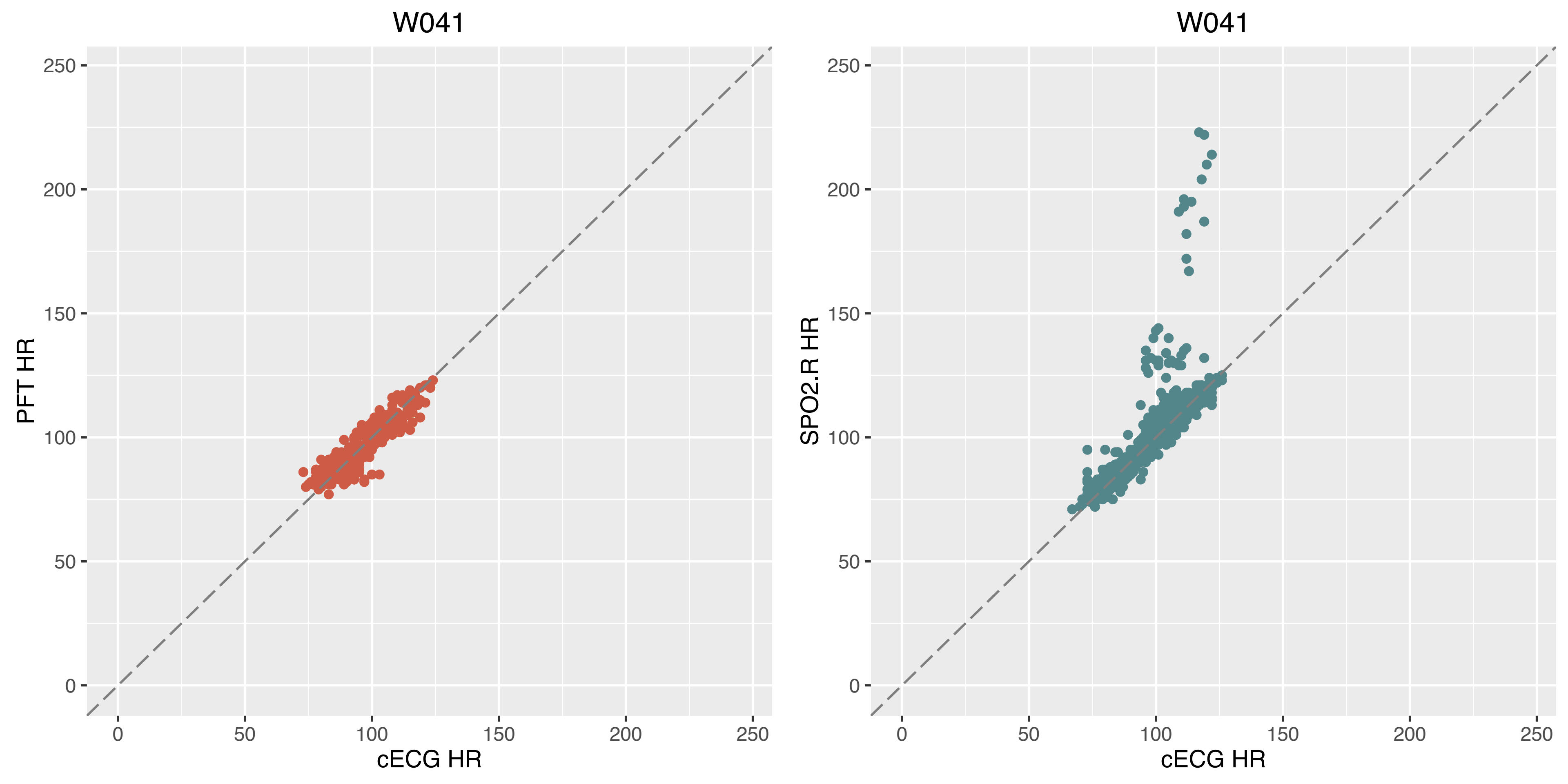

W041
PFT HR
cECG HR
W041
SPO2.R HR
cECG HR
0
50
100
150
200
250

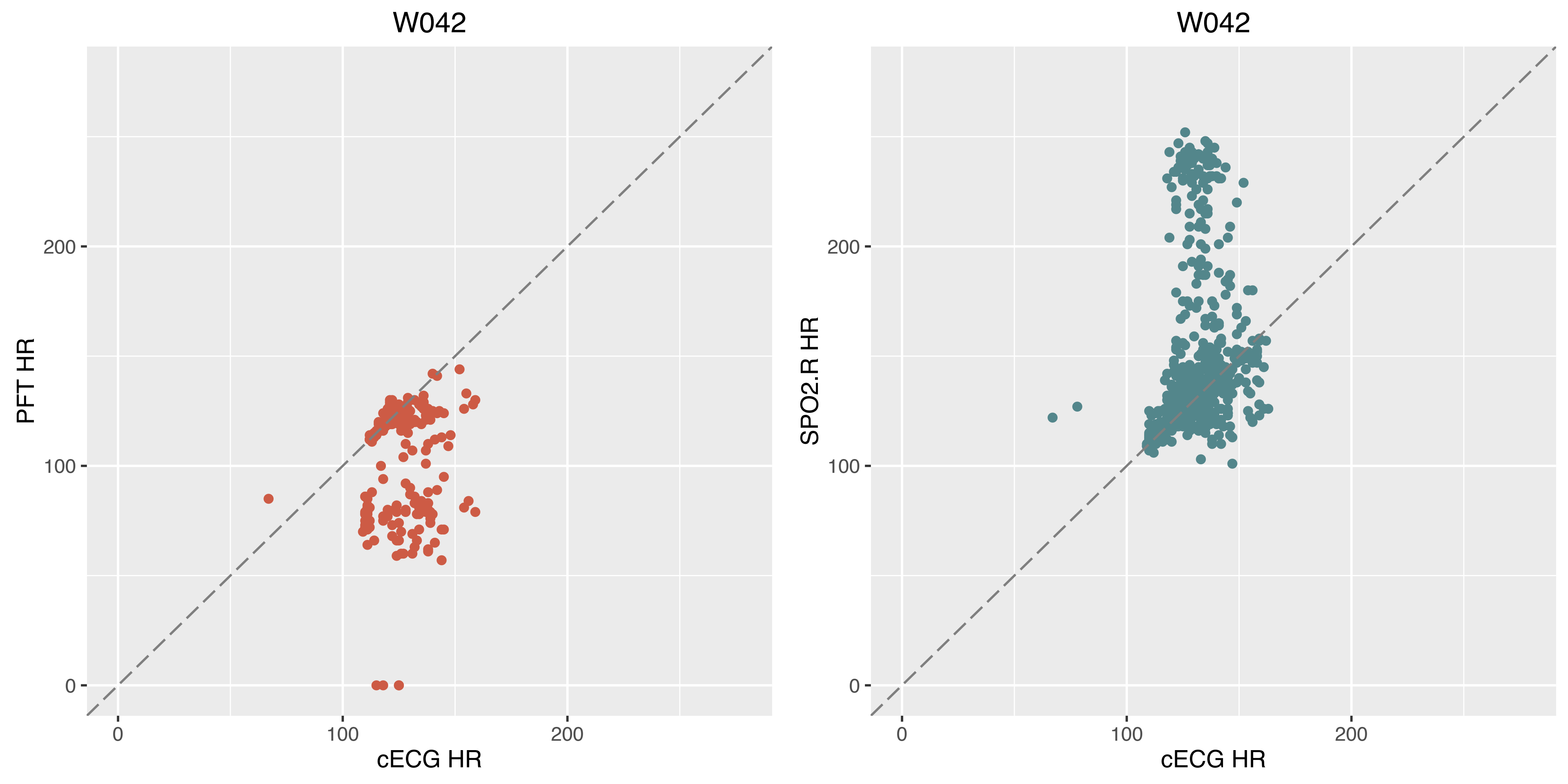

W042
PFT HR
cECG HR
0
100
200
W042
SPO2.R HR
cECG HR
0
100
200

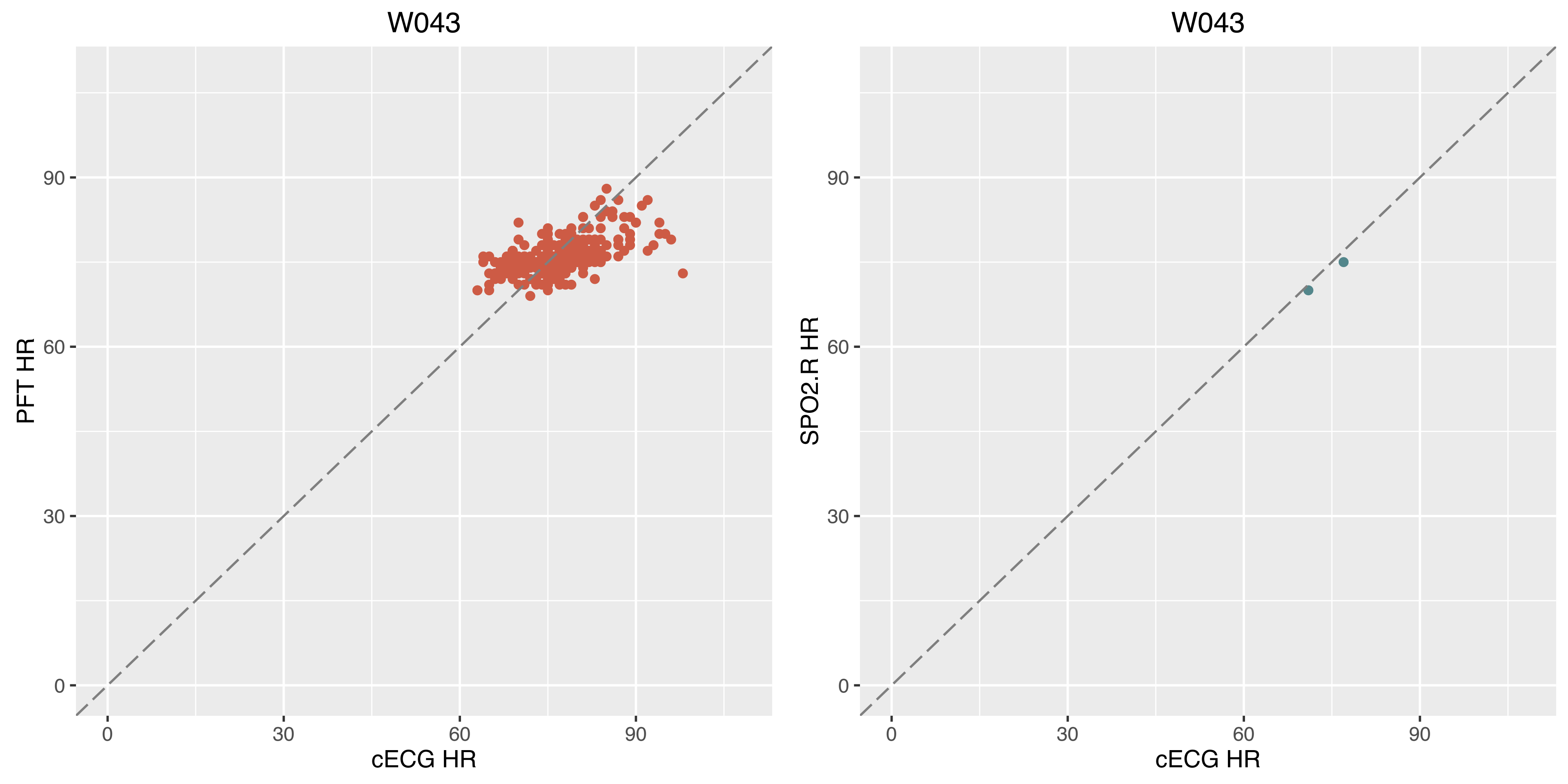
W043
PFT HR
cECG HR
0
30
60
90
W043
SPO2.R HR
cECG HR
0
30
60
90

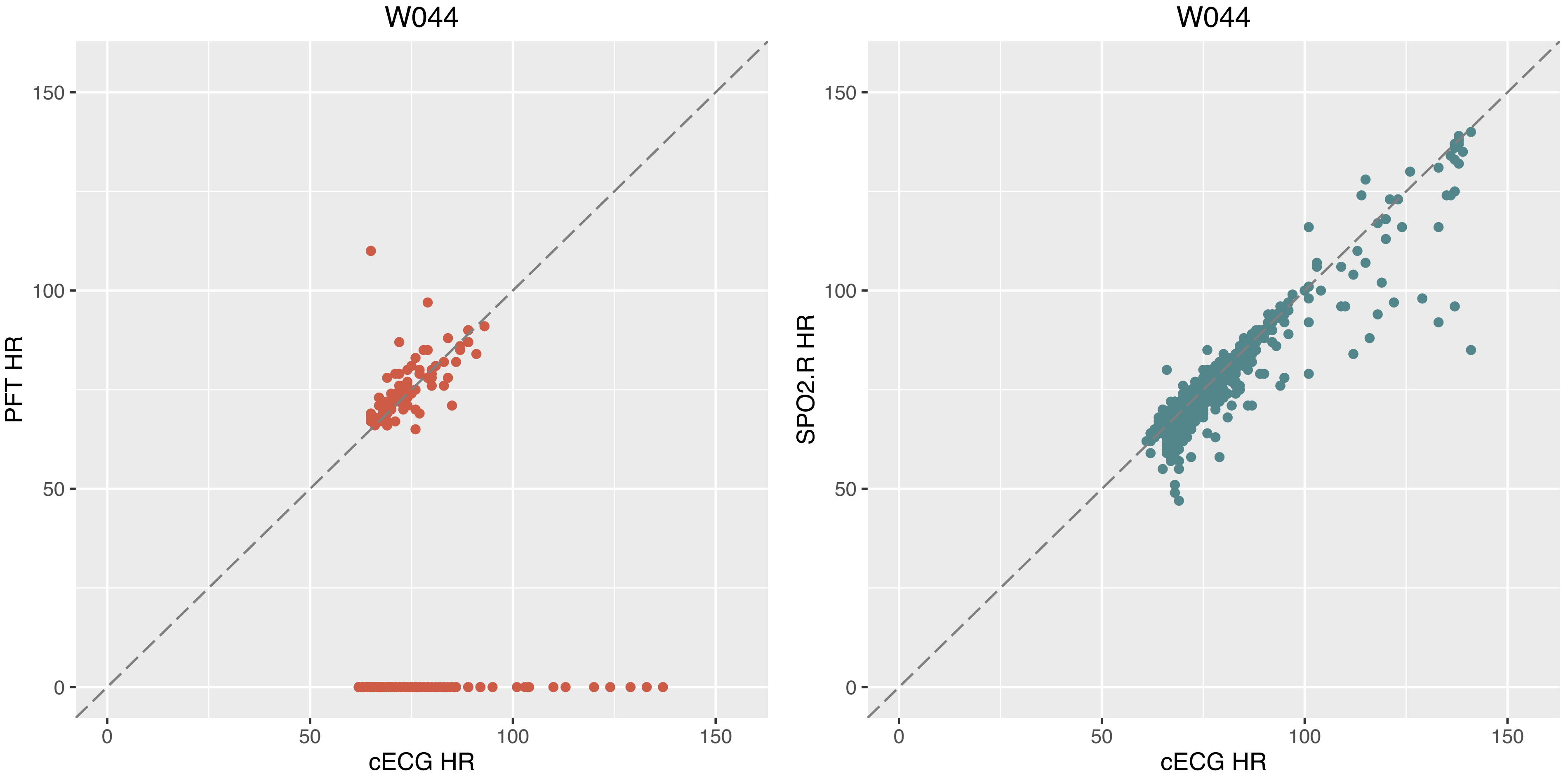
W044
PFT HR
cECG HR
W044
SPO2.R HR
cECG HR
0
50
100
150

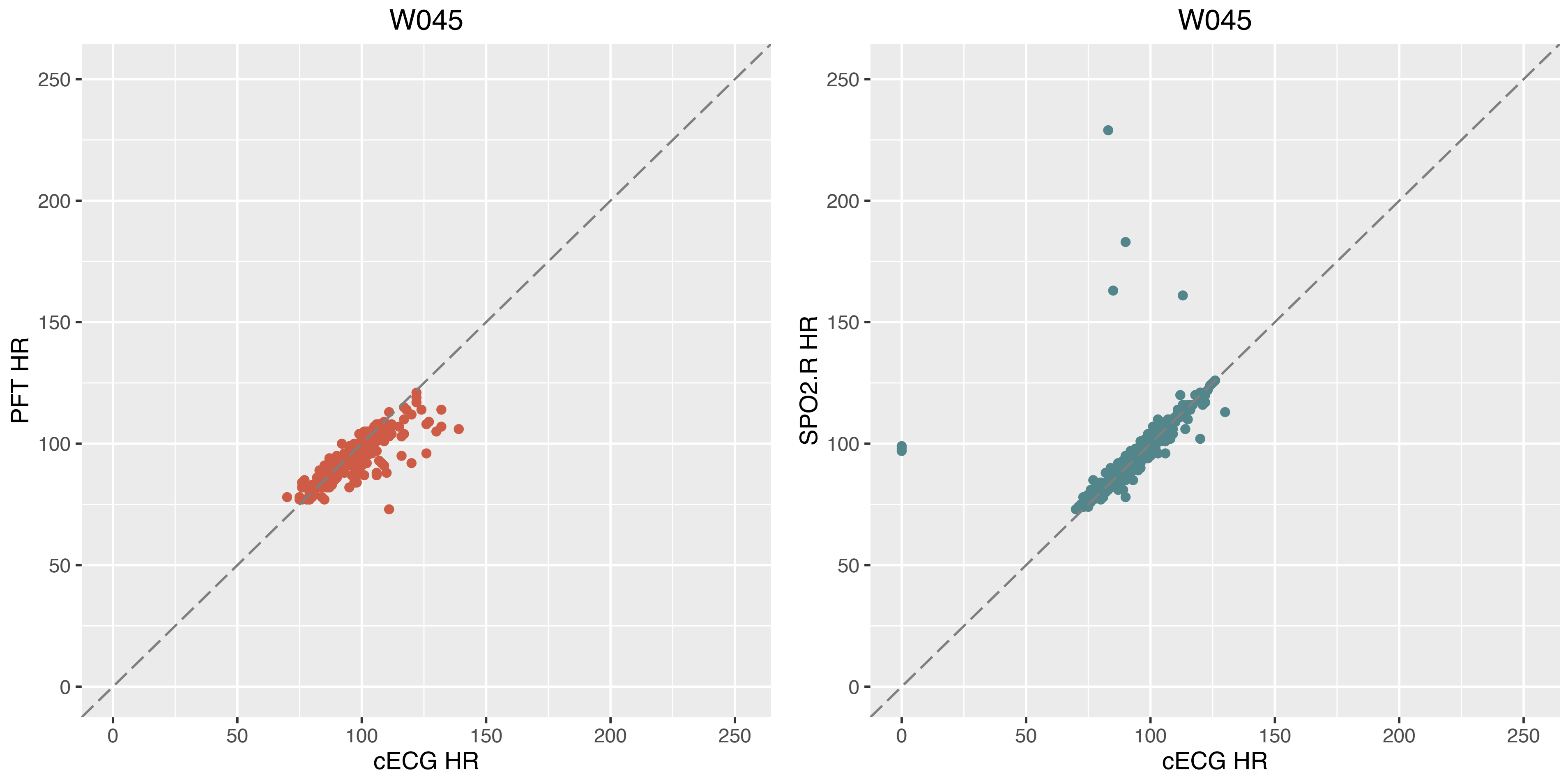
W045
PFT HR
cECG HR
0
50
100
150
200
250
W045
SPO2.R HR
cECG HR
0
50
100
150
200
250

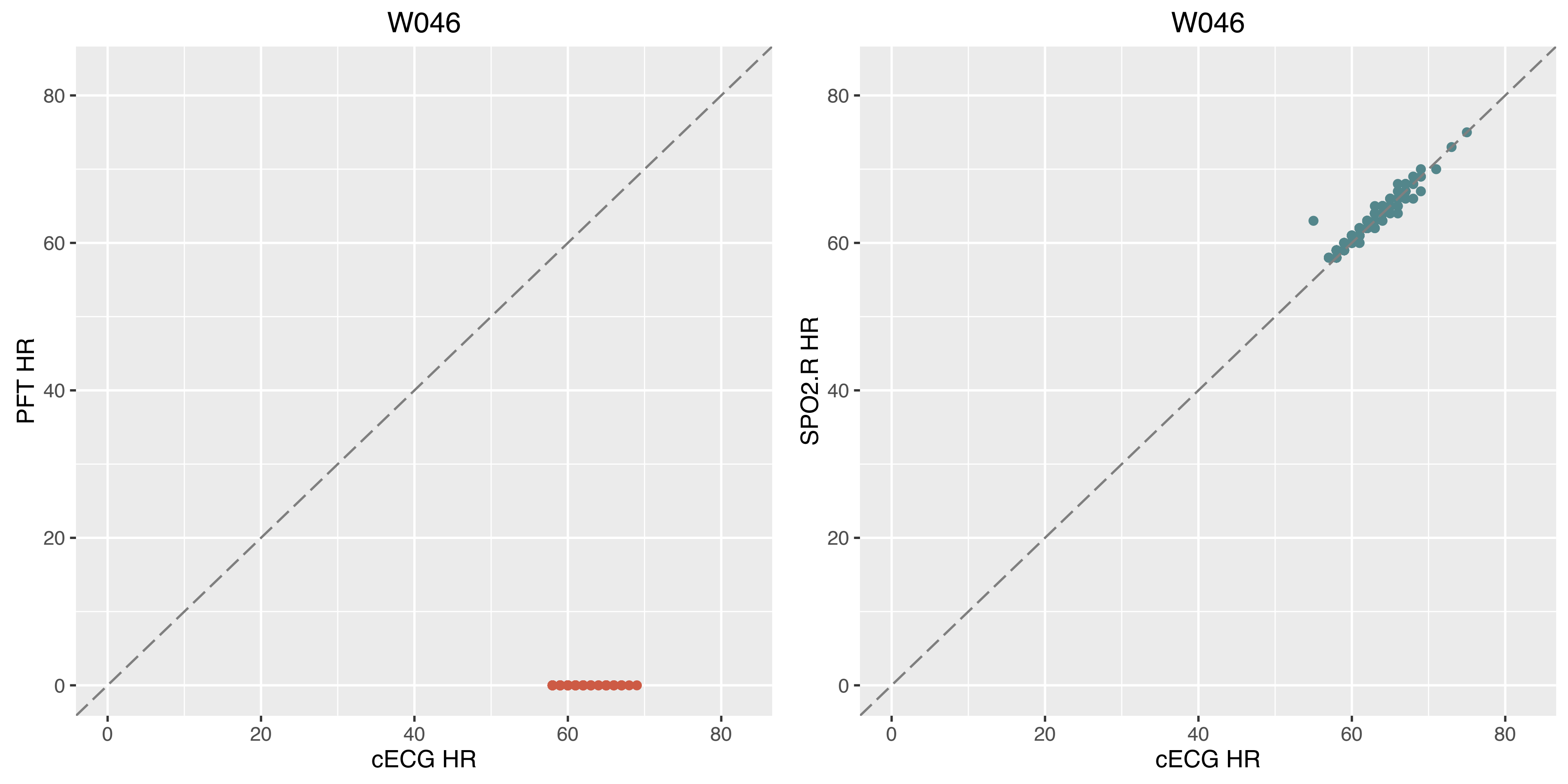
W046
PFT HR
cECG HR
0
20
40
60
80
W046
SPO2.R HR
cECG HR

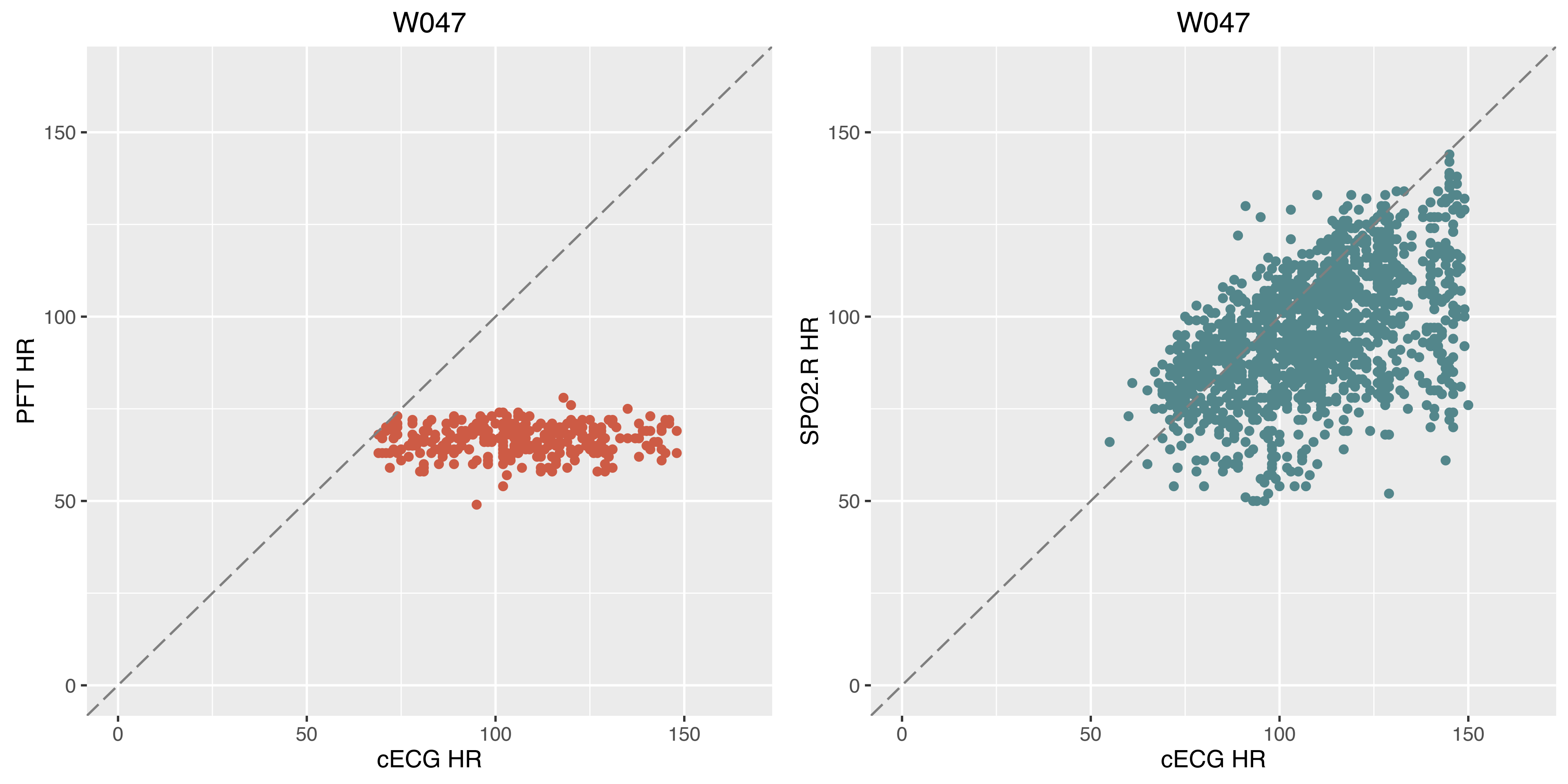

W047
PFT HR
cECG HR
0
50
100
150
W047
SPO2.R HR
cECG HR
0
50
100
150

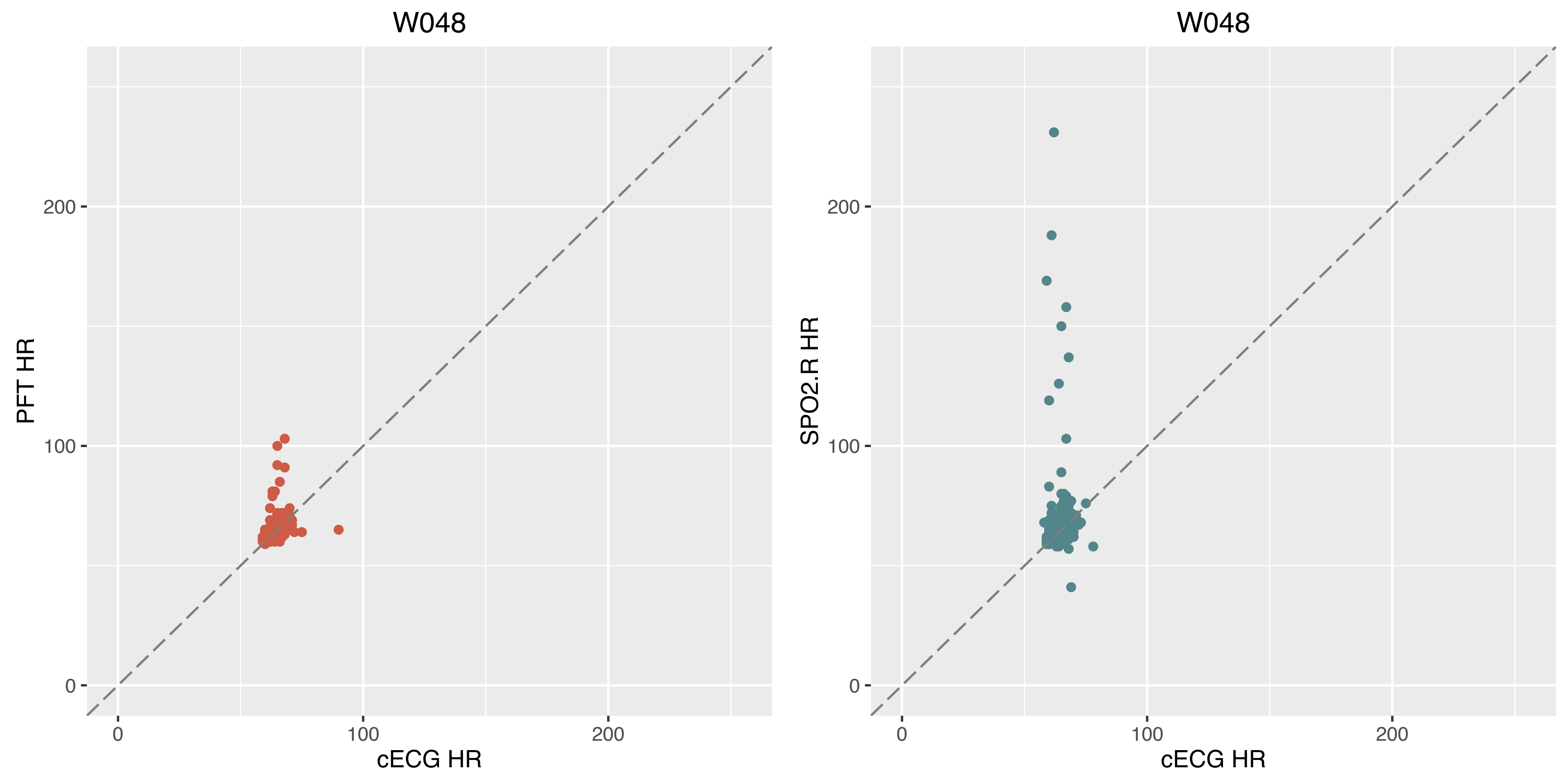

W048
PFT HR
200
100
0
0
100
200
cECG HR
W048
SPO2.R HR
200
100
0
0
100
200
cECG HR

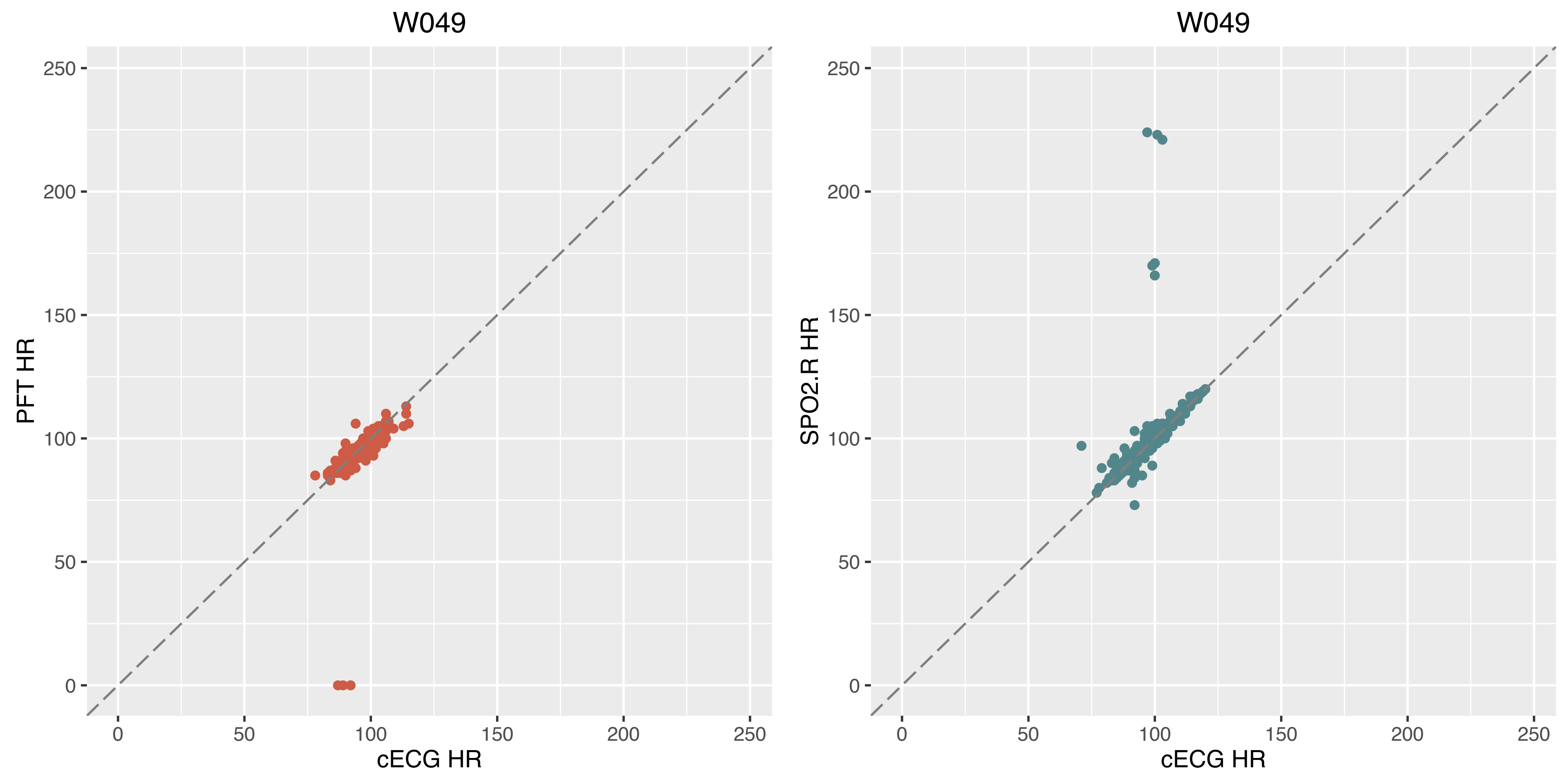

W049
PFT HR
cECG HR
0
50
100
150
200
250
W049
SPO2.R HR
cECG HR

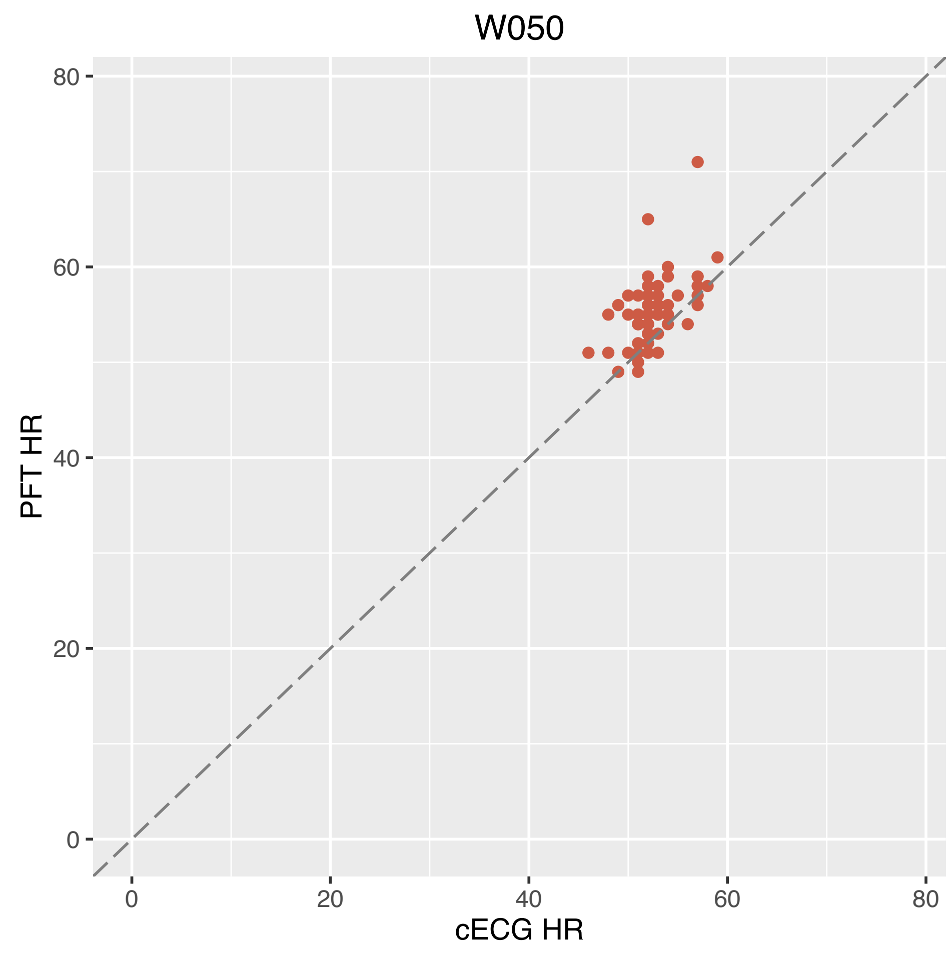
W050
80
60
40
20
0
PFT HR
0
20
40
60
80
cECG HR

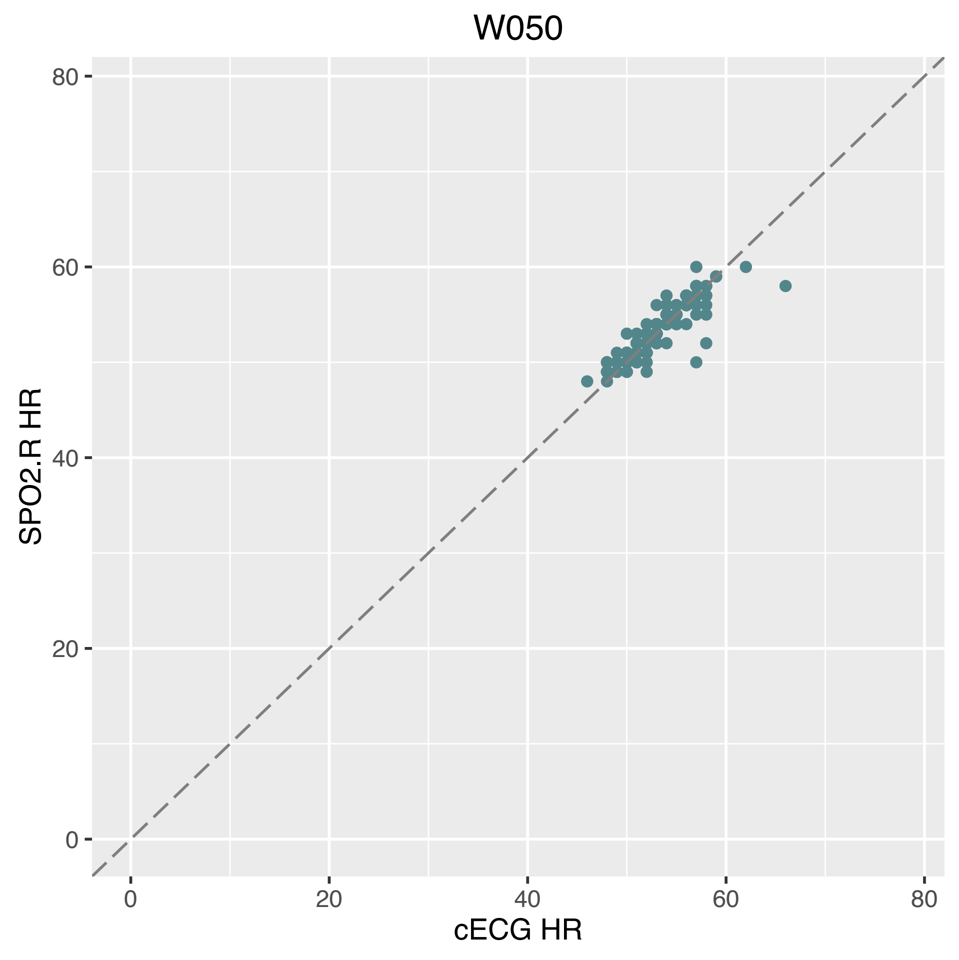
W050
80
60
40
20
0
SPO2.R HR
0
20
40
60
80
cECG HR

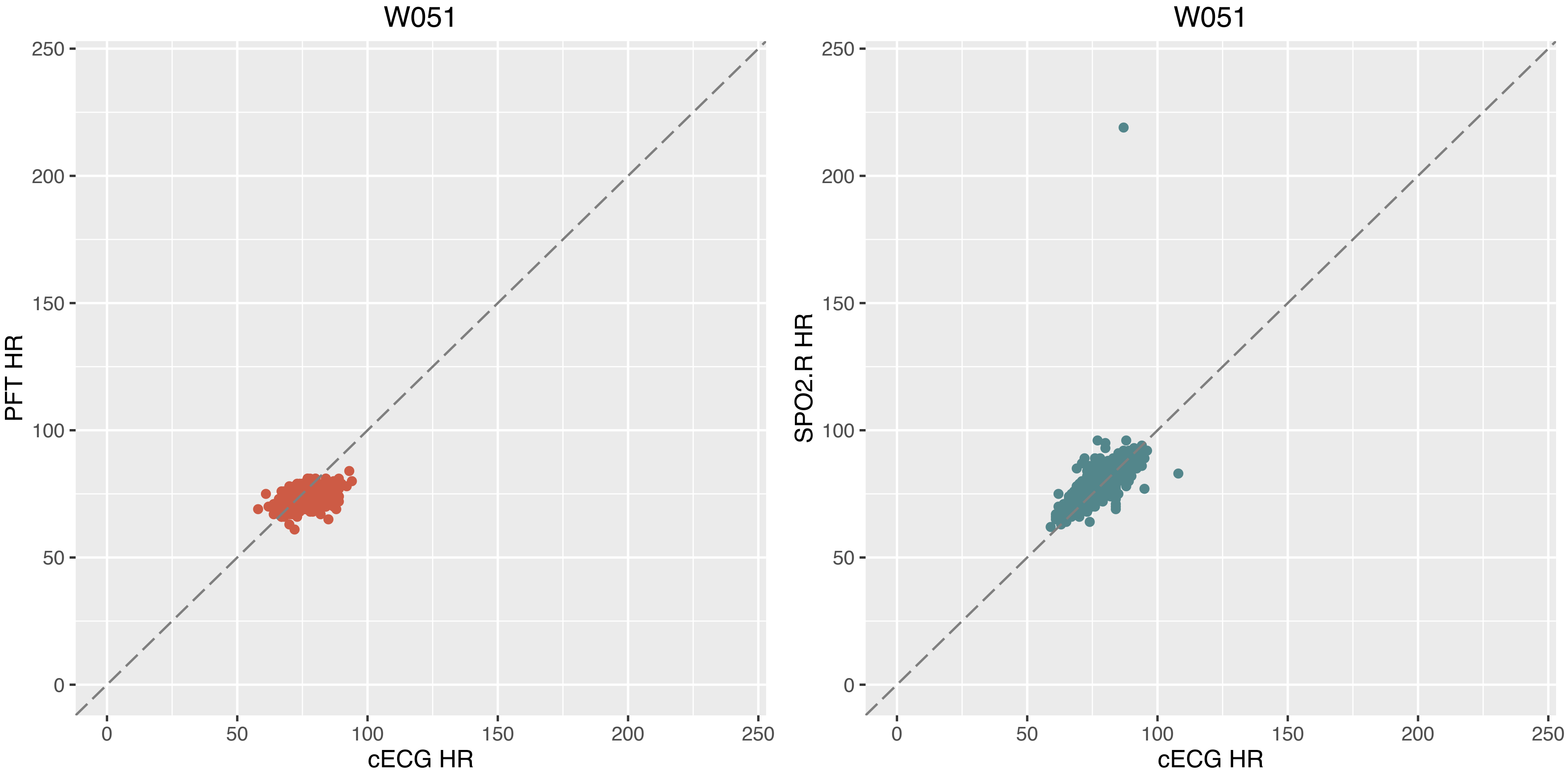

W051
PFT HR
cECG HR
W051
SPO2.R HR
cECG HR
0
50
100
150
200
250

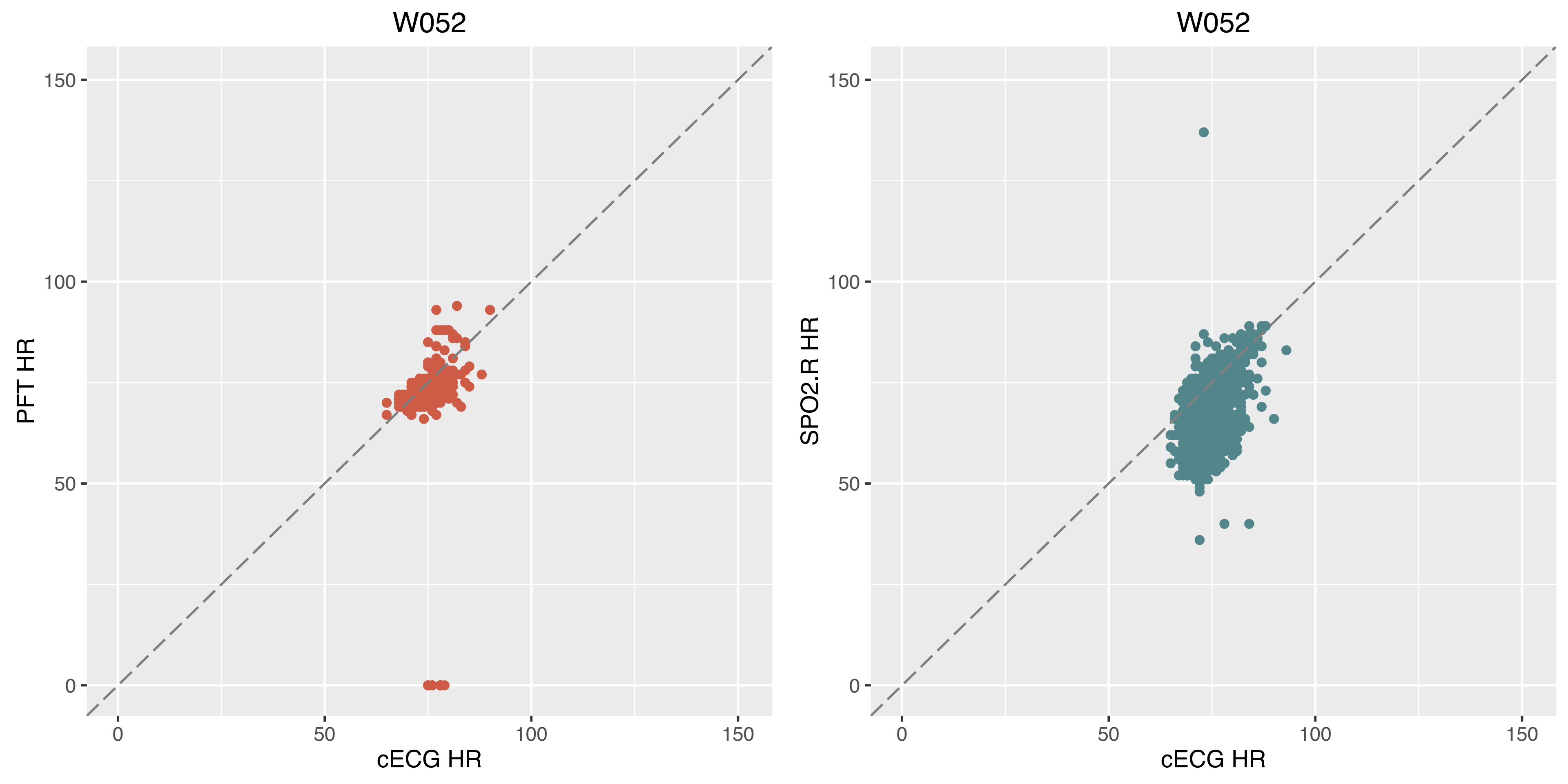
W052
PFT HR
cECG HR
W052
SPO2.R HR
cECG HR
0
50
100
150
